# Response to the Novel Corona Virus (COVID-19) Pandemic Across Africa: Successes, Challenges, and Implications for the Future

**DOI:** 10.3389/fphar.2020.01205

**Published:** 2020-09-11

**Authors:** Olayinka O. Ogunleye, Debashis Basu, Debjani Mueller, Jacqueline Sneddon, R. Andrew Seaton, Adesola F. Yinka-Ogunleye, Joshua Wamboga, Nenad Miljković, Julius C. Mwita, Godfrey Mutashambara Rwegerera, Amos Massele, Okwen Patrick, Loveline Lum Niba, Melaine Nsaikila, Wafaa M. Rashed, Mohamed Ali Hussein, Rehab Hegazy, Adefolarin A. Amu, Baffour Boaten Boahen-Boaten, Zinhle Matsebula, Prudence Gwebu, Bongani Chirigo, Nongabisa Mkhabela, Tenelisiwe Dlamini, Siphiwe Sithole, Sandile Malaza, Sikhumbuzo Dlamini, Daniel Afriyie, George Awuku Asare, Seth Kwabena Amponsah, Israel Sefah, Margaret Oluka, Anastasia N. Guantai, Sylvia A. Opanga, Tebello Violet Sarele, Refeletse Keabetsoe Mafisa, Ibrahim Chikowe, Felix Khuluza, Dan Kibuule, Francis Kalemeera, Mwangana Mubita, Joseph Fadare, Laurien Sibomana, Gwendoline Malegwale Ramokgopa, Carmen Whyte, Tshegofatso Maimela, Johannes Hugo, Johanna C. Meyer, Natalie Schellack, Enos M. Rampamba, Adel Visser, Abubakr Alfadl, Elfatih M. Malik, Oliver Ombeva Malande, Aubrey C. Kalungia, Chiluba Mwila, Trust Zaranyika, Blessmore Vimbai Chaibva, Ioana D. Olaru, Nyasha Masuka, Janney Wale, Lenias Hwenda, Regina Kamoga, Ruaraidh Hill, Corrado Barbui, Tomasz Bochenek, Amanj Kurdi, Stephen Campbell, Antony P. Martin, Thuy Nguyen Thi Phuong, Binh Nguyen Thanh, Brian Godman

**Affiliations:** ^1^ Department of Pharmacology, Therapeutics and Toxicology, Lagos State University College of Medicine, Lagos, Nigeria; ^2^ Department of Medicine, Lagos State University Teaching Hospital, Lagos, Nigeria; ^3^ Department of Public Health Medicine, Steve Biko Academic Hospital and the University of Pretoria, Pretoria, South Africa; ^4^ WHO Collaborating Centre for Social Determinants of Health and Health in all Policies, Pretoria, South Africa; ^5^ Charlotte Maxeke Medical Research Cluster, Johannesburg, South Africa; ^6^ Healthcare Improvement Scotland, Glasgow, United Kingdom; ^7^ Queen Elizabeth University Hospital, Glasgow, United Kingdom; ^8^ University of Glasgow, Glasgow, United Kingdom; ^9^ Department of Surveillance and Epidemiology, Nigerian Centre for Disease Control, Abuja, Nigeria; ^10^ Uganda Alliance of Patients’ Organizations (UAPO), Kampala, Uganda; ^11^ Institute of Orthopaedic Surgery “Banjica”, University of Belgrade, Belgrade, Serbia; ^12^ Department of Internal Medicine, Faculty of Medicine, University of Botswana, Gaborone, Botswana; ^13^ Department of Internal Medicine, University of Botswana and Department of Medicine, Princess Marina Hospital, Gaborone, Botswana; ^14^ Department of Biomedical Sciences, Faculty of Medicine, University of Botswana, Gaborone, Botswana; ^15^ Effective Basic Services (eBASE) Africa, Bamenda, Cameroon; ^16^ Faculty of Health and Medical Sciences, Adelaide University, Adelaide, SA, Australia; ^17^ Department of Public Health, University of Bamenda, Bambili, Cameroon; ^18^ Children's Cancer Hospital, Cairo, Egypt; ^19^ Pharmacology Department, Medical Division, National Research Centre, Giza, Egypt; ^20^ Pharmacy Department, Eswatini Medical Christian University, Mbabane, Eswatini; ^21^ Department of Psychology, Eswatini Medical Christian University, Mbabane, Eswatini; ^22^ Raleigh Fitkin Memorial Hospital, Manzini, Eswatini; ^23^ Pharmacy Department, Ghana Police Hospital, Accra, Ghana; ^24^ Department of Medical Laboratory Sciences, School of Biomedical and Allied Health Sciences, University of Ghana, Accra, Ghana; ^25^ Department of Medical Pharmacology, University of Ghana Medical School, Accra, Ghana; ^26^ Ghana Health Service, Pharmacy Department, Keta Municipal Hospital, Keta-Dzelukope, Ghana; ^27^ Pharmacy Practice Department, School of Pharmacy, University of Health and Allied Sciences, Hohoe, Ghana; ^28^ Department of Pharmacology and Pharmacognosy, School of Pharmacy, University of Nairobi, Nairobi, Kenya; ^29^ Department of Pharmaceutics and Pharmacy Practice, School of Pharmacy, University of Nairobi, Nairobi, Kenya; ^30^ Discipline of Pharmaceutical Sciences, School of Health Sciences, University of KwaZulu-Natal, Westville-campus, Durban, South Africa; ^31^ Independent Researcher, Mafeteng, Lesotho; ^32^ Pharmacy Department, College of Medicine, University of Malawi, Blantyre, Malawi; ^33^ Department of Pharmacy Practice and Policy, Faculty of Health Sciences, University of Namibia, Windhoek, Namibia; ^34^ Department of Pharmacology and Therapeutics, Ekiti State University , Ado-Ekiti, Nigeria; ^35^ Department of Medicine, Ekiti State University Teaching Hospital, Ado-Ekiti, Nigeria; ^36^ Department of Epidemiology, Graduate School of Public Health, University of Pittsburgh, Pittsburgh, PA, United States; ^37^ Department of Family Medicine, Steve Biko Academic Hospital and University of Pretoria, Pretoria, South Africa; ^38^ School of Pharmacy, Sefako Makgatho Health Sciences University, Pretoria, South Africa; ^39^ Department of Pharmacy, Tshilidzini Hospital, Shayandima, South Africa; ^40^ Eugene Marais Hospital, Pretoria, South Africa; ^41^ National Medicines Board, Federal Ministry of Health, Khartoum, Sudan; ^42^ Unaizah College of Pharmacy, Qassim University, Qassim, Saudi Arabia; ^43^ Faculty of Medicine, University of Khartoum, Khartoum, Sudan; ^44^ Community Medicine Council, SMSB, Khartoum, Sudan; ^45^ Department of Child Health and Paediatrics, Egerton University, Nakuru, Kenya; ^46^ East Africa Centre for Vaccines and Immunization (ECAVI), Kampala, Uganda; ^47^ Department of Pharmacy, University of Zambia, Lusaka, Zambia; ^48^ Department of Medicine, University of Zimbabwe College of Health Sciences, Harare, Zimbabwe; ^49^ Ministry of Health and Child Care, Harare, Zimbabwe; ^50^ London School of Hygiene and Tropical Medicine, London, United Kingdom; ^51^ Biomedical Research and Training Institute, Harare, Zimbabwe; ^52^ Zimbabwe College of Public Health Physicians, Harare, Zimbabwe; ^53^ Independent Consumer Advocate, Brunswick, VIC, Australia; ^54^ Medicines for Africa, Johannesburg, South Africa; ^55^ Community Health and Information Network (CHAIN), Kampala, Uganda; ^56^ Liverpool Reviews and Implementation Group, University of Liverpool, Liverpool, United Kingdom; ^57^ WHO Collaborating Centre for Research and Training in Mental Health and Service Evaluation, Section of Psychiatry, Department of Neuroscience, Biomedicine and Movement Sciences, University of Verona, Verona, Italy; ^58^ Department of Nutrition and Drug Research, Faculty of Health Sciences, Jagiellonian University Medical College, Krakow, Poland; ^59^ Strathclyde Institute of Pharmacy and Biomedical Sciences, University of Strathclyde, Glasgow, United Kingdom; ^60^ Department of Pharmacology, College of Pharmacy, Hawler Medical University, Erbil, Iraq; ^61^ Centre for Primary Care, Division of Population Health, Health Services Research and Primary Care, University of Manchester, Manchester, United Kingdom; ^62^ NIHR Greater Manchester Patient Safety Translational Research Centre, School of Health Sciences, University of Manchester, Manchester, United Kingdom; ^63^ Faculty of Health and Life Sciences, University of Liverpool, Liverpool, United Kingdom; ^64^ HCD Economics, The Innovation Centre, Daresbury, United Kingdom; ^65^ Pharmaceutical Administration & PharmacoEconomics, Hanoi University of Pharmacy, Hanoi, Vietnam; ^66^ Division of Clinical Pharmacology, Karolinska Institute, Karolinska University Hospital Huddinge, Stockholm, Sweden; ^67^ School of Pharmaceutical Sciences, Universiti Sains Malaysia, Penang, Malaysia

**Keywords:** COVID-19, Africa, prevalence, treatment, misinformation, health policy, unintended consequences, review

## Abstract

**Background:**

The COVID-19 pandemic has already claimed considerable lives. There are major concerns in Africa due to existing high prevalence rates for both infectious and non-infectious diseases and limited resources in terms of personnel, beds and equipment. Alongside this, concerns that lockdown and other measures will have on prevention and management of other infectious diseases and non-communicable diseases (NCDs). NCDs are an increasing issue with rising morbidity and mortality rates. The World Health Organization (WHO) warns that a lack of nets and treatment could result in up to 18 million additional cases of malaria and up to 30,000 additional deaths in sub-Saharan Africa.

**Objective:**

Document current prevalence and mortality rates from COVID-19 alongside economic and other measures to reduce its spread and impact across Africa. In addition, suggested ways forward among all key stakeholder groups.

**Our Approach:**

Contextualise the findings from a wide range of publications including internet-based publications coupled with input from senior-level personnel.

**Ongoing Activities:**

Prevalence and mortality rates are currently lower in Africa than among several Western countries and the USA. This could be due to a number of factors including early instigation of lockdown and border closures, the younger age of the population, lack of robust reporting systems and as yet unidentified genetic and other factors. Innovation is accelerating to address concerns with available equipment. There are ongoing steps to address the level of misinformation and its consequences including fines. There are also ongoing initiatives across Africa to start addressing the unintended consequences of COVID-19 activities including lockdown measures and their impact on NCDs including the likely rise in mental health disorders, exacerbated by increasing stigma associated with COVID-19. Strategies include extending prescription lengths, telemedicine and encouraging vaccination. However, these need to be accelerated to prevent increased morbidity and mortality.

**Conclusion:**

There are multiple activities across Africa to reduce the spread of COVID-19 and address misinformation, which can have catastrophic consequences, assisted by the WHO and others, which appear to be working in a number of countries. Research is ongoing to clarify the unintended consequences given ongoing concerns to guide future activities. Countries are learning from each other.

## Introduction

### COVID-19 and Key Risk Factors

The novel coronavirus named SARS-CoV-2 causing COVID-19 was first reported in Hubei Province in China in December 2019 (Guan et al., 2020; Li Q. et al., 2020; World Health Organisation, 2020a; Wu and McGoogan, 2020), and has subsequently spread to all continents (WHO, 2020a). COVID-19 is transmitted from person to person through respiratory droplets as well as directly through touching surfaces and other fomites (Chiang and El Sony, 2020; Huang et al., 2020; Perencevich et al., 2020; WHO, 2020b; World Health Organisation, 2020b). Initial observations from China suggested a case fatality ratio (CFR) of 2.3% (Wu and McGoogan, 2020). In their July 1, 2020 report, the World Health Organization (WHO) suggested a case fatality ratio (CFR) of 4.91% based on 10,357,662 confirmed cases worldwide and 508,055 recorded deaths (WHO, 2020a).

Increased morbidity and case fatality from COVID-19 is associated with a number of underlying health conditions, alongside male gender and older age. These include hypertension, cardiovascular disease (CVD), diabetes, obesity, chronic kidney disease, chronic obstructive pulmonary disease (COPD), and history of smoking and shortness of breath, as well as some immunosuppressive conditions and potentially blood type (Basu, 2020; Bode et al., 2020; CDC COVID-19 Response Team, 2020; Centre for Disease Prevention and Control, 2020; Di Lorenzo and Di Trolio, 2020; Dietz and Santos-Burgoa, 2020; Docherty et al., 2020; Du et al., 2020; Ellinghaus et al., 2020; Fang et al., 2020; Hamid et al., 2020; Huang et al., 2020; Inciardi et al., 2020; Khunti et al., 2020; Kirby, 2020; Kluge et al., 2020; Matos and Chung, 2020; Richardson et al., 2020; Ryan et al., 2020; Shah et al., 2020; Vardavas and Nikitara, 2020; Williams et al., 2020; Zheng Z. et al., 2020). Ethnicity, particularly for people of Black Afro-Caribbean origin alongside those from South Asia, has also emerged as a significant factor associated with a higher mortality risk versus the white population in Europe and the United States of America (USA) (Bambra et al., 2020; Khunti et al., 2020; Kirby, 2020; Pan et al., 2020; Pareek et al., 2020; Public Health England, 2020). However, it is currently not fully understood how ethnicity, poverty and deprivation, cultural and behavioral differences, as well as underlying health, interplay in morbidity or mortality risk especially with currently lower mortality rates in Africa at the end of June 2020 compared with a number of European countries and the USA (WHO, 2020a).

### Prevention and Treatment of COVID-19

Response activities to the COVID-19 pandemic typically included personal protection through physical distancing and hand washing coupled with respiratory precautions through face covering (Courtemanche et al., 2020; Ng et al., 2020; WHO, 2020b; WHO, 2020c; WHO, 2020d; World Health Organisation, 2020b). Increased testing and screening with contact tracing are fundamental to transmission control, and this has been variably combined with “locking down” of public places including educational establishments, retail outlets, factories and offices combined with closure of borders and quarantining suspected persons (Nussbaumer-Streit et al., 2020; Rajendran et al., 2020; WHO, 2020d). Case management of patients includes supportive care with supplementary oxygen coupled with ventilatory support for those most severely affected (Berlin et al., 2020; Marini and Gattinoni, 2020; Matos and Chung, 2020; Meng et al., 2020; Nigeria Centre for Disease Control, 2020a; World Health Organisation, 2020b). Depending on the threshold for hospital admission, it is estimated that up to 17% of hospitalized patients will require intensive care, with an appreciable proportion needing mechanical ventilation (World Health Organisation Europe, 2020; Docherty et al., 2020; Mahase, 2020a; Richardson et al., 2020). This is a concern even in high-income countries where there have been challenges with the availability of critical care beds, appropriately trained staff and personal protective equipment (PPE) (Di Lorenzo and Di Trolio, 2020; IHME, 2020a; IHME, 2020b; Lacobucci, 2020; Massonnaud et al., 2020).

An encouraging example of a positive response among low- and middle- income countries (LMICs) is Vietnam where multiple activities including extensive testing, contact tracing, and social distancing, under the government slogan “Fighting the epidemic is like fighting against the enemy”, have been successful in limiting the tranmission of COVID-19 with only 355 confirmed cases by the end of June 2020 and no recorded attributable mortality (Hall, 2020; Jones, 2020; Ministry of Health, VietNam, 2020; Pearson and Nguyen, 2020; Thai et al., 2020; WHO, 2020a).

Currently, there does not appear to be a cure for COVID-19; however, there are a number of medicines undergoing trials including antivirals, steroids, antimalarials, immunomodulators, and herbal medicines, some of which have shown positive findings although there have been concerns with the trial design of a number of studies (Das et al., 2020; ECDC, 2020a; Geleris et al., 2020; Luo L. et al., 2020; Mehta et al., 2020; Pradhan et al., 2020; Recovery Collaborative Group, 2020; Sanders et al., 2020; Scavone et al., 2020; Zhong H. et al., 2020).

The most promising therapeutic breakthrough to date has come from the UK-based adaptive randomised Recovery trial (ECDC, 2020a; Recovery Collaborative Group, 2020). The low cost corticosteroid dexamethasone has recently been shown to significantly reduce mortality in the most severely affected patients with COVID-19; patients with an oxygen requirement and those requiring ventilatory support (Recovery Collaborative Group, 2020). Other recent studies have also shown benefit from corticosteroids in critically ill patients increasing the number of days alive and free of mechanical ventilation as well as reducing mortality (Sterne et al., 2020; Tomazini et al., 2020). The prescribing of 6mg of dexamethasone daily for 10 days is now recommended for widespread use in the UK unless contraindicated for other reasons as well as endorsed by the National Institute of Health (NIH) in the USA (NIH, 2020a; Rees, 2020). However, currently optimal dosing and duration of dexamethasone in COVID-19 is unknown; consequently, current recommendations reflect those used in the Recovery trial.

Initial studies with remdesivir failed to demonstrate clinical benefit over placebo although these were underpowered (The Guardian, 2020a; BMJ Best Practice - Coronavirus disease 2019 (COVID-19), 2020; Wang Y. et al., 2020). More recently, larger scale studies conducted by NIH in the USA have shown encouraging results including a reduction in the time to recovery and a trend towards lower mortality (hazard ratio for death = 0.70) leading to the issuance of an Emergency Use Authorization by the US FDA as well as endorsement by the European Medicines Agency and the NHS in the UK (Beigel et al., 2020; EMA, 2020; NIH, 2020b; UK Medicines & Healthcare products Regulatory Agency, 2020; US Food and Drug Administration, 2020). However, further studies are needed before the prescribing of remdesivir can be fully endorsed.

However, much controversy surrounds the use of chloroquine and hydroxychloroquine with or without azithromycin for both the prevention and treatment of COVID-19. Following initial studies in China, coupled with the findings of Gautret et al. (2020) (Boulware et al., 2020; Das et al., 2020; Cortegiani et al., 2020; Gao et al., 2020; Gautret et al., 2020), its use was endorsed among a number of governments and medical societies (Bokpe, 2020; Channnel News Asia, 2020; East African, 2020; Rich, 2020; Sciama, 2020; Tilangi et al., 2020). However, the lack of a comparator arm in the initial studies has been heavily criticised coupled with concerns with side-effects including cardiac side-effects with hydroxychloroquine (International Society of Antimicrobial Chemotherapy, 2020; Gautret et al., 2020; ISAC/ Elsevier, 2020; Borba et al., 2020; Ferner and Aronson, 2020; Littlejohn, 2020) as well as reports of fatal overdoses (Abena et al., 2020; Das et al., 2020; GuruGamer, 2020; Nga et al., 2020; Politi, 2020). Recent studies have failed to demonstrate any clinical benefit for both the prevention and treatment of COVID-19 (Boulware et al., 2020; Geleris et al., 2020; Recovery Trial, 2020a; Rosenberg et al., 2020). The study by Mehra et al. (Mehra et al., 2020) also showed increased mortality with chloroquine or hydroxychloroquine; however, this has now been retracted subject to external auditing (Mehra et al., 2020; The Lancet, 2020). The European Medicines Agency now advises caution with the prescribing of hydroxychloroquine outside of clinical trials (European Medicine Agency, 2020a), and the WHO has halted the hydroxychloroquine arm in its ongoing Solidarity Trial with the NIH in the USA also halting the use of hydroxychloroquine in its studies (WHO, 2020e; ECDC, 2020a; NIH, 2020c). In South Africa, the South African Pharmacy Council has also warned against the misuse of hydroxychloroquine for the treatment of COVID-19, which builds on concerns from the regulatory agency given the lack of evidence (Masango, 2020; SAHPRA, 2020a).

The antiretroviral treatment (ART) lopinavir-ritonavir, which showed activity against MERS-CoV, has also been recommended for treating COVID-19 patients alongside arbidol (Cao et al., 2020; Kumar et al., 2020; Mitjà and Clotet, 2020; Zhong H. et al., 2020; Zhu et al., 2020). However, there have also been contrasting data regarding its effectiveness in COVID-19 patients, with most studies failing to show any clinical benefit including the UK Recovery study (Cao et al., 2020; ECDC, 2020a; Ford et al., 2020; Recovery Trial, 2020b). Consequently, lopinavir-ritonavir cannot currently be recommended for use outside of clinical trials and more recently the WHO has discontinued the lopinavir-ritonavir arm in the Solidarity Trial (WHO, 2020e). A recent study though by Hung et al. (2020) found that early triple antiviral therapy with interferon beta-1b, lopinavir/ritonavir and ribavirin alleviated the symptoms and shortened the duration of viral shedding and hospital stay compared with lopinavir/ ritonavir alone in patients with mild to moderate COVID-19 (Hung et al., 2020). Any positive findings though with combination therapies need confirmation before they can be endorsed. Studies regarding the potential effectiveness of nasal irrigation are also ongoing (US National Library of Medicine - ClinicalTrials.gov, 2020a; US National Library of Medicine - ClinicalTrials.gov, 2020b). This follows a pilot randomised study showing improvements in the duration of symptoms and viral shedding in patients with upper respiratory tract infections (Ramalingam et al., 2019), with a post-hoc re-analysis with a focus on those infected with coronaviruses also showing benefit (Ramalingam et al., 2020).

A number of other treatments focusing on the late inflammatory complications of COVID-19 (Gaborit et al., 2020; Jose and Manuel, 2020; Monteleone et al., 2020) are also under review. These include tocilizumab, which is widely used in rheumatoid arthritis and blocks Interleukin-6 (IL-6), a combination of emapalumab, anakinra and sarilumab (Clinical Trials Arena, 2020; Di Lorenzo and Di Trolio, 2020; Luo et al., 2020; Roumier et al., 2020; Toniati et al., 2020; Xu et al., 2020; Zhang C. et al., 2020), and interferons (Andreakos and Tsiodras, 2020; Prokunina-Olsson et al., 2020; Shalhoub, 2020; Tu et al., 2020). These are currently not recommended for use outside of clinical trials. Ongoing studies are also using the plasma from affected patients along with prior titration of neutralising antibodies (Bloch et al., 2020; ECDC, 2020a; UK Department of Health and Social Care, 2020; Tu et al., 2020; Wu et al., 2020), and anticoagulants for hypercoagulability states (Cunningham et al., 2020; Paranjpe et al., 2020; Shi et al., 2020; Tang et al., 2020; Xu et al., 2020). Again, these experimental therapies cannot be recommended until more clinical trial data becomes available.

There has also been controversy surrounding BCG vaccination as possible protection against COVID-19 (O'Neill and Netea, 2020; Schaaf et al., 2020). The WHO is warning against claims of effectiveness based on current ecological studies, with the South African Government and leading scientists also warning against diverting stocks away from neonatal vaccination programmes given concerns with shortages until more trial data becomes available (Medical Brief, 2020a; SAHPRA, 2020b; Schaaf et al., 2020; WHO, 2020f).

There are also concerns with the inappropriate use of medicinal plants to prevent and treat COVID-19, which such use particularly prevalent in some sub-Saharan African countries as this can cause more harm than good (Ekor, 2014; Liwa et al., 2014; Nkeck et al., 2020; Nordling, 2020; Yang, 2020). Safety fears are enhanced by the lack of data when used to prevent or treat COVID-19 along with other treatments that patients may be taking including antivirals, antibiotics as well as medicines for non-communicable diseases (NCDs) (Nkeck et al., 2020; Yang, 2020). We are aware that some herbal medicines are showing promise based on *in vitro* and small-scale clinical studies (Ang et al., 2020; Luo E. et al., 2020; Luo L. et al., 2020; Vellingiri et al., 2020; Yang, 2020; Zhang L. et al., 2020). However, their use outside of such studies is a concern until more data becomes available especially if their use causes delay in patients seeking appropriate care from healthcare professionals as their symptoms develop (Yang, 2020).

The controversies and issues surrounding a number of the treatments, the redaction of recent studies, as well as concerns with trial design in a number of studies (Bae et al., 2020; ECDC, 2020a; International Society of Antimicrobial Chemotherapy, 2020; ISAC/ Elsevier, 2020; Mehta et al., 2020; The Lancet, 2020), means it is essential that treatment recommendations should only be made once the results of robust trials are known (Council for International Organizations of Medical Sciences, 2020; Godman, 2020).

### Consequences of Lockdown and Other Measures to Prevent and Treat COVID-19

There are a number of unintended consequences arising from COVID-19. These include Governments diverting personnel and resources away from priority diseases including both infectious and non-infectious diseases. Reducing antimicrobial resistance (AMR) is a key activity across countries especially among LMICs as it increases morbidity, mortality and costs (Founou et al., 2017; Cassini et al., 2019; Hofer, 2019; Khan et al., 2019). However, diverting attention away from AMR, including ongoing efforts to reduce inappropriate self-purchasing of antibiotics, which is prevalent across many countries, as well as routine immunisation programmes for existing infectious diseases, will inevitably have a significant impact on future patient care (Ghosal and Milko, 2020; World Health Organization, 2020c; Health 24, 2020; Kalungia et al., 2016; Auta et al., 2019; Kalungia and Godman, 2019; Godman et al., 2020a; Hofman and Goldstein, 2020; Jerving, 2020; Lorgelly and Adler, 2020; Thornton, 2020; UN News, 2020; World Health Organisation, 2020d). Self-purchasing of antibiotics is a particular concern with the clinical presentation of COVID-19 overlapping with other infectious diseases including tuberculosis (TB), viral and bacterial respiratory tract infections, and pneumonia, making a differential diagnosis challenging exacerbated by limited diagnostic facilities in most communities (Godman et al., 2020b; Kasozi et al., 2020; Ongole et al., 2020). In hospitals, establishing antimicrobial stewardship programmes and other activities (Mendelson and Matsoso, 2015; Ghana Ministry of Health, 2018; Schellack et al., 2018; Anand Paramadhas et al., 2019; Afriyie et al., 2020; Godman et al., 2020b) can improve antibiotic prescribing where there is little evidence of bacterial co-infection (Godman et al., 2020a; Seaton, 2020; Zhou et al., 2020). Consequently, it is essential that such activities be introduced where necessary to guide future antibiotic prescribing among suspected COVID-19 patients (Seaton, 2020).

We are aware that the Ebola outbreak in Guinea, Liberia, and Sierra Leone between 2014 and 2016 resulted in as many people dying from HIV/AIDS, TB, and malaria as Ebola due to reduced access to health care (Parpia et al., 2016; Ghosal and Milko, 2020; Krubiner et al., 2020). There are also concerns that an appreciable reduction in the distribution of protective bed nets (75%) and medicines for treating malaria due to lockdown measures, combined with no media campaigns, could result in up to 18 million additional cases and up to 30,000 additional deaths in sub-Saharan Africa alone compared to 2018 (Cash and Patel, 2020; Krubiner et al., 2020; World Health Organisation, 2020c). Whilst the latest evidence suggests that HIV positive patients do not have a higher COVID-19 infection rate, or a significantly different disease course than HIV-negative individuals (Blanco et al., 2020; Guo et al., 2020; Härter et al., 2020; Tarkang, 2020), patients’ fears of contracting COVID-19 when they attend clinics, as well as limited access to health facilities and treatment during lockdown, will negatively impact on treatment and adherence to medicines as well as initial diagnosis (Africa News, 2020a; Chaiyachati et al., 2014; Mbuagbaw et al., 2015; Jerving, 2020; Krubiner et al., 2020; Tarkang, 2020). Medicine supplies can potentially be addressed through differentiated service delivery and other programmes (Jerving, 2020; Tarkang, 2020; Wilkinson and Grimsrud, 2020). Telemedicine and other technologies could also potentially help with consultations, with mobile technologies helping with tracking and tracing COVID-19 patients (Cohen et al., 2020; IOL, 2020; LinksCommunity, 2020).

There are also concerns that lockdown measures will negatively impact of the management of patients with non-communicable diseases (NCDs) (Kluge et al., 2020). This includes a lack of support and access to facilities to improve lifestyle management, monitor patient and regularly provide essential medicines (Kluge et al., 2020). Ongoing national plans to reduce morbidity and mortality due to NCDs, especially CVD and diabetes, across continents including Africa (Mensah et al., 2015; Amegah, 2018; Godman et al., 2020c; Godman et al., 2020d) could also be compromised by reduced access to medicines, cancelled or missed appointments due to patients' reduced access to facilities and fear of coming into contact with COVID-19 patients in healthcare facilities, and not following lifestyle advice (Kabale et al., 2020; Kluge et al., 2020; Nachimuthu et al., 2020). However, we are aware that governments are committing extra resources to try and minimise these unintended consequences (Ebrahim and Lakay, 2020). Issues relating to medical supplies can again be addressed by measures to extend prescription lengths (Al-Quteimat and Amer, 2020; Republic of South Africa Government Gazette, 2020; Lakay, 2020) as well as home delivery of medicines, with concerns with consultations potentially addressed through technologies such as telemedicine (Africa Health IT News, 2020a; LinksCommunity, 2020; Webster, 2020).

Other serious consequences include the exacerbation of mental health conditions during lockdown, the implications for frontline healthcare workers in terms of their health and wellbeing, and anxiety of citizens about their health (Brooks et al., 2020; Cullen et al., 2020; Endomba et al., 2020; González-Sanguino et al., 2020; Habersaat et al., 2020; Hwang et al., 2020; Kaufman et al., 2020; Li W. et al., 2020; Rajkumar, 2020; Ren et al., 2020; Wang C. et al., 2020a; Wang C. et al., 2020b; WHO, 2020g; Xiang et al., 2020). Patients with COVID-19 are also at increased risk of requiring psychotropic medicines because mild-to-moderate illness may result in adverse psychological effects from the diagnosis, from the symptoms, the need for forced isolation, any associated loss of income, and the potential risk of death. This might trigger new psychiatric symptoms or exacerbate underlying psychiatric conditions. In moderate-to-severe clinical situations, there is a risk that patients could develop altered states of consciousness such as hypo- or hyperkinetic delirium, which may require treatment with psychotropic medications (Reade and Finfer, 2014). In addition, some of the medical treatments that have been proposed for COVID-19 could contribute to onset or worsening of psychiatric symptoms. These include psychiatric disturbances with antivirals and steroids as well as depressive-dysphoric experiences with interferons (Tamam et al., 2003; Warrington and Bostwick, 2006; Manfredi et al., 2010; Kenna et al., 2011).

In addition, patients with existing mental illnesses may well have difficulties with accessing regular help during the pandemic unless pro-actively addressed (United Nations, 2020a; Yao et al., 2020). This includes continued access to medicines and treatments for those with long-term mental health conditions since sudden discontinuation should be avoided (WHO, 2020g). Worsening of mental health may be greater in patients with conditions such as schizophrenia with challenges in continuing active case management in the community during the pandemic as well as concerns with administering long-acting injections where pertinent, and performing regular blood tests in patients prescribed clozapine (Kozloff et al., 2020). Pro-active and timely support is needed for all patients with mental health conditions to avoid deteriorating conditions (Endomba et al., 2020; Salum et al., 2020; WHO, 2020g; Xiang et al., 2020). There are also reports of an increase in gender-based violence across countries, including African countries, as a result of lockdown measures, which also needs addressing going forward (Campbell, 2020; Chandan et al., 2020; Mahase, 2020b; SADC, 2020; United Nations., 2020b).

### Specific Challenges of COVID-19 in Africa

COVID-19 poses a particular challenge for the African continent because of existing high prevalence rates of other infectious diseases including human immunodeficiency virus (HIV)/ acquired immunodeficiency syndrome (AIDS), TB, cholera, and malaria, along with high rates of AMR and a disproportionate burden of poverty (UNAIDS, 2019; World Health Organisation, 2019; WHO, 2019a; Ataguba, 2020; Godman et al., 2020a; Simpson, 2020; United Nations Economic Commission for Africa, 2020a; WHO, 2020b; WHO, 2020c), with ongoing infectious disease initiatives appreciably challenged by COVID-19 (Mendelson and Matsoso, 2015; Ghana Ministry of Health, 2018; Godman et al., 2020a; Kowalska et al., 2020). The presence of multi-morbidity with NCDs will aggravate the situation further (Oni et al., 2015; So-Armah and Freiberg, 2018; Achwoka et al., 2019; Chang et al., 2019; Kansiime et al., 2019; Woldesemayat, 2020), with already high rates of CVD and diabetes a growing concern across Africa (Mensah et al., 2015; Godman et al., 2020b; Godman et al., 2020c).

Alongside this, there are ongoing challenges with available financial and human resources across Africa (Craven et al., 2020; Glied and Levy, 2020; Jayaram et al., 2020; Shepherd and van der Mark, 2020) including more limited availability of healthcare personnel and hospital beds including intensive care unit (ICU) beds (Murthy et al., 2015; Bates et al., 2018; Godman et al., 2019; El-Sadr and Justman, 2020; Godman et al., 2020c; Godman et al., 2020d; Martinez-Alvarez et al., 2020; United Nations, 2020b). Reports suggest there were just 1.8 hospital beds per 1,000 people across Africa before the pandemic, less than a third of those in France (Bavier, 2020). Oxygen is also not widely available and single room isolation can be a rarity making nosocomial transmission a concern. Overall, there appeared to be less than one ventilator and less than one ICU bed per 100,000 people in Africa compared with up to thirty times that number in the USA before the pandemic (Houreld et al., 2020), although there is regional variation (Murthy et al., 2015; El-Sadr and Justman, 2020; Shepherd and van der Mark, 2020; van den Heever, 2020). The availability of PPE across Africa was also a challenge in the early stages of the pandemic putting healthcare professionals at risk (Aljazeera, 2020; Lapolla et al., 2020; Le Roux and Dramowski, 2020; Saba and Jika, 2020). However, this is changing with increased local production of PPE, new local designs for face masks and ventilators (Bissada, 2020; Kenyatta University, 2020; Mining Review, 2020; Nyavor, 2020), as well as improved procurement processes enhanced by the recent launch of the pan-African medical supplies platform (South African Government, 2020a).

Most challenging though is over-crowding and lack of running water (and therefore a lack of hand washing) among the population, with an estimated 34% to 36% of people in Africa having no access to basic household washing facilities and 30% having only limited access (Bavier, 2020; El-Sadr and Justman, 2020; United Nations Economic Commission for Africa, 2020b). This is a particular concern among refugees, who require additional efforts to help prevent the spread of the virus including education on the rationale behind lockdown activities where pertinent (Associated Press, 2020; UNHCR, 2020).

Given these multiple issues and concerns, the UN Economic Commission for Africa (UNECA) initially estimated that the COVID-19 pandemic could potentially lead to 300,000 deaths across Africa and push 29 million people into extreme poverty (Bavier, 2020). In mid-April, the WHO warned that there could be up to 10 million cases of COVID-19 in Africa within six months (Aljazeera News, 2020a), rising to between 29 million and 44 million in the first year, with up to 190,000 deaths if containment measures failed (United Nations Africa Renewal, 2020). By the end of June 2020, there were over 303,000 COVID-19 cases among the WHO African countries with over 6000 deaths giving a CFR of 2.02% (WHO Regional Office Africa, 2020a). Consequently, absolute numbers of COVID-19 related deaths to date are still lower in Africa compared with other continents including the Americas and Europe (WHO, 2020a); however, this is changing as prevalence rates rise. There are also concerns with the reliability of the data among some of the African countries due to limited detection capacity and reliable tests as well as under-reporting of both deaths and prevalence rates (Ashly, 2020; Bruton and Edwards, 2020; Chiang and El Sony, 2020; Houreld and Lewis, 2020; McCaffrey, 2020; Mules, 2020; Shepherd and van der Mark, 2020; United Nations, 2020; WHO, 2020h). For instance, there has been no official data released from Tanzania since 8 May with the President stating the pandemic has largely been defeated despite concerns with truck drivers testing positive at borders and continued ongoing concerns with under-reporting (Houreld and Lewis, 2020; Mules, 2020; Mwai and Giles, 2020). Having said this, there have been concerns with the reliability of testing equipment (Aljazeera News, 2020b).

Other potential factors for currently lower prevalence rates and deaths in Africa compared with other continents could be a comparatively younger population as well as rapid instigation of lockdown and other measures. which build on experiences with other infectious diseases aided by the African Union, WHO Africa and the African Centre for Disease Control (CDC), as well as sensitivity of the virus to ambient temperatures (Africa CDC, 2020; Cash and Patel, 2020; EAC Secretariat, 2020; Lancet editorial, 2020; Pilling, 2020; WHO, 2020h; WHO, 2020i). The African CDC has been actively coordinating a strong multilateral response amongst African governments and other stakeholders towards COVID-19 building on the activities by the WHO in Africa and others (WHO Regional Office Africa, 2020b; WHO, 2020i; WHO, 2020j). The establishment of National Public Health Institutes among many African countries has also improved the response to public health threats (Nigeria CDC, 2018). Activities across Africa include developing laboratory expertise, training a volunteer health workforce, and risk communication. As mentioned, these built on Africa´s experience in dealing with other infectious diseases including Ebola, HIV, malaria and TB (El-Sadr and Justman, 2020; Nkengasong and Mankoula, 2020; Payne, 2020). The United Nations has also established a knowledge hub for COVID-19 to help African countries learn from each other (United Nations Economic Commission for Africa, 2020b). Testing has also appreciably increased in recent weeks across Africa (Burke, 2020) helped by Africa CDC and the WHO Africa, who have provided testing kits and training, with 48 African countries able to test for COVID-19 by 29 April 2020 compared with just two countries at the start of the pandemic (Ighobor, 2020; Simpson, 2020; WHO Regional Office Africa, 2020c).

Most African countries also rapidly instigated lockdown measures as well as social distancing to reduce the spread of the virus (Bavier, 2020; Dyer, 2020; United Nations., 2020b; United Nations Economic Commission for Africa, 2020a; WHO Regional Office Africa, 2020d). Similar to several African countries, Ghana early on began testing travellers on arrival in the country and isolating positive cases, as well as instigating testing among its citizens. Overall, Ghana has one of the highest testing rates in Africa having performed between 110,000 and 120,000 tests by the end of April 2020 (Di Caro, 2020). The Government of Ghana also established five key objectives to reduce the spread of the virus (Ghana News Agency, 2020; Zurek, 2020). Similar to most African countries, South Africa declared a national state of disaster in terms of section 27 (2) of the Disaster Management Act, 2002, and implemented a phased approach to lockdown with stage 5 introduced on March 27 2020, easing to stage 4 with effect from May 1 2020, which remains in place (Abdool Karim, 2020; South African Government, 2020b). Lockdown measures have now been eased among several countries following concerns about the economic and other issues including the potential for increased violence, whilst still maintaining an active response should the need arise (Moore, 2020; Shepherd and van der Mark, 2020; Tih, 2020). However, this situation is being actively monitored in case of a spike in prevalence rates.

Medicine shortages are an increasing concern across countries (Acosta et al., 2019) and are a particular issue in Africa where typically up to 94% of its medicines are imported (N Gage Consulting, 2017; Bavier, 2020; Dugmore, 2020), and supply issues can be exacerbated in countries with ongoing conflicts (ALCED, 2020). However, African countries are already taking steps to address concerns with shortages before and during the pandemic including suggesting potential alternatives (Chigome et al., 2019; Medical Brief, 2020b; Modisakeng et al., 2020; Review Online, 2020; Tomlinson, 2020). There are also ongoing programmes to address the quality of medicines across Africa. The potential for substandard and falsified medications is exacerbated when prices appreciably increase and shortages occur following endorsement of potential treatments for COVID-19 in the media (Abena et al., 2020; Haque et al., 2020; Kindzeka, 2020; Knott, 2020; World Health Organisation, 2020e). Government activities are likely to accelerate with the launch of the Lomé initiative placing falsified and substandard medicines on the highest political agenda with ongoing measures to strengthen the legal response to these medicines (WHO, 2020k).

COVID-19 has accelerated discussions on local pharmaceutical production in Africa (GhanaWeb, 2020a) with, for instance, the East African Community States seeking to support local production of essential medical products and supplies. Products include masks, sanitizers and ventilators, to address shortages in the region (EAC, 2020). In addition, several local companies have come forward to produce medical equipment and other products to manage the pandemic, and this will continue (Defy, 2020; Kaine and Nwokik, 2020; Nyaira, 2020). This is in line with the philosophy to develop regional hubs involving several African countries to address issues of economies of scale (Conway et al., 2020).

### Rationale Behind the Paper and the Objective

Given the multiple issues and challenges facing African countries, we believe there is an urgent need to consolidate knowledge of ongoing activities across Africa to address the COVID-19 pandemic and to understand its impact. Similarly, there is a need to evaluate the impact of re-directing activities away from the care of patients in other priority disease areas in Africa towards COVID-19 activities given the likely consequences on increased morbidity and mortality alongside the economic and social impact (Cash and Patel, 2020; Lancet editorial, 2020; Thornton, 2020).

We are aware that several regional financial institutions, including the African Development Bank and the African Export-Import Bank, have announced significant financial support for this purpose (African Development Bank, 2020; Okonjo-Iweala et al., 2020). The commitments of the World Bank and the International Monetary Fund also provide additional mechanisms, including suspending debts, to free resources towards the COVID-19 response (Ataguba, 2020). These measures should help address existing concerns regarding the financial impact of government measures including lockdown measures on the livelihoods of African citizens, with many currently working in the informal sector (Bonnet et al., 2019; Ataguba, 2020; Cash and Patel, 2020).

Containing the spread and impact of COVID-19, including the substantial economic impact, will though require a multipronged approach and co-operation among all key stakeholder groups going forward, including patients, with countries learning from each other (Ataguba, 2020). Ongoing activities include addressing the considerable misinformation about COVID-19 and potential treatments including vaccines and herbal medicines (Anna, 2020; Brennen et al., 2020; Forrest, 2020; Gallagher, 2020; Haque et al, 2020; Larson, 2020; Neil and Campbell, 2020; Newton and Bond, 2020; Nkeck et al., 2020; Serwornoo and Abrokwah, 2020; Smith, 2020; The Guardian, 2020; World Health Organisation, 2020f; Yang, 2020). The current controversies surrounding chloroquine/ hydroxychloroquine is just one example. As a result, there is a recognized need for scientific integrity and credibility when developing and discussing possible treatments for COVID-19 in line with recommendations from the Council for International Organizations for Medical Services (Council for International Organizations of Medical Sciences, 2020; Godman, 2020). This is to ensure future public and physician trust in treatment recommendations, which forms an integral part of effectively dealing with COVID-19 (Pitts, 2019; Abena et al., 2020; Goodman and Borio, 2020; Perry Wilson, 2020; Pitts, 2020; Rubin et al., 2020).

We are aware that early in the pandemic, Africa CDC, as well as WHO Africa and their partners, spearheaded efforts to train and sensitize African governments on the need to effectively counter and reduce the levels of misinformation (Budoo, 2020; Davis, 2020; Media Foundation West Africa, 2020). Their activities included training on risk communication and regular briefings to heads of state as well as relevant sectors of government. Additional efforts have also been provided by groups such as the International Alliance of Patients' Organizations (IAPO), which have developed resource hubs to provide reliable and updated information to mitigate against misinformation and promote preventative activities (International Alliance of Patients' Organizations, 2020).

Education among patients can also help reduce any stigma associated with COVID-19 for recovering patients and their families as well as any mistrust by the people in their governments (Adusei, 2020; AFP-JIJI, 2020; Habersaat et al., 2020; IFRC, UNICEF and WHO, 2020; Lubega and Ekol, 2020; United Nations, 2020b). We know that countries and governments have the potential to learn from the evidence base of HIV-related stigma interventions to help address such issues, as well as previous pandemics as seen with Vietnam (Logie, 2020; United Nations., 2020b; WHO, 2020a). Potential activities among patients and their organizations include active discussions around how stigma affects different communities, reflections on personal biases, and the instigation of institutional support programmes (Logie, 2020; He et al., 2020). This is important as the COVID-19 pandemic has already provoked stigmatisation and discriminatory behaviors against people of certain ethnic backgrounds as well as anyone perceived to have been in contact with or recovered from the virus (AFP-JIJI, 2020; GhanaWeb, 2020b; IFRC, UNICEF and WHO, 2020; ). Having said this, we have seen reasonable knowledge, attitudes and practices towards most aspects of COVID-19 among the public in China, Nepal, Pakistan and Paraguay although still room for improvement (Rios-González, 2020; Hayat et al., 2020; Singh et al., 2020a; Zhong B. L. et al., 2020). Surveys in the UK and US have also shown that participants generally had good knowledge about the main means of transmission and the common symptoms of COVID-19 (Geldsetzer, 2020). In Cameroon, the population is also aware of the disease and preventive measures (Nicholas et al., 2020). Further research is ongoing regarding why some people fail to adhere to suggested preventative measures, and we will be monitoring this (Chan et al., 2020).

Consequently, the objective of this paper is to summarise and consolidate our knowledge of current activities across Africa related to COVID-19 to help provide future guidance to all key stakeholder groups. This includes following up on clinical trial activities across Africa since an early concern was that few clinical trials were being conducted across Africa (Mabuka-Maroa, 2020; Roussi and Maxmen, 2020). This is beginning to change, building on examples in Burkina Faso, Kenya, Nigeria, South Africa and the WHO Solidarity Trial across several African countries (Anna, 2020; Coulibaly, 2020; Olafusi, 2020; WHO, 2020l). By doing so, we believe we can support African countries' efforts to continue to work together to tackle the pandemic, including jointly pursuing loan waivers to help protect against the financial consequences of COVID-19 (Phiri, 2020; President Republic of Kenya, 2020a).

This will be the first paper in this series as more information and findings become available. This builds on a recent systematic review regarding the importance of viral diseases in Africa, and the fact that different approaches will be needed across Africa to tackle COVID-19, depending on current circumstances (Chauhan et al., 2020; Mehtar et al., 2020; Shepherd and van der Mark, 2020).

## Methodology

We adopted a mixed methods approach. This initially involved conducting a narrative review of the published literature as well as papers awaiting publication and internet references known to the co-authors. We did not perform a systematic review. We were aware that some systematic reviews have already been conducted and published in this area including potential treatments despite the lack of data from robust clinical trials (Alqahtani et al., 2020; Castagnoli et al., 2020; Chowdhury et al., 2020; Cortegiani et al., 2020; Das et al., 2020; Ford et al., 2020; Huang et al., 2020; Nussbaumer-Streit et al., 2020; Sarma et al., 2020; Singh et al., 2020b; Vardavas and Nikitara, 2020; Zheng Z. et al., 2020). A number of the publications surrounding COVID-19 are also currently only available in pre-publication form and not peer-reviewed. Alongside this, much of the information regarding ongoing activities across Africa are from internet sources, and it is too early to assess the impact of these, especially as COVID-19 cases appeared later in Africa than in either China or Europe.

We also did not systematically review each paper for its quality using well-known scales such as the Newcastle-Ottawa scale or the Cochrane risk of bias tool as our emphasis was on contextualizing the findings rather than performing a systematic review (Marra et al., 2016; Almeida et al., 2018; da Silva et al., 2018; Ong et al., 2018; Saleem et al., 2019). However, the publications and internet sources were filtered by the co-authors to add robustness to the present paper and its suggestions.

In view of this, and to provide direction for the future, we supplemented information from the literature and internet sources with additional current information from co-authors across Africa. The co-authors include senior level personnel from governments and their advisers, lecturers and researchers from academia, and clinicians, as well as those involved with activities to enhance the rational use of medicine, undertake Health Technology Assessment (HTA), document medicine shortages, and involved with patient organizations from across Africa and wider. The co-authors were asked to provide information on the following themes or topics, where known, in their own country to supplement the ongoing literature:

- Details about the current epidemiology of COVID-19 including CFRs, national responses to date and an assessment of the effectiveness, if known
- The socio-economic impact as well as the impact on healthcare delivery in other priority disease areas including medicine and equipment shortages as well as attendance at clinics. This includes any information on the unintended consequences of COVID-19 in other disease areas if known
- How issues such as medicine shortages are being addressed and the implications for local manufacturing in the future
- Current medicines and other approaches to treating patients with COVID-19 as well as ongoing studies that address issues such as false claims and hopes. In addition, ongoing activities to address falsified and sub-standard medicines
- Key lessons for the future for all key stakeholder groups

The same questions were asked of each co-author, with country-specific replies typically consolidated where there were multiple authors in a country. The information was subsequently consolidated by two of the authors (OO and BG) and checked with each co-author during manuscript preparation to add robustness to the findings and suggestions.

We also documented ongoing technology innovations across Africa, which we believe is important as Africa seeks to become self-sufficient in the management of patients with COVID-19 and beyond.

Documented prevalence and mortality rates will typically be based on WHO date for consistency and reliability as there have been challenges with generating up-to-date data among a number of African countries due to the availability of testing facilities and testing kits, as well as concerns with some testing kits; however, as mentioned, this is now being addressed with the help of the WHO and others (Aljazeera News, 2020b; Burke, 2020; Simpson, 2020; UNICEF, 2020; WHO Regional Office Africa, 2020c).

The African countries chosen reflect a wide range of geographies and population sizes. We did not divide them into low- or middle-income countries as COVID-19 is likely to affect all African countries and they can learn from each other.

Statistical analysis of the different measures and initiatives and their possible impact on the epidemiology, morbidity and mortality rates, has not been undertaken as this is too early given rising prevalence rates across Africa. In addition, our principal aim was to provide a comprehensive analysis of the current situation across Africa, including the potential implications for other infectious and non-infectious disease areas within the African continent, to stimulate ongoing debates regarding potential future activities. This is important across Africa with high prevalence rates for both infectious and non-infectious diseases, We have successfully used this dual approach in previous publications to stimulate debate in important healthcare areas and situations to provide future guidance as countries seek to improve the quality and efficiency of their approaches to medicine use, including during pandemics (Godman et al., 2014a; Godman et al., 2014b; Godman et al., 2015; Ermisch et al., 2016; Bochenek et al., 2017; Ferrario et al., 2017; Moorkens et al., 2017; Godman et al., 2018; Godman et al., 2019; Godman et al., 2020b; Godman et al., 2020c; Godman et al., 2020d; Miljković et al., 2020).

## Findings

The findings and activities are divided into sections to meet the study objectives. These include documenting the epidemiology among a range of African countries principally based on epidemiology data provided by the WHO. The aforementioned precedes discussing ongoing strategies to limit the spread of COVID-19 as well as their subsequent impact on morbidity and mortality in populations across Africa.

The impact of COVID-19, including the unintended consequences on the healthcare system and patients, along with financial and socioeconomic issues, will be explored before documenting the subsequent impact on increased local production of pharmaceuticals and other supplies as well as any ongoing clinical studies and innovations across Africa aiming at improving future care. Finally, potential ways forward will be debated among all key stakeholder groups based on the experiences of the co-authors to provide future guidance. This includes key issues of shortages, unintended consequences, and misinformation as well as the role of patients and patient organizations in preventative measures and other interventions.

### Epidemiology

There is considerable variation in the number of recorded cases, deaths and CFRs across Africa following the first reported case on February 14, 2020 (United Nations., 2020b), with some countries yet to record their first deaths due to COVID-19 and some countries recording only a few deaths to date (**Table 1**), potentially reflecting different approaches and circumstances (**Tables 2** and **2A**). **Table 1A** in the **Data Sheet** gives further details of the epidemiology over time as well as recovered cases where known and documented.

**Table 1:** Current epidemiology of COVID-19 (up to 30 June 2020) across Africa unless stated.

Country	Population (2020)	Number of Positive Cases	Number of Deaths	Case Fatality Rate (%)
Algeria (WHO Regional Office Africa, 2020a)	43,851,044	13,907	912	6.6
Botswana (WHO Regional Office Africa, 2020a)	2,351,627	227	1	0.4
Burkina Faso (WHO Regional Office Africa, 2020a)	20,903,273	962	53	5.5
Cameroon (WHO Regional Office Africa, 2020a)	26,545,863	12,592	313	2.5
Democratic Republic of Congo (WHO Regional Office Africa, 2020a)	89,561,403	7,039	169	2.4
Egypt (WHO, 2020a)	102,334,404	68,311	2953	4.3
Eswatini (Swaziland) (WHO Regional Office Africa, 2020a)	1,160,164	812	11	1.4
Ethiopia (WHO Regional Office Africa, 2020a)	114,963,588	5,846	103	1.8
Ghana (WHO Regional Office Africa, 2020a)	31,072,940	17,741	112	0.6
Kenya (WHO Regional Office Africa, 2020a)	53,771,296	6,366	148	2.3
Lesotho (WHO Regional Office Africa, 2020a)	2,142,249	27	0	0.0
Malawi (WHO Regional Office Africa, 2020a)	19,129,952	1,265	16	1.3
Namibia (WHO Regional Office Africa, 2020a)	2,540,905	203	0	0.0
Nigeria (WHO Regional Office Africa, 2020a)	206,139,589	25,694	590	2.3
Rwanda (WHO Regional Office Africa, 2020a)	12,952,218	1,025	2	0.2
Senegal (WHO Regional Office Africa, 2020a)	16,743,927	6,793	112	1.6
South Africa (WHO Regional Office Africa, 2020a)	59,308,690	151,209	2,657	1.8
Sudan (WHO, 2020a)	43,849,260	9,258	572	6.2
Tanzania (WHO Regional Office Africa, 2020a)	59,734,218	509	21	4.1
Uganda (WHO Regional Office Africa, 2020a)	45,741,007	889	0	0.0
Zambia (WHO Regional Office Africa, 2020a)	18,383,955	1,594	24	1.5
Zimbabwe (WHO Regional Office Africa, 2020a)	14,862,924	591	7	1.2

NB: Population figures taken from Worldometer for Africa 2020 (Worldometer, 2020).

**Table 2:** Ongoing activities across Africa to help prevent the spread of COVID-19 including dates and examples.

Country	Closing Borders/ Travel restrictions	Quarantine Measures/ Testing measures	Lockdown Measures	Sanitary/ PPE measures	Directives on Management and misinformation
Algeria	17 March – closure of all land borders		13 March – Partial (capital) – full others with easing after that		
Botswana	Ongoing restrictions on air travel and strict border controls (20 to 28 March)	14-day quarantine introduced and scientists trained on specimen collection (23 March)	Yes – including closure of educational institutions (18 March) and general lockdown measures (2 April)	Mandatory use of face masks in public (May 1)	Yes – Directive to reduce misinformation with the potential for fines/ prison for abuse
Cameroon	18 March - closure of borders	3 March as well as active surveillance	18 March - including school closures and ban on gatherings	Yes- including encouraging hand washing and compulsory face masks in public (18 March onwards)	i) Establishing treatment centresii) Encouraging household production of face masks
Democratic Republic of Congo	24 March – closure of all borders and travel (International and domestic) remain suspended (6 June)	3 March – Quarantine measures	31 March – Curfew measures introduced with lockdown measures extended to 15 May (2 May)	Yes - Continued campaigns on prevention	Instigation Community Action Committees
Egypt	25 March – suspension of incoming passenger flights	Yes – early testing of suspected cases	25 March – Night time curfew and other lockdown measures	Yes – Increasing public awareness of preventative approaches	MoH developed standard treatment protocols
Eswatini(formerly Swaziland)	27 March – Closure of borders	27 March – Mandatory quarantining at borders and contract tracing introduced	27 March – i) Partial lockdown and social distancing introducedii) In addition, reduced numbers in omnibuses	Yes – distribution PPE to all health facilities	Yes – designating specific COVID-19 hospitals and fines or prison for spreading misinformation (27 March)
Ethiopia	Closing borders (22 March) and banning flights to more than 30 countries (20 March)	1 April – First testing labs (Northern Tigray Region)	16 March - closing schools and banning large gatherings	3 April – government and others helping with supply of PPE	
Ghana	22 March closure of all borders (quarantining from 16 March)	Enhancing testing facilities including suspected cases	16 March - Closure of schools and other measures including restrictions on burials	Early April - Mandatory masks in public places in a number of localities and patient education. 15 June - Compulsory masks	Dedicated hospitals and Government fast tracking testing of hand sanitisers
Kenya	25 March – suspending air travel and closing borders (mass testing and quarantining before this)	Increasing testing facilities with the help of increased local production and mandatory quarantining	13 - 19 March – lockdown and other measures introduced	Mandatory wearing of face masks and increased public education	i) Expansion of hospitals and ID units to deal with COVID-19ii) Development of treatment guidelinesiii) Active programmes to address misinformation
Lesotho	30 March – Borders closed	Private industry and NGO support for testing	30 March – lockdown measures introduced	NGO and industry support to purchase PPE	National response command centre provides guidance
Malawi	1 April - suspension of international flights and increased surveillance	Increasing the number of test facilities	18 April - lockdown measures proposed but suspended	Ongoing acquisition of PPE to meet demand	Instigation of isolation centres for COVID-19 patients and recruitment of more healthcare workers
Namibia	15 March – International flights banned and 24 March Foreign travellers banned		14 March – banning large gatherings; 15 March – schools closed and 27 March – lockdown measures introduced	Individuals expected to wear masks in public	Establishment of isolation units in public hospitals to tackle severe cases
Nigeria	23 March – closure of borders and ban on flights	Protocols for testing to increase rates	23 March – closure of schools and ban on gatherings; 30 March – ban on non-essential travel	Measures include hand sanitisation and mandatory wearing of masks (23 April)	Nigerian CDC developing protocols and case management and instigation of isolation centres
Rwanda	21 March – Ban on travel including air travel	Rapid testing – speeding up identification of cases	Mid-March – lockdown including unnecessary travel outside of homes prohibited	19 April - Wearing of masks compulsory early in the pandemic	Robots are helping in hospitals to address resource and other concerns
South Africa	26 March – complete travel ban introduced (eased 4 May)	April – mass screening introduced as well as active case-finding	26/ 27 March – Stage 5 lockdown introduced including school closures	Early April – use of PPE/ hand sanitisers regulated	i) Isolation centres establishedii) Spread of misinformation punishable with fines or prisoniii) Length of prescriptions increased
Sudan	Early March – Travel restrictions introduced – extended 20 April	Screening at point of entry and WHO/ UNICEF helping with testing materials as currently limited	Early/ Mid-March and extended - social isolation and school closures	WHO/ UNICEF helping with PPE – concerns with appreciably increased prices in shops	One isolation and treatment centre in the capital
Tanzania	April 12 – International flights suspended	23 March – 14-day quarantining from some countries	Yes – for large gatherings – however concerns that largely ignored		12 May – WHO helping to convert a commercial site into a 500-bedded treatment centre
Uganda	20 March – Closing borders and banning international travel	20 March – Mandatory quarantine at own expense for returning personnel	20 March – Lockdown measures implemented including suspending markets– slowly releasing Mid May	Advising the public regarding hygiene measures and mandatory wearing of masks	Task Force initiated (national, regional, district) to provide guidelines
Zambia	March - Temporary closing of borders especially with Tanzania	i) Initial response included a 14-day quarantine for travellers (Mid-March)ii) Mass screening where cases identified as well as contact tracing	Lockdown measures including social distancing and closure of educational establishments	March - Mandatory wearing of face masks in public places and active procurement of PPE	MoH training and recruiting health workers to help with case management3 designated laboratories to help with testing using PCR methods
Zimbabwe	31 March – closure of borders	31 March - All returnees quarantined for 21 days	30 March national lockdown – further extended 1 May	Local production of PPE and hand sanitisers to address shortages	i) Establishment of COVID-19 Treatment Centres at one ID Hospital in Harare and one in Bulawayoii) National Guidelines updatediii) Law passed with potentially prison for fake news

NB: CDC, Centre for Disease Control; MoH, Ministry of Health; NGO, Non-governmental organization; PPE, Personal Protective Equipment; WHO, World Health Organization.

### Ongoing Activities to Address COVID-19 and Their Impact

Different activities are ongoing across Africa to try and limit the impact of COVID-19. These are summarised in **Table 2**, with **Table 2A** in the **Data Sheet** giving additional details for those interested. As seen, prevention and treatment approaches remain broadly similar among the African countries, with a number of African countries combining approaches to provide joint guidance and updates on suggested activities, such as the East African Community (EAC Secretariat, 2020). There are concerns though with limited activity in some countries, e.g., Tanzania, as well as fears that if lockdown measures are released too early due to financial and resource issues for citizens and governments these will appreciably enhance future prevalence rates (Schroder et al., 2020).

### Health and Social Impact of COVID-19 Including Impact on Other Diseases

Measures to limit the spread of COVID-19 are having a considerable impact on other disease areas including both infectious and non-infectious diseases. **Table 3** contains details of the considerable healthcare and financial impact of COVID-19 as well as ongoing activities among African countries to address these. We will be examining unintended consequences in more detail in future research projects. Similarly, there are considerable financial and socioeconomic consequences which are also being addressed, with countries having the opportunity to learn from each other.

**Table 3:** Healthcare, financial, and socioeconomic impact of COVID-19 across Africa up to June 2020.

Country	Healthcare, Financial and Socioeconomic impact
Botswana	To date
	Healthcare services have not been seriously affected by the COVID-19; however, in ambulatory care there are fewer attendances at outpatients' clinics due to a bar on public transport There has been no noticeable impact on immunization programmes, prevalent infectious diseases (TB and HIV) and NCDs However, increased level of suspicion of COVID-19 in people presenting with respiratory illnesses
Cameroon	Healthcare concerns include: Cameroon is marked by violence in the North, the North West and the South West regions with a considerable number of internally displaced persons (IDPs). The COVID -19 pandemic constitutes an additional strain on the country's resources, which is a concern for the refugee populations. The implementation of both WHO-advised basic protective measures and national strategies have been slow especially in the crisis hit regions of the country due to other humanitarian crises with IDPs. However, with the WHO stating cloth masks should be used, this has made it easier for a majority of the population to be able to afford a mask. Treatment centers are currently lacking ventilators Cameroon reported three outbreaks of monkey pox between 2018 and 2020, several cholera outbreaks, measels, and polio between 2016 and 2020, with concerns for these infectious diseases with increasing focus on COVID-19 (Journal du Cameroun, 2020; MSF, 2019; Kindzeka, 2019a; Kindzeka, 2019b; Matengo, 2019; Outbreak News, 2020) However, International humanitarian agencies such is the Médecins Sans Frontieres (MSF) are at the forefront in the fight against COVID-19 in the crisis ridden region Financial and socioeconomic concerns and ongoing strategies to address this include: The imposed lock down has led to a drastic reduction in economic activities, shortfalls in tax and non-tax revenues with a looming budget deficit To help address concerns the government: Is embarking on delays in debt servicing and informing its creditors and international partners of its inability to meet current financial commitments Shutting down businesses, or other penalties, involved in selling basic commodities at increased prices or hoarding commodities to create artificial shortages to profiteer Despite these measures, over 92% of businesses, mostly small and medium size, are projecting appreciable downsizing of employees with the possibility of over 50% of companies collapsing in 3 months in the event of an extension of the current lockdown. Despite the desire to sustain schooling through on-line classes, the poor infrastructure, low internet penetration rate, and limited and fluctuating bandwidth has made this endeavour almost impossible for most academic establishments. Consequently, new approaches are needed
Democratic Republic of Congo	An ongoing concern is that the COVID-19 outbreak is creating significant additional pressure on an already overburdened health and social service delivery system in one of the world's poorest countries, which is exacerbating the vulnerabilities of the population (Adow, 2020; ReliefWeb, 2020a) This builds on an epidemic of measles, with the number of deaths to date appreciably higher than seen with COVID-19. Ducomble et al. in their recent paper documented 311,471 reported cases following the outbreak in 2019 with 6,045 reported deaths mainly among children under 5 (Ducomble and Gignoux, 2020) In addition, the Republic has had to deal with its tenth outbreak of Ebola, which has been ongoing since August 2018, the largest-ever outbreak reported in the Republic and the world's second largest, with a Public Health Emergency of International Concern declared on 17 July 2019 (ECDC, 2020b) There is ongoing testing against Ebola in the Republic, e.g., from 15 to 21 April 2020, an average of 2,037 alerts were reported and investigated per day, which is ongoing Of these, an average of 196 alerts were validated as suspected cases each day (WHO, 2020m) Overall, the WHO reported as of 21 April 2020, a total of 3461 cases of Ebola in the Republic, including 3,316 confirmed and 145 probable cases, of which 2,279 patients have died giving an overall case fatality ratio 66%. Out of the total confirmed and probable cases, 56% were female and 28% were children (WHO, 2020m)
Egypt	Healthcare concerns include: All outpatient services closed and services limited to emergencies including surgeries. There is also shortage of blood for transfusion and ICU beds for non-COVID-19 patients Many departments/hospitals were closed due to COVID-19 infections; however, have now reopened after decontamination procedures Shortage of PCR testing kits to adequately test the population Alongside this: Ongoing fear that the virus spreads from the dead bodies of infected COVID-19 patients leading to the abandonment of corpses of some infected doctor/patients despite undertaking all recommended preventive measures Stigma for medical doctors working in the diagnosis of asymptomatic cases due to a misconception that they are source for virus transmission Financial and socioeconomic concerns and ongoing strategies to address this include: Paid leave to government employees with special circumstances including: a) Women with children less than 12 years old, b) Employees who have documented chronic disease/s, c) Employees who have been in contact with COVID-19 patients and d) Employees who return from travel outside of Egypt Monthly financial support to people with irregular employment (OECD, 2020). Similarly, some NGOs have helped people in this category with supply of daily needs The Government on 20 April announced 100 billion EGP (USD 6.4 billion) to fund a comprehensive plan to combat the pandemic, half of which is allocated to the tourism sector (OECD, 2020)
Eswatini (Swaziland)	Health care concerns include: The designated isolation Hospital is currently not able to accommodate more than 16 patients. To address this, the Government has dedicated some hotels for quarantining; however, there are concerns with availability and patients are currently being quarantined at crowded homes. Hospitals are also being encouraged to make plans for isolating unstable suspected cases; however again concerns with capacity Partial closure of the major private hospital in Mbabane affecting care Very few ventilators were functional in government health facilities during the early stages of the pandemic with more needed as well as a shortage of hand sanitisers (although being addressed with local production) Adequate management of patients with HIV and TB in Eswatini, which has the highest percentage of people living with HIV worldwide, and a high prevalence of TB and with approximately 70% of all TB patients co-infected with HIV (WHO, 2019b; MSF, 2020). To help reduce visits to healthcare facilities, typically stable HIV patients now receive six-monthly refills of ARVs and among those with TB, medication is provided for one to three months depending on their health and drug availability (MSF, 2020) Given the risk of COVID-19, every effort should be made by hospitals and health centre staff to down-refer patients with stable NCDs to the primary care level. Patients with unstable diseases, recent emergencies related to their condition, and/or paediatric patients, are encouraged to come to hospital clinics where possible. All patients in primary care should only be administered with a one-month refill to enhance attendance and follow-up Encouragingly, vaccination programmes have not been stopped although due to travel restrictions there are missed appointments. There are also concerns with the availability of the influenza vaccine in retail pharmacies with out-of-stock situations reported from 23 April 2020 Financial and socioeconomic concerns and ongoing strategies to address this include: SRA-COVID-19 E90 million tax relief fund for small and medium enterprises Zimbabweans in Eswatini have donated E10000 towards the COVID-19 Fund Businesses in Eswatini have donated E24 million towards the COVID-19 Fund The Banks have announced that those individuals and companies that need short term financial support or relief can approach them and each application will be assessed on a risk-based approach. In addition, the Central Bank had made provisions for businesses to obtain loans at lower interest rates to help out (TradingEconomics, 2020) as well as encouraging electronic payments rather than cash transactions The Eswatini Government providing food assistance to the most vulnerable adversely affected by the COVID 19 pandemic, which will benefit over 300,000 individuals from 63,000 households across all four regions of the Kingdom (Government of The Kingdom of Eswatini, 2020) Such measures will help address the potential for an appreciable number of companies laying off staff due to the virus (Times of Swaziland, 2020)
Ghana	Healthcare concerns include: An outbreak of meningitis in the Upper West region of the country which has killed 33 people as of 4 April had not received the same attention as COVID-19 (Arhinful, 2020). By early May, 40 deaths had occurred and over 400 cases recorded, with mass vaccination now being contemplated Vaccination programmes including polio vaccination ceased early during the lockdown; however normal vaccination programs for babies born in hospitals are still being administered Currently no routine follow-up of patients with NCDs Psychologically, the lockdown has given a sense of imprisonment potentially enhancing the prevalence of mental health disorders. However, evening exercise and training under the cover of darkness has increased to help address this Public healthcare systems have focused mainly on COVID 19 cases during the lockdown, with a shift in funding to tackle COVID-19 to the detriment of other disease areas Financial and socioeconomic concerns and ongoing strategies to address this include: Free food distribution by the Government and NGOs to those in need, initially free transport for health workers, as well tax rebates for frontline workers. In addition, a 50% reduction in electricity and water tariffs and donation of salaries by Government officials as well as the Ghana Chamber of Mines to the COVID-19 National Trust Fund (Agence de Presse Africaine, 2020; Takyi-Boadu, 2020) Religious organizations have donated beds and monies to the National Trust Fund (Asamoah, 2020; Agbobli, 2020) The World Bank has provided US$100 million to assist in tackling the effects of the COVID-19 pandemic (The World Bank, 2020a) A recent publication regarding the cost-benefits of moderate social distancing in response to the COVID-19 pandemic in Ghana suggests that the costs outweigh the benefits. Consequently, the authors believe there is an urgent need to avoid escalating social distancing policies in Ghana, re-open schools and avoid reducing healthcare activities in other infectious diseases apart from COVID-19 as well as NCDs (Ghana Prorities Project, 2020)
Kenya	Healthcare concerns include: Reduced access to other healthcare services with most hospitals focusing on COVID-19, and currently no mechanisms for the management of chronic illnesses for those who are in quarantine. However, the rise in telemedicine approaches could help with identification and management of diseases (Africa Health IT News, 2020a; Africa Health IT News, 2020b; Bonner, 2020) Public health workers and community health volunteers have been mobilised and engaged to help with COVID-19 prevention A reduced flow of patients with other disease. To address this, online consultations have been promoted and some patients including those with cancer have received letters permitting them to travel and there has been an increase in home deliveries of medicines Some quarantine centres do not have adequate facilities for social distancing and infection prevention and control. In addition, concerns about the ability to pay for quarantining (Mutahi, 2020) COVID-19 positive cases have been reported in densely populated areas such as slums and refugee camp (Kakuma) putting strain on the public health programmes (Muraya, 2020) Response to other infectious diseases, e.g., cholera outbreak in Eastern and North Eastern Kenya - although systems have now been put in place to avert further loss of life (The East African, 2020). At least 194 lives have also been lost and many people displaced following heavy floods in Western Kenya in April putting further pressure on healthcare systems (Aljazeera News, 2020c) Antenatal and postnatal programmes have also been affected in some areas in Kenya Encouragingly, vaccination programmes are ongoing with no adverse impact reported to date. Financial and socioeconomic concerns and ongoing strategies to address this include: US$50 million donation from the World Bank to Kenya to tackle the COVID-19 pandemic (The World Bank, 2020b), with donations also from other countries (All Africa, 2020) Citizens earning less than US$300 per month have been exempted from paying taxes, with a general reduction in income tax. In addition, reduction in VAT, expedition of VAT payments and a general increase in cash flows especially for businesses (President Republic of Kenya, 2020b) Tax relief on telecommunication and other essential services as well as lowering of interest rates (President Republic of Kenya, 2020b) Food distribution to the vulnerable and low socioeconomic income groups Money retrieved from corruption cases will also be used to fund COVID-19 activities
Lesotho	Healthcare concerns include: There are only two hospitals designated for COVID-19 patients in Lesotho with limited number of beds and a limited number of ventilators for the entire country The government has though prepared a number of guest houses and hotels as quarantine facilities for people coming from other countries, who are screened for 14 days Screening has been scaled up at all of borders since there has been an influx of people entering the country through illegal borders. Screening is also being conducted in healthcare facilities and in the communities. Initially, there were inadequate laboratory services in Lesotho, and COVID-19 tests were undertaken in South Africa. However testing has now started in the National Reference Laboratory (The Reportor, 2020) with a substantial contact tracing program in place in response to the escalation of COVID-19 cases Currently, there are no reports on the impact of COVID-19 on the management of other infectious and non-infectious diseases, no identified medicine shortages, and vaccination programmes are progressing as normal. However, there are issues with accessing services due to shortages of public transportation and travel restrictions There has been considerable misinformation surrounding COVID-19 which may negatively hamper the government's efforts to contain the disease and prevent its spread. The Ministry of Health is working hard to deal with this and have quickly reprimand perpetrators Financial considerations: A concern is that prices of some basic food commodities, disinfectants and cleaning supplies have increased. However, the government has promised financial support where necessary
Malawi	Healthcare concerns include: Additional strain on the HCP workforce to cover all disease areas and not just COVID-19 patients Limited supply of PPE has put the lives of HCPs in danger, which resulted in a strike in many facilities across the country. This has been addressed by employing more HCPs and increasing their risk allowances (Africa News, 2020b), as well as making more PPE available. UNICEF has helped procure PPE (UNICEF Malawi, 2020) and the Jack Ma and Alibaba foundations have also helped to address supply issues (United Nations Malawi, 2020) The shortage of essential PPE for citizens has been compounded by an escalation of prices with the Competition and Fair Trading Commission (CFCT) of Malawi conducting price checks to help address concerns (Kumwenda, 2020) The shortage of hand sanitizers has been solved helped by the local production of hand sanitizers by many companies including institutions of higher learning in addition to supplies provided with the help of UNICEF. However, this has resulted in regulatory dilemma with limited availability of testing tools and protocols for such products. Measures are now in place in both the short term and long term to help solve this problem A major concern is that concentration on COVID-19 has diverted attention away from ensuring the availability of essential medicines for other disease areas Financial and socioeconomic concerns and ongoing strategies to address this include: Cash payments to the poor to help them survive the lockdown (Masina, 2020a) The United Nations launching an emergency appeal in Malawi. The UN believes US$140 million is needed to support the country's preparedness and response for the next six months (May onwards) and target the most vulnerable, which equates to 7.5 million people, nearly half of the country's population, with approximately 70% of the population living below the international poverty level if $1.90/ day (Masina, 2020b) The government has reduced the cost of fuel to mitigate against the increase in the cost of transport, which resulted from a reduction in the number of people boarding minibuses and private cars leading to an increase in the cost of transport and transporters overcharging passengers to make up for reduced numbers (Africa News Agency, 2020) Schools have been closed since 23 March 2020 (Tembo, 2020), with calls for alternative ways of engaging students including home reading and online learning; however concerns with limited resources including computers
Namibia	Healthcare concerns include a lack of hand sanitisers. Consequently, the School of Pharmacy at the University of Namibia was been tasked to produced hand sanitizers for the Ministry of Health and Social Services and other governmental and non-governmental organizations Measures to address financial and socioeconomic consequences include: Over 8 billion Namibian Dollars have been committed by the Government to stimulate the economy, provide social protection to families and support health sector response (ReliefWeb, 2020b) The government through the Ministry of Finance has also given financial support to small/individual business owners who depended on their daily income for survival. The package was ~USD 47 per person
Nigeria	Healthcare concerns include: Most secondary and tertiary public hospitals have shut down routine outpatient services with most elective surgeries on hold; however, facilities to interact with physicians when the need arises are being put in place including telephone services Re-organization of duty and call roasters to minimize the number of healthcare workers exposed to COVID-19 at any one time Immunization clinics are continuing to function as an essential service endorsing advice from the WHO (WHO, 2020n); however, utilization has reduced due to the lockdown Many patients suffering from non-COVID-19 conditions are currently unable to readily access care leading to concerns with increasing inactivity as well as increased use of complementary and alternative medicines Identification and management of NCDs has been hampered by reduced access to healthcare facilities, likely to impact negatively on medication adherence and outcomes, exacerbated by concerns with drug shortages Increasing levels of depression/ domestic violence as the lockdown and the lack of money continues Nigeria is currently experiencing an outbreak of Lassa fever with 979 confirmed cases as at 18^th^ April 2020 in 27 out of the 36 states leading to 188 deaths with a case fatality of 19.2% (Nigeria Centre for Disease Control, 2020b); however, receiving less attention than COVID-19 Financial and socioeconomic concerns and ongoing strategies to address this include: Tax rebates of 50%, suspension of import duties on medical equipment, medicines and PPE for treatment and management of COVID-19 for three months, effective 1 March 2020 N1 trillion (over US$2.5 billion) in loans to boost local manufacturing and production across critical sectors Distribution of cash to the most vulnerable poor and families in various communities. On April 1st, the Nigerian Electricity Regulatory Commission (NERC) suspended the payment of the new electricity tariffs scheduled to commence on April 2nd, citing poor electricity supply, wide metering gap and the impact of the COVID-19 pandemic as the basis for this gesture
South Africa	Healthcare concerns include: Elective surgeries have been stopped, with hospitals typically only dealing with emergencies There are ongoing programmes to educate the public on the importance of avoiding going to high level hospitals to reduce congestion. Overall, hospitals and clinics appear to be functioning although support staff such as HR, finance and some other allied staff have been sent home for lockdown to avoid transmission Access to health facilities has also become a major challenge due to lack of public transportation during lockdown To address concerns with the supply of medicines especially for patients with chronic conditions, a greater supply of medicines has been given to patients to avoid repeated visits to facilities and also includes prescriptions filled for up to four months or longer (Ho, 2020; Lakay, 2020) Currently, though there appears to be no shortages for possible treatments – helped by the lack of endorsement of medicines used to treat malaria or HIV to treat COVID-19 by the South African Health Products Regulatory Authority with concerns about stockpiling of medicines and lack of evidence (SAHPRA, 2020a). The South African Pharmacy Council also sought to discourage stockpiling of essential medicines (SAPC, 2020). However, shortages are likely to occur as the pandemic continues; with ongoing steps to try and reduce this address this (Medical Brief, 2020b; Review Online, 2020) There are concerns with the decrease in the extent of HIV testing as well as collection of ARV's due to fears of contracting COVID-19 (Africa News, 2020a) There are concerns with vaccination programmes with April 2020 showing a 22% decreases in the measles first dose and in fully immunised children under one year (Health 24, 2020), with UNICEF South Africa generally urging parents and caregivers to get their children immunized (UNICEF South Africa, 2020). Where immunisation services have been curtailed, the risks of children dying from a vaccine-preventable disease appreciably outweigh their risks of dying from COVID-19 (Abbas et al., 2020; Hofman and Goldstein, 2020) Increased anxiety among citizens as seen with protests in some Provinces against housing of citizens who were repatriated from China Initiatives to address financial and socioeconomic issues include: The World Bank, International Monetary Fund, BRICS New Development Bank and the African Development Bank have been approached and are working with the National Treasury on various funding projects (South African Government, 2020c) Tax relief, release of disaster relief funds, emergency procurement, wage support, funding to small businesses Social relief of distress grants given to those above 18 years of age who are (South African Government, 2002c; South African Government, 2020d): (i) Unemployed; not receiving any income or social grant; (ii) Not receiving any unemployment insurance benefit, any stipend from the National Student Financial Aid Scheme or resident in a government funded or subsidised institution Other initiatives include: Child support grant beneficiaries will receive an extra R300 in May and from June to October 2020 they will receive an additional R500 each month All other grant beneficiaries will receive an extra R250 per month for the next six months; a special Covid-19 Social Relief of Distress grant of R350 a month for the next 6 months will be paid to individuals who are currently unemployed and do not receive any other form of social grant or Unemployment Insurance Fund payment, and grants increased for a number of categories as of 1 April 2020 There has also been roll out food assistance through vouchers and cash transfers. The Department of Social Development has also partnered with the Solidarity Fund, NGOs and community-based organizations to distribute 250,000 food parcels across the country The Minister has also granted limited-time block exemptions to healthcare, banking, retail property and hotel sectors in respect of certain categories of agreements or practices between participants from the application of Section 4 (restrictive horizontal practices between competitors) and Section 5 (restrictive vertical practices between participants at various levels of the supply chain) of the Competition Act (Gounden et al., 2020)
Sudan	Healthcare concerns include: Difficulty with accessing Primary Health Care (PHC) for the treatment of NCDs including renal dialysis due to the partially paralysed activities in the public and private sectors Health centres/ hospitals closing down once a case of COVID 19 is suspected or confirmed with isolation of all health care providers who came into contact with the patient Inadequate supply of PPE for healthcare workers resulting in a good proportion on quarantine and a great reluctance among others to continue to work under the prevailing conditions. To help address this, there has been a shipment of 20,000 testing kits, 100,000 masks and 1,000 protective suits from the Jack Ma Foundation (OCHA, 2020), and UNICEF has mobilized US$370,000 for Infection Prevention and Control (IPC) supplies for use at points of entry to Sudan and in ambulances (OCHA, 2020) Economic hardship with long queues at bakeries and oil stations make social distancing difficult and meaningless Access to immunization services has been reduced in view of additional resources for COVID-19 The Federal Ministry of Health together with the WHO has developed a US$76 million countrywide COVID-19 preparedness and response plan to help combat concerns and the spread of the virus (OCHA, 2020) Financial and socioeconomic concerns and ongoing strategies to address this include: The harsh socioeconomic impacts of the pandemic are further compounded by the prevailing Sudanese Revolution and its attendant political and leadership crisis as well as Sudan still on the American State Sponsors of Terrorism list Sudan is on the list of countries expected to have severe food shortage from COVID-19 with already 61% of the population in South Sudan in a state of food crisis in 2019 (World Food Programme, 2020), with general agreement that the nation cannot sustain long-term lockdown and movement restriction with limited or no organized efforts from government to meet basic needs However, the World Bank on April 6 activated the Contingent Emergency Response Implementation Plan (CERIP) providing US$7.6 million in support of the coronavirus emergency response (World Bank, 2020) In addition (Dabanga, 2020): The Islamic Development Bank donated US$50 million to Sudan's Health Ministry for the Covid-19 response The United States announced on 27 March a donation of $8 million to Sudan. This assistance will primarily provide health-related support and supplies to bolster water, sanitation, and hygiene activities
Uganda	Healthcare concerns include: Difficulties with patients accessing ambulatory care facilities with restrictions on public and private transport alongside the curfew The Government has urged the public not to relax efforts to reduce HIV/ AIDS with the current high focus on COVID-19, with the aim of ending AIDS as a public health threat by 2030 (CGTN Africa, 2020), and some patients with HIV will struggle to obtain their medicines. This is being addressed to some extent by volunteers and the Ministry allowing community health workers to pick up medicines for patients (Athumani, 2020) Ensuring the safety of patients in health facilities and quarantine centres, enhanced by lack of testing facilities Measures to address financial and economic considerations include: The Ugandan president warning traders against raising the price of goods because of COVID-19 infection; however, Uganda currently has no price control and anti-profiteering laws (Kyeyune, 2020) Seeking support from the International Monetary Fund (IMF) as well as concessional loans amounting to US$100 million for 2019/ 2020 and US$90million 2020/ 2012 from the World Bank and seeking export promotion strategies to address concerns with high import rates (Xinhua, 2020a) Early May, the IMF approved a US$491.5 Million Disbursement to Uganda to help address the financial impact of the COVID-19 pandemic (IMF, 2020) Mobilisation of funds from the general public fund to help address the financial consequences
Zambia	Healthcare concerns include: HCPs are already overcommitted providing services to high patient workloads afflicted with other infectious diseases and NCDs. To address this, the Government has committed to recruiting an additional 8,000 health workers in 2020 to meet its target of enlisting 30,000 health workers by 2021 (Chilinda, 2020) Inadequate supply of commodities and equipment. To address this, the government has suspended custom duties and VAT on additional medical supplies used for COVID-19 coupled with donations from external organizations (Editor Food Business Africa, 2020; Lusakatimes, 2020; Xinhua, 2020b) Encouragingly, routine ambulatory and hospital care services in most parts of Zambia remain functional and routine vaccination programmes (especially for childhood illnesses) have not been disrupted in most parts of Zambia However, the financing of healthcare is already a concern and anticipated to be further constrained by the COVID-19 pandemic should further flare ups occur. This will negatively impact on care generally Financial and socioeconomic concerns and ongoing strategies to address this include: Epidemic Preparedness Fund amounting to ZMW57 million (approx. US$3.1 million); Cabinet approving a COVID-19 Contingency and Response Plan with a budget of ZMW659 million (approx. US$36.2 million) under the Disaster Management and Mitigation Unit (DMMU); and the Government mobilizing funds through the budget and engagement with international and domestic stakeholders (High Commission of the Republic of Zambia., 2020) Easing liquidity; waiver of tax penalties and interest on outstanding tax liabilities resulting from COVID-19; building new and sustainable value chains and engaging multilateral organizations including the IMF and the World Bank (EY Tax News Update, 2020)
Zimbabwe	Healthcare concerns include: Accessing healthcare for other conditions especially for chronic NCDs has become more challenging Patients with fever are also not getting appropriate timely care because health workers are not comfortable managing them until they know their COVID-19 status. This has resulted in some avoidable complications building on the malaria outbreak with over 226 deaths reported since 1 January 2020 (Haigh, 2020; OCHA Zimbabwe, 2020). Consequently, there is a need in malaria areas to differentiate malaria from COVID-19 There has been an alteration of drug dispensing protocols for patients with NCDs who are stable to provide these for 3-6 months to minimize hospital visits. The same principles also apply to patients on treatment for TB and HIV. However, Zimbabwe had a challenge with the shortage of medicines to treat patients with NCDs even before the COVID-19 pandemic. Most of the medicines for NCDs are typically procured out-of-pocket, with patients told to continue sourcing from private pharmacies if the hospital pharmacy does not have the medicine This is a potential shortage generally in supply and access to medicines especially with an increase in malaria cases amid the COVID-19 pandemic The plans for mass campaigns for TCV/IPV/HPV/Vitamin A that was scheduled for May 2020 has been put on hold due to the pandemic. Vaccination though is ongoing at facilities building COVID-19 guidance (social distance at facilities). However, this is a challenge when there is a need to ensure vaccination occurs in batches and where there are issues with public transport Measures to address financial and socioeconomic consequences include freezing of prices for basic commodities including food and income support

NB: AIDs, acquired immunodeficiency syndrome; ARVs, anti retrovirals; HCP, healthcare professional; HIV, human immunodeficiency virus; ICU, intensive care unit; NCDs, non-communicable diseases; PPE, personal protective equipment; TB, tuberculosis.

### Shortages of Medicines and Activities to Address This

There are shortages of medicines across Africa as most medicines are imported (**Table 3A**). We are likely to see local production increasing as part of future strategies to address ongoing shortages as a result of COVID-19, and we will be monitoring this in the future.

**Table 3A** in the **Data Sheet** contains details of ongoing shortages across Africa with countries starting to implement robust strategies to help deal with these in the future.

### Clinical Trial Activities

As mentioned, there has been limited clinical trial activity across Africa compared with higher income countries; however, this is beginning to change:

- In Botswana, there are currently no on-going clinical trials on COVID-19; however, the University of Botswana (a government-funded institution) has called for proposals on COVID-19 research, which was open until May 15, 2020.
- Egypt appears to be undertaking several clinical trials with currently 14 registered clinical trials granted expedited approval by review boards utilizing different medicines and treatment modalities (US National Library of Medicine, 2020).
- Currently there are no known clinical trials commissioned in Eswatini with no demonstrable commitment to commissioning COVID-19- related clinical research activities given the current pressure on resources.
- In Ghana, there currently appears to be no clinical trial research activities related to COVID-19. However, some medicinal plants with potential antiviral activity have been identified and submitted to the Centre for Scientific Research into Plant Medicine and Noguchi Memorial Institute for Medical Research for the commencement of studies/screening on their potential therapeutic anti-COVID-19/SARS-CoV-2 activities.
- Kenya is currently involved in WHO clinical trials for three medicines used in combating COVID-19. A vaccine clinical trial is envisaged in the next two or three months at the Kilifi KEMRI/Welcome Trust collaborating research site. The Institutional Review Boards have established electronic systems for protocol submissions and review feedback and expediting the review process (3-4 days for first response) to facilitate implementation.
- Three clinical trials have been registered in Nigeria but currently none have started recruiting (Global Coronavirus COVID-19 Clinical Trial Tracker, 2020). However, Nigeria has been included in the WHO SOLIDARITY trial with five treatment centres participating.
- A number of clinical trials are currently ongoing in South Africa for both pharmaceutical and non-pharmaceutical interventions with the Institutional Review Boards in South Africa having established electronic systems and expediting the review process to facilitate these. This includes the SOLIDARITY WHO trial with the South African research team being led by senior academics and clinicians from eight medical schools (Baleta, 2020) as well as the BCG vaccination trial programme for healthcare workers (NIH ClinicalTrials.gov, 2020a) and the South African Ox1Cov-19 Vaccine VIDA-Trial, the first clinical trial for a vaccine against Covid-19 in South Africa with the first patients enrolled the week of June 23, 2020 (NIH ClinicalTrials.gov, 2020b; Wits University, 2020a).
- Zimbabwe is also involved in the WHO SOLIDARITY Trial.

### Technical Innovations Regarding COVID-19 From Across Africa

There are many ongoing innovations across Africa to tackle COVID-19, and these are expected to continue. In Senegal, the Institut Pasteur, with support from the WHO, developed a US$1 COVID-19 Rapid Testing Kit to enable the country to undertake considerable testing with the government currently seeking approval for their kits to be used in other African countries (Kavanagh et al., 2020; LaMarca, 2020; Roberto, 2020). Scientists and industry partners in Ghana have also developed a test for COVID-19, a finger-prick blood test to rapidly detect antibodies, which is seen as beneficial among asymptomatic cases to reduce the spread (Nyavor, 2020).

Students at the Jomo Kenyatta University of Agriculture and Technology in Kenya developed a Mobile app to help with contact tracing, triaging and case management of COVID-19 patients (JKUAT, 2020a), with other students in Kenya also developing and making ventilators to address shortages (JKAUT, 2020b; Kenyatta University, 2020; University of Nairobi, 2020). A multidisciplinary team at Wits University in South Africa used their design and engineering skills to create face shields (Wits University, 2020b). A multi-disciplinary team from Honoris United Universities from across Africa, including doctors, engineers, and students, have developed a prototype for a new non-invasive respirator alongside face shields and splash protection masks, which can be quickly and affordably manufactured *via* 3D printing to address current shortages (Bissada, 2020).

Scientists in Uganda have also developed hands-free hand sanitisers and rapid testing kits with a team at Makerere University involved in manufacturing low-cost ventilators to bolster the country's capacity in case demand increases (Achan, 2020; Daily Monitor, 2020; School of Public Health Makerere University, 2020).

The Gauteng Department of Health in South Africa has introduced an app (Mpilo) based platform that supports improved service delivery and improved patient experiences in Gauteng Health facilities (Matshediso, 2020). The University of Pretoria in South Africa has also produced an interactive app with real-time data on COVID-19 infections to help with management (Gower, 2020). The governments in Egypt and Ghana have also recently launched apps to help with tracing people who have come into contact with COVID-19 positive patients as well as provide advice on management (El-Sabaa, 2020; Ministry of Communications, Republic of Ghana, 2020).

Scientists in Ghana have also successfully sequenced the genomes of SARS-COV-2 from 15 of the samples obtained from confirmed COVID-19 cases through active collaboration using the Next Generation Sequencing (NGS) Core and High-Performance Computing system (University of Ghana News Release, 2020). Whilst there have been some differences in the strains between countries, all 15 genomes typically resembled (>92% similarity) the original strain isolated from China (University of Ghana News Release, 2020). Scientists from the Nigerian Centre for Disease Control (NCDC) and others from across Nigeria, including from Lagos University Teaching Hospital, have also reported sequencing of the SARS-CoV-2 from the first confirmed case of COVID-19 in Nigeria (Pauloluniyi, 2020).

### Key Lessons Among Individual African Countries and Implications for the Future for Key Stakeholder Groups

**Tabel 4A** (**Data Sheet**) summarises key lessons learnt among a number of African countries going forward in the management of COVID-19 and other disease areas based on their experiences.

**Boxes 1**–**6** consolidate potential activities that could be undertaken across Africa to improve the future care of patients with COVID-19 building on existing activities (**Tables 2** and **2A**). However, we need to be mindful of a range of healthcare issues including the unintended consequences on both health and socioeconomic circumstances. This builds on **Table 3**, as well as **Tables 3A** and **4A**, for individual African countries combined with recommendations for all key stakeholder groups in Africa going forward in the management of both infectious and non-infectious diseases as well as clinical and economic issues surrounding fixed dose combinations (Godman et al., 2019; Godman et al., 2020a; Godman et al., 2020b; Godman et al., 2020c; Godman et al., 2020d).

Box 1 **National/ regional governments and authorities.**

**Leadership and Governance**

- Instigation of national/ regional pandemic planning. This includes the need for active surveillance with appropriate personnel and facilities as well as ensuring necessary healthcare workforce and structures including adequate supplies of PPE and other necessary equipment, and communication including all key stakeholder groups with reserved funds for such health emergencies. In addition, a phased approach to easing restrictions when pertinent (Habersaat et al., 2020). Such activities have worked well across countries (Jones, 2020; Wang C. J. et al., 2020)
- Development and implementation of occupational health and safety protocols, policies and legislative frameworks taking cognisance of the risks and mechanisms of COVID-19 infection. Such protocols, policies and legislative frameworks should be consistent with national and international workplace safety policies and guidelines. Effective enforcement and monitoring mechanisms should be put in place
- Planning should take account of the impact of COVID-19 on the mental health of patients and healthcare professionals as well as other priority disease areas and potential ways to address these. This requires a sound understanding of investment/ priority decision making and activities including HTA EBM principles
- Actively plan for misinformation building on the considerable levels of misinformation already seen by end June 2020 (Habersaat et al., 2020)
- Good communication and financial planning are vital to address concerns with any lockdown/ curfew measures especially in countries with high levels of informal sector workers/ high level of workers dependent on a daily wage for their survival
- Lead the development of care guidelines nationally as well as take part in the development of any Pan-African care guidelines. This builds on HTA/ EBM approaches
- Ensure longer term that all healthcare professionals have the necessary training to improve the management of patients with infectious diseases as well as likely co-morbidities especially among African patients. This includes instigating antimicrobial stewardship programmes as well as Infection Prevention Control groups in hospitals where these do not currently exist
- Enhance collaboration between African countries, especially those with shared borders. This builds on ongoing collaborations including the East African Community with their shared plans to tackle COVID-19 (EAC Secretariat, 2020)
- Explore telemedicine and other similar approaches to help with diagnosis of infectious diseases and address unintended consequences especially in patients with chronic NCDs (Africa Health IT News, 2020b; Bonner, 2020; LinksCommunity, 2020; Webster, 2020) as a consequence of any lockdown, alongside investigating the potential of robots for care delivery
- Continue with measures to reduce the extent of falsified and sub-standard medicines within Africa as well as measures to address concerns with misinformation. Alongside this, seek ways to enhance local/ regional production of medicines, PPE and other equipment to reduce reliance on imports

**Health Care Workforce (HCW)**

- Strengthen health care systems including ambulatory care systems as well as policies and initiatives to improve identification and management of COVID-19, as well as other high priority disease areas (capacity building)
- Instigate practices to ensure that essential health services offered by the private healthcare sector are not compromised
- Seek to address shortages of physicians where applicable including training and utilising of other healthcare professionals. This can include task shifting including a greater role for other professionals such as pharmacists (**Box 4**). As part of this, develop medium to long term epidemic/pandemic training for healthcare personnel to improve their preparedness and capacities for such health emergencies
- Enhance the professionalism of HCWs including initiatives to reduce inappropriate prescribing of antimicrobials where this occurs (Godman et al., 2020a; Khan et al., 2020)
- Strengthen community systems through capacity building of community owned resources including community extension health workers as well as voluntary healthcare workers as these are typically the first point of contact in communities
- Ensure HCWs continue to have access to PPE as well as additional training (where relevant) in dealing with pandemics as the safety of HCWs and patients is critical during pandemics
- Seek to share basic best practices across countries, including what works well in COVID-19 case management, infection, prevention and control.

**Financing/Socioeconomic issues**

- As most African currently countries rely on foreign input, governments need to find alternative sources of income in the future through agriculture and technology advances building on current innovations
- Minimize the socioeconomic impact through enhancing national solidarity and prioritization of social cohesion.
- Seek to protect vulnerable populations including the benefits and privileges of low-wage workers as well as address access to healthcare and support for all to reduce the burden that makes vulnerable people less able to follow public health directives
- Make it possible for small-scale business to survive periods of economic downturns occasioned by public health crisis. This can include facilitating the disbursement of interest loans to SMEs as well as reward local companies in the country with tax incentives/guaranteed bank loans to enable them recover post COVID-19

**Research Including Unintended Consequences for Both Infectious Diseases (including AMR) and non-infectious diseases**

- Promote and fund operational/national research on the impact of COVID-19 on infectious diseases (including AMR) and non-infectious diseases especially the unintended consequences (**Table 3**). This is particularly important in Africa where national programmes are just starting to address AMR as well as NCDs including CVD, diabetes, and mental health (Mendelson and Matsoso, 2015; Keates et al., 2017; Ghana Ministry of Health, 2018; Godman et al., 2020a; Godman et al., 2020b; Godman et al., 2020c)
- Continue to promote research into potential risk factors for morbidity and mortality among African patients given the differences that can exist between patients in Africa and those in high income countries
- Continue with research programmes aimed at mitigating against infectious diseases (including AMR) and non-infectious diseases across countries to add to the debate about the potential impact of unintended consequences
- Instigate research into new ways to manage patients with both infectious diseases and NCDs where there are constraints on patient access including better use of new technologies including telemedicine and consultations through the internet/ mobile telephones

NB: AMR, Antimicrobial Rsistance; CVD, Coronary Vascular Disease; EBM, Evidence-base medicine; HTA, Health Technology Assessment; NCDs, Non-communicable diseases; PPE, Personal Protective Equipment; SME, Small and Medium size enterprise.

Box 2 **Health technology assessment/ evidence based medicine.**

**Short Term**

- Continue reviewing emerging medicines that could be used in the management of COVID-19 to inform and update clinical protocols
- Disseminate findings *via* Ministry of Health and other websites to address current levels of misinformation
- Continue to undertake research for evidence-based interventions given the current controversies surrounding a number of suggested treatments for COVID-19, and encourage greater collaboration between governments, healthcare professionals and patient organizations researching and disseminating the findings
- Strengthen HTA capabilities especially when Ministries of Health are confronted with different potential interventions to address COVID-19 coupled with limited resources. This builds on existing initiatives across Africa including Ghana, Kenya and South Africa as well as the recent cost-benefit analysis on social distancing in Ghana and ICU versus care in general wards for patients with severe COVID-19 (Ghana Prorities Project, 2020; Hernandez-Villafuerte et al., 2016; Mueller et al., 2017; Hollingworth et al., 2018; Southern African Health Technology Assessment Society (SAHTAS), 2019; SAMRC, 2020)
- As part of this, upgrade research into priority decision making especially given the many competing healthcare needs among patients in Africa including those with infectious and non-infectious diseases as well as co-morbidities

**Longer Term**

- Seek to invest in personnel and resources to enhance HTA/ EBM capabilities within countries where currently limited personnel and resources to enable greater critique of evidence based alternatives
- Seek to establish HTA collaborations across African countries to avoid duplication building on experiences in Europe (European Observatory, 2016; KCE Report, 2017; Vella Bonanno et al., 2019)

NB: EBM, Evidence-base medicine; HTA, Health Technology Assessment.; ICU, intensive care unit.

Box 3 **Physicians (hospital and ambulatory care).**

**Short Term**

- Deployment of higher number of physicians where possible to help deal with the pandemic as well as other priority disease areas. Ensure physicians are motivated emotionally, mentally and financially to help deal with the pandemic especially given the likely impact of the virus on their mental health. Educational interventions and psychological support will be needed to ensure greater understanding of COVID-19 and its implications including better coping strategies. Psychological support could include development of support systems among all stakeholders and the general community.
- Physicians need to be provided with adequate PPE and training to address current fears, with hospitals provided with adequate logistical support (PPE, ventilators, medication, non-consumables)
- Embrace alternative ways of working including devolved responsibilities as well as the use of new technologies that help improve the care of all patients during pandemics
- Introduce greater opportunities for the training and re-training of ambulatory care physicians in the management of patients with COVID-19 and any co-morbidities given the challenges that can exist with differential diagnosis. As part of this, push for the instigation of antimicrobial stewardship programmes as well as IPC groups in hospitals where these do not currently exist
- Work with others to address concerns with misinformation as well as falsified and sub-standard medicines. In addition, concerns with potential shortages of medicines and equipment and alternative approaches

**Longer Term**

- Building on current experiences and collaborations on the best way to treat patients with COVID-19 and the aftermath including concerns with mental health disorders
- Focus on training of sufficient physicians from non-public health or circulatory medicine specialities to ensure a higher number of staff with transferable skills relevant to a pandemic that can be rapidly re-deployed
- There is an urgent need generally to employ more health personnel to augment current inadequacies across Africa. This includes exploring measures to attain and retain physicians

NB: IPC, Infection, prevention and control; PPE, Personal Protective Equipment.

Box 4 **Pharmacists (hospital and ambulatory care).**

**Short Term**

- Seek ways to ensure that medicines, or suitable alternatives, that are deemed helpful to patients with COVID-19 are routinely available coupled with medicines for other priority disease areas
- Work with Government agencies when falsified or sub-standard medicines are suspected to reduce their supply
- In hospitals, seek to ensure that hospital pharmacists are part of COVID-19 treatment teams including ASPs and IPC teams. This means that hospital pharmacist must keep abreast on current treatment research and protocols for COVID-19 and be familiar with EBM techniques given ongoing controversies (Al-Quteimat and Amer, 2020). In addition, should be provided with any necessary PPE including face shields and googles
- Hospital pharmacists should be involved in educating patients/staff on COVID-19 management especially on non-pharmacological and pharmacological interventions
- Where appropriate, pharmacist should be encouraged to prepare cost-effective WHO-recommended hand sanitizers for their facilities
- In the community, pharmacists have a vital role as they are often the first healthcare professional that patients contact regarding respiratory/ influenza diseases especially in countries with high patient co-payments, concerns with access to ambulatory healthcare facilities as well as issues of affordability to pay for a physician and their medicines (Markovic-Pekovic et al., 2017; Mukokinya et al., 2018; Adunlin et al., 2020; Al-Quteimat and Amer, 2020; Godman et al., 2020a). This includes encouraging self care/ hygiene measures including the supplying and wearing of masks and the need for regular hand washing as well as trying to protect patients against stock-outs of pertinent medicines (Al-Quteimat and Amer, 2020; Amariles et al., 2020; Haque et al., 2020; Ung, 2020)
- Community pharmacists can also discuss appropriate treatments including arguing against the need for antibiotics where this is a concern and encouraging appropriate referral where possible (Mukokinya et al., 2018; Amariles et al., 2020; Godman et al., 2020a)
- Community pharmacists and others can also push for extended supply of medicines where appropriate as well as help engage in discussions regarding adherence to medicines using different technologies especially given ongoing concerns with adherence to medicines without regular input from pharmacists and other professionals (Al-Quteimat and Amer, 2020; Kretchy et al., 2020; Zheng S. Q. et al., 2020).
- Pharmacists can also suggest alternative approaches during times of medicine shortages including potential OTC treatments (Cadogan and Hughes, 2020).
- They can also help improve stock controls to reduce potential shortages of key medicines with associated price increases, which is important in LMICs with high patient co-payments (Haque et al., 2020; Godman, 2020).
- Community pharmacists can also be involved in vaccination programmes given current concerns, as well as help administer a vaccine for COVID-19 when available with research suggesting that when pharmacists provide immunizations, they substantially increase the number of vaccinated people in the community (Hedima et al., 2020)

**Longer Term**

- Increase the training of Pharmacists at both hospital and community levels to be prepared/actively involved in management of COVID-19 patients as well as addressing unintended consequences including the use of technology to address issues with adherence
- Pharmacists should be part of appropriate relevant training and the development of emergency plans and workflows to deal with future pandemics and their consequences (Aruru et al., 2020)

NB: ASPs, Antimicrobial Stewardship Programmes; EBM, Evidence-based medicine; IPC, Infection, Prevention and Control; PPE, Personal protective equipment.

Box 5 **Other healthcare professionals in ambulatory and hospital care.**

**Short Term**

- Work with other professionals to identify optimal methods to deal with the care of patients with COVID-19 both in hospitals and in the community
- This includes making sure that healthcare professionals have the necessary PPE and equipment to deal with COVID-19 patients
- Seek to instigate training regarding the proper donning and removal of PPE during routine duties and in the management of suspected/positive COVID-19 cases if not in place
- Be part of ongoing plans to help improve the management of high priority disease areas outside of COVID-19 to make sure these patients are not neglected
- Work with necessary professionals to ensure the mental health of healthcare professionals and patients is not neglected during and post the pandemic. This includes helping to address issues of stigma

**Longer Term**

- Be part of elaborate plans to improve the management of patients with COVID-19 and other priority disease areas including re-distribution of activities to address shortages with physicians and other HCPs
- Seek to work with universities, companies and other groups to develop in-house/ in-country technologies to treat COVID-19 patients and be less reliant on imports in the future

NB: HCPs, Healthcare Professionals; PPE, Personal protective equipment.

Box 6 **Suggested activities among patients/patient organizations.**

**Short Term**

- Mobilize and engage with traditional, cultural, and other leaders who are respected and listened to in their communities to disseminate important health messages including hygiene and social distance measures associated with COVID-19 to help address current low levels of health literacy in a number of African countries. This includes addressing current misconceptions about the importance of using face masks, and how to use them appropriately, as many patients do not appreciate the need as well as helping acknowledge that some people with respiratory problems may have difficulties using masks for long, which needs to be effectively tackled
- Engage with various channels to help reassure COVID-19 patients that they pose no threat to the community after treatment, and should continue with their lives, as well as help address issues of stigma and discrimination against COVID-19 patients when these arise
- Work with government bodies to help address issues of misinformation about vaccines and treatments including misconceptions about vaccines and the consequences (Anna, 2020). In addition, work with all key stakeholder groups to help address the mental health issues that will arise from the current pandemic
- Patients organizations should work with regulatory authorities and professionals generally to strengthen ethical conduct and professionalism post the pandemic including promoting only evidence-based approaches across social and other media given concerns with the level of misinformation during the pandemic (Godman, 2020)
- Recovered COVID-19 patients can be used as ambassadors to sensitise the populace about the reality of the pandemic, and the need to avoid stigmatization
- Social media has proved useful and can provide an efficient means for patients and patient organizations to effectively communicate with their communities. However, this needs to be carefully managed to reduce the level of any misinformation and subsequent adverse consequences. This builds on activities with traditional, cultural, and other leaders
- Empower patients on medication safety and polypharmacy concerns as well as educate patients about self-management generally of their conditions especially patients with chronic NCDs in the absence of routine clinics
- Help explore possible telemedicine approaches to reduce reliance on clinic attendance, which in itself can be problematic outside of the pandemic if this involves extensive travel and waiting times

**Longer Term**

- Seek to develop patient-level data bases to routinely collect data on patients to help with future planning and care delivery
- Enhance the role of patient organizations within countries where pertinent as they can play a long-term role regarding advocacy and the spreading of information not only on COVID-19 but also other diseases and concerns. Patient organizations also have a key role generally to encourage changes in behaviors that promote good underlying health to support health outcomes and reduce the risk of severe symptomatic responses to a pandemic

A key area will be patients especially in view of their necessary compliance with any lockdown and social distancing activities as well as considerations for the unintended consequences of COVID-19. These include mental health considerations, managing maternal and childbirth challenges, the identification and management of other NCDs as well as other infectious diseases.

HTA will also be a crucial area especially given concerns with the extent of misinformation regarding COVID-19 and the implications. In addition, the need for governments to prioritise their limited resources across disease areas (Hatswell, 2020; Allen and Mirsaeidi, 2020), rather than devoting appreciable personnel and resources to one area to the detriment of others as seen currently with activities regarding COVID-19 (**Tables 2**, **2A**, and **3**). However, there are challenges including available personnel and resources as well as typically a greater focus on medicines among HTA units rather than other technologies (Hernandez-Villafuerte et al., 2016; Bijlmakers et al., 2017; MacQuilkan et al., 2018; Fasseeh et al., 2020). This is changing with studies in Ghana evaluating the cost-benefit of moderate social distancing policies (Ghana Prorities Project, 2020) and the cost-effectiveness of different prevention measures (Asamoah et al., 2020) as well as groups in South Africa HTA evaluating the cost-effectiveness of ICU versus care on general wards with patients with severe COVID-19 (SAMRC et al., 2020).

HTA and EBM will also be important in the development of clinical guidelines for Africa considering the costs involved with developing de-novo guidelines. Africa could certainly gain from a Pan-African guideline for the management of COVID-19 as knowledge grows to provide scientifically-based recommendations, similar to initiatives for NCDs (Okwen et al., 2019). Global evidence communities including the BMJ under its Best Practice series are already adopting this approach for knowledge generation, synthesis, and translation to facilitate evidence-based decisions (BMJ Best Practice - Coronavirus disease 2019 (COVID-19), 2020; McMaster University. COVID-END, 2020), and any Pan-African initiatives should build on this. Groups such as IAPO are also important with their resource hubs to provide robust, reliable, and updated information to help improve the care of patients (International Alliance of Patients' Organizations, 2020). In the meantime, we are beginning to see African countries taking steps to reduce the level of misinformation and this is likely to grow (Budoo, 2020; Davis, 2020).

## Discussion and Next Steps

We believe this is the first study to comprehensively document ongoing activities throughout Africa to tackle the health, financial and socioeconomic consequences of COVID-19. This is important given the high prevalence of both infectious and non-infectious diseases in Africa, the high number of people at or below the poverty level compared with other continents and higher income countries, as well as concerns with the lack of personnel, hospital beds and equipment (Murthy et al., 2015; UNAIDS, 2019; WHO, 2019a; World Health Organisation, 2019; Ataguba, 2020; Godman et al., 2020a; Godman et al., 2020b; Houreld et al., 2020; Martinez-Alvarez et al., 2020; Simpson, 2020; WHO, 2020c).

There have been different activities across Africa to prevent the spread of COVID-19 and reduce mortality rates (**Tables 2** and **2A**). African countries have broadly followed similar multiple approaches in terms of prevention including closing borders and instigating lockdown measures including travel restrictions, social distancing and testing. Closure of borders and quarantining along with other public health measures appear successful in reducing the spread of the virus (Nussbaumer-Streit et al., 2020), and this certainly appears to be the case among a number of the African countries. Encouragingly, to date there have been no or very few reported deaths among some of the African countries (**Tables 1** and **1A**), mirroring for instance the situation in other LMICs including Vietnam (WHO, 2020a). Encouragingly as well, there have been a number of innovations coming from the African continent to help with identification and management of patients with COVID-19, which includes local production of hygiene equipment as well as low-cost ventilators (Section 3.6). Alongside this, initiatives to enhance the production of medicines in Africa to reduce their reliance on imported medicines and other essential supplies. This bodes well with dealing with future pandemics in Africa. We will continue though to monitor prevalence and mortality rates across Africa as well as potential reasons for the limited mortality up to late June 2020, certainly when compared with a number of European countries and the USA, including potential issues around ethnicity and other factors.

However, extensive lockdown and other measures to help control the spread of the virus have been at a cost including the negative impact on other disease areas as well as financial and socioeconomic consequences (**Table 3**). This includes an increase in mental health disorders exacerbated by issues of stigma. **Table 3** shows clearly the need to strengthen or enhance existing healthcare infrastructures in many countries to respond to such pandemics and indeed promote better population health and underlying lifestyle behaviors. While good health system infrastructure does not guarantee better health outcomes it increases the opportunities for these to happen (Donabedian, 2005).

Groups such as IAPO are important to reduce any stigma associated with COVID-19 (IFRC, UNICEF and WHO, 2020; Lubega and Ekol, 2020). In the process of stigmatisation, people are being labelled, stereotyped, separated, and/or experience loss of status and discrimination because of a potential negative affiliation with the disease. Stigma can drive people to hide their illness to avoid discrimination, which potentially prevents them from promptly seeking help, and could discourage them from adopting healthy behaviors, which is concerning (Thornicroft et al., 2007). This is because such barriers could potentially contribute to more severe health problems, ongoing transmission, and difficulties with controlling infectious diseases. As a result, there are ongoing strategies to address these challenges including a greater role for patient organizations (Kaufman et al., 2020).

There are real concerns though if lockdown policies are eased too early due to financial and socioeconomic pressures (Schroder et al., 2020), and we will be following this up in future research projects. We will also be following up the unintended consequences of COVID-19 on other infectious and non-infectious disease areas as well as vaccination programmes. In the meantime, **Boxes 1**–**6** provide direction to African countries going forward as they start to ease their lockdown restrictions and address the unintended consequences of the pandemic.

### Limitations

We are aware that we have not undertaken a systematic review. This is because, as mentioned, a number of quoted papers have not been formally peer-reviewed and a great deal of information regarding country activities is currently only available on the internet. In addition, cases of COVID-19 started later in Africa than in China and Europe, and it is too early to formally assess the impact of polices. A number of clinical trials regarding possible treatments for COVID-19 have also only recently started.

However, we believe the strength of our paper lies in its comprehensive approach including ongoing and planned activities from across Africa and next steps from senior level personnel, which we will be building upon as more data becomes available. This includes activities against COVID-19 and future activities to re-balance the care of patients with other infectious diseases as well as NCDs.

## Conclusion

There have been multiple activities across Africa to try and reduce the spread of COVID-19 with its subsequent impact on morbidity and mortality with the help of international and national groups including the WHO and African CDC. This is despite concerns with available resources, personnel and equipment compared with higher income countries. To date, mortality rates appear appreciably lower than seen in a number of European countries and the USA possibly due to early lockdown and other measures including early closure of borders and quarantining of returning travellers, which is encouraging given initial concerns in Africa. However, infection rates continue to rise which is a concern especially as lockdown measures are being eased in a number of African countries. Encouragingly, multiple activities were typically introduced early across Africa building on the experiences with other infectious diseases, with countries continuing to learn from each other. However, there are a number of unintended consequences which are likely to result in increased morbidity and mortality of other infectious diseases and NCDs. Activities are ongoing to try and reduce the impact, and we will be monitoring these in the future.

## Orcid Numbers

Olayinka O. Ogunleye, orcid.org/0000-0002-8921-1909

Debjani Mueller, orcid.org/0000-0001-7796-982X

Nenad Miljkovic, orcid.org/0000-0003-1282-3883

Julius C. Mwita, orcid.org/0000-0002-5947-3684

Godfrey Mutashambara Rwegerera, orcid.org/0000-0002-5896-6065

Bene D Anand Paramadhas, orcid.org/0000-0002-8204-1417

Mohamed Hussein, orcid.org/0000-0003-0349-7262

Wafaa M. Rashed, orcid.org/0000-0001-7531-2840

Loveline Niba, orcid.org/0000-0002-5938-4913

Melaine Nsaikila, orcid.org/0000-0003-2335-3768

Adefolarin A. Amu, orcid.org/0000-0003-3212-2283

Daniel Afriyie, orcid.org/0000-0001-8859-3565

Seth K. Amponsah, orcid.org/0000-0001-7411-0972

Israel Sefah, orcid.org/0000-0001-6963-0519

Tebello Violet Sarele, orcid.org/0000-0002-5874-6599

Refeletse Keabetsoe Mafisa, orcid.org/0000-0001-9713-466X

Felix Khuluza, orcid.org/0000-0002-8334-0160

Dan Kibuule, orcid.org/0000-0002-6908-2177

Francis Kalemeera, orcid.org/0000-0002-4320-5087

Mwangana Mubita, orcid.org/0000-0002-8732-8644

Tshegofatso Maimela, orcid.org/0000-0002-8554-1329

Joseph Fadare, orcid.org/0000-0002-5641-1402

Johannes Hugo, orcid.org/0000-0002-9406-8801

Ibrahim Chikowe, orcid.org/0000-0002-8231-9140

Johanna C. Meyer, orcid.org/0000-0003-0462-5713

Enos M. Rampamba, orcid.org/0000-0002-3492-9104

Abubakr Alfadl, orcid.org/0000-0002-3014-1408

Elfatih M. Malik, orcid.org/0000-0002-8071-8092

Ombeva Malande, orcid.org/0000-0002-1840-7402

Aubrey Kalungia, orcid.org/0000-0003-2554-1236

Chiluba Mwila, orcid.org/0000-0003-0160-1222

Ioana D. Olaru, orcid.org/0000-0003-3392-9257

Trust Zaranyika, orcid.org/0000-0003-4363-7709

Blessmore V Chaibva, orcid.org/0000-0001-7858-3814

Nyasha Masuka, orcid.org/0000-0003-4653-8626

Corrado Barbui, orcid.org/0000-0003-1073-9282

Ruaraidh Hill, orcid.org/0000-0002-2801-0505

Tomasz Bochenek, orcid.org/0000-0001-9915-7267

Amanj Kurdi, orcid.org/0000-0001-5036-1988

Stephen Campbell, orcid.org/0000-0002-2328-4136

Thuy Nguyen Thi Phuong, orcid.org/0000-0001-7939-5276

Antony P Martin, orcid.org/0000-0003-4383-6038

Brian Godman, orcid.org/0000-0001-6539-6972

## Author Contributions

OOO, DB, JF, and BG developed the concept of the paper and undertook the initial literature review. OOO, JF, JMe, ACK, and BG developed the draft questionnaire. All authors subsequently contributed to the development of the paper including country activities as well as critiqued subsequent drafts before submission.

## Conflict of Interest

AM is employed by HCD Economics.

The remaining authors declare that the research was conducted in the absence of any commercial or financial relationships that could be construed as a potential conflict of interest. However, a number of them are employed by national or regional governments in Ministries of Health or are advisers to them.

## References

[B1] Abbas K. Procter S. R. van Zandvoort K. Clark A. Funk S. Mengistu T. (2020). Routine childhood immunisation during the COVID-19 pandemic in Africa: a benefit-risk analysis of health benefits versus excess risk of SARS-CoV-2 infection. Lancet Glob. Health (EPrint). 10.1016/S2214-109X(20)30308-9 PMC7367673 32687792

[B2] Abdool Karim S. S. (2020). The South African Response to the Pandemic. N Engl. J. Med. 382 (24), e95. 32469479 10.1056/NEJMc2014960 PMC7281717

[B3] Abena P. M. Decloedt E. H. Bottieau E. Suleman F. Adejumo P. Sam-Agudu N. A. (2020). Chloroquine and Hydroxychloroquine for the Prevention or Treatment of COVID-19 in Africa: Caution for Inappropriate Off-label Use in Healthcare Settings. Am. J. Trop. Med. Hyg. 102 (6), 1184–1188. 32323646 10.4269/ajtmh.20-0290 PMC7253100

[B4] Achan J. (2020). Dr. Wayengera: The man behind Uganda's COVID-19 testing kits. Available at: https://www.newvision.co.ug/new_vision/news/1518668/dr-misaki-wayengera-uganda-covid-19-test-kits.

[B5] Achwoka D. Waruru A. Chen T. H. Masamaro K. Ngugi E. Kimani M. (2019). Noncommunicable disease burden among HIV patients in care: a national retrospective longitudinal analysis of HIV-treatment outcomes in Kenya, 2003-2013. BMC Public Health 19 (1), 372. 30943975 10.1186/s12889-019-6716-2 PMC6448214

[B6] Acosta A. Vanegas E. P. Rovira J. Godman B. Bochenek T. (2019). Medicine Shortages: Gaps Between Countries and Global Perspectives. Front. Pharmacol. 10 (763). 10.3389/fphar.2019.00763 PMC6658884 31379565

[B7] Adow M. (2020). COVID-19 adds to DR Congo's multiple health crises - The healthcare system in one of the world's poorest countries is struggling to treat cholera, measles, ebola - and now coronavirus. Available at: https://www.aljazeera.com/news/2020/04/covid-19-adds-dr-congos-multiple-health-crises-200413180551574.html

[B8] Adunlin G. Murphy P. Z. Manis M. (2020). COVID-19: How Can Rural Community Pharmacies Respond to the Outbreak? J. Rural Health. 10.1111/jrh.12439 PMC7262086 32277726

[B9] Adusei A. (2020). Covid-19 is hitting communities in Ghana hard. BMJ.

[B10] AFP-JIJI (2020). Coronavirus stigma weighs heavily in sub-Saharan Africa. Available at: https://www.japantimes.co.jp/news/2020/05/20/world/social-issues-world/virus-stigma-sub-saharan-africa/#.XsYjk2hKjIU.

[B11] Africa CDC (2020). Coronavirus Disease 2019 (COVID-19). Available at: https://africacdc.org/covid-19/.

[B12] Africa Health IT News (2020a). Minet, clinic launch telemedicine service in Kenya. Available at: http://africahealthitnews.com/minet-clinic-launch-telemedicine-service-in-kenya/.

[B13] Africa Health IT News (2020b). Telemedicine Technology deplore in Kenya To Enhance Fight Against Covid-19. Available at: http://africahealthitnews.com/telemedicine-technology-deplore-in-kenya-d-to-enhance-fight-against-covid-19/.

[B14] Africa News Agency (2020). Malawi public transport operators raise fares to offset restrictions. Available at: https://www.iol.co.za/news/africa/malawi-public-transport-operators-raise-fares-to-offset-restrictions-45983229.

[B15] Africa News (2020a). HIV-positive people in SA skipping treatment over Covid-19 fears. Available at: https://www.africannewsagency.com/news-politics/HIV-positive-people-in-SA-skipping-treatment-over-Covid-19-fears-24770911.

[B16] African Development Bank (2020). President Adesina discusses the immediate and long-term impact of COVID-19 in Africa. Available at: https://www.afdb.org/en/news-and-events/multimedia/video/president-adesina-discusses-immediate-and-long-term-impact-covid-19-africa-35755.

[B17] Africa News (2020b). Malawi coronavirus: health workers protest over risk allowance. Available at: https://www.africanews.com/2020/04/21/malawi-coronavirus-health-workers-protest-over-risk-allowance//.

[B18] Afriyie D. K. Sefah I. A. Sneddon J. Malcom W. McKinney R. Cooper L. (2020). Antimicrobial Point Prevalence Surveys in two Ghanaian hospitals: opportunities for antimicrobial stewardship. JAC Antimicrob. Resist. 1–9. 10.1093/jacamr/dlaa001 PMC8210261 34222959

[B19] Agbobli S. (2020). ICGC donates GHC 100K towards COVID-19 trust fund. Available at: https://www.theghanareport.com/icgc-donates-gh%E2%82%B5100k-towards-covid-19-trust-fund/.

[B20] Agence de Presse Africaine (2020). Ghana Chamber of Mines donates $2m to Covid-19-Trust Fund. Available at: http://apanews.net/en/news/ghana-chamber-of-mines-donates-2m-to-covid-19-trust-fund.

[B21] ALCED (2020). Time Period:02/05/2019-02/05/2020. Available at: https://acleddata.com/dashboard/#/dashboard.

[B22] Aljazeera News (2020a). Africa coronavirus cases could hit 10 million in six months: WHO COVID-19 cases on the continent may shoot up from thousands to millions if models are accurate, UN health agency says. Available at: https://www.aljazeera.com/news/2020/04/africa-coronavirus-cases-hit-10-million-months-200417055006127.html.

[B23] Aljazeera News (2020b). Tanzania president questions coronavirus kits after animal test - President Magufuli says tests were found to be faulty after goat, sheep and pawpaw samples test positive for COVID-19. Available at: https://www.aljazeera.com/news/2020/05/tanzania-president-questions-coronavirus-kits-animal-test-200503174100809.html.

[B24] Aljazeera News (2020c). Kenya floods kill 194 people, displace tens of thousands - Government says 100,000 people displaced following torrential rain in the western part of the country. Available at: https://www.aljazeera.com/news/2020/05/kenya-floods-kill-194-people-displace-tens-thousands-200506133348867.html.

[B25] Aljazeera (2020). All you need to know about PPE. Available at: https://www.aljazeera.com/news/2020/04/ppes-important-200419071536635.html.

[B26] All Africa (2020). Kenya: Denmark Gives Kenya Sh320m to Boost COVID-19 Fund. Available at: https://allafrica.com/stories/202004050111.html.

[B27] Allen M. B. Mirsaeidi M. (2020). Health and Economy in COVID-19 Era: A Plan for Reconstituting Long-Term Economic Security. Front. Public Health 8 (235), 1–3. 32574304 10.3389/fpubh.2020.00235 PMC7266873

[B28] Almeida P. Silva T. B. C. de Assis Acurcio F. Guerra Junior A. A. Araujo V. E. Diniz L. M. (2018). Quality of Life of Patients with Type 1 Diabetes Mellitus Using Insulin Analog Glargine Compared with NPH Insulin: A Systematic Review and Policy Implications. Patient 11 (4), 377–389. 29322308 10.1007/s40271-017-0291-3 PMC6019415

[B29] Alqahtani J. S. Oyelade T. Aldhahir A. M. Alghamdi S. M. Almehmadi M. Alqahtani A. S. (2020). Prevalence, Severity and Mortality associated with COPD and Smoking in patients with COVID-19: A Rapid Systematic Review and Meta-Analysis. PLoS One 15 (5), 1–13. 10.1371/journal.pone.0233147 PMC7213702 32392262

[B30] Al-Quteimat O. M. Amer A. M. (2020). SARS-CoV-2 outbreak: How can pharmacists help? Res. Soc. Adm. Pharm. 10.1016/j.sapharm.2020.03.018 PMC7271257 32241695

[B31] Amariles P. Ledezma-Morales M. Salazar-Ospina A. Hincapié-García J. A. (2020). How to link patients with suspicious COVID-19 to health system from the community pharmacies? A route proposal. Res. Soc. Adm. Pharm. 10.1016/j.sapharm.2020.03.007 PMC7156146 32224133

[B32] Amegah A. K. (2018). Tackling the Growing Burden of Cardiovascular Diseases in Sub-Saharan Africa. Circulation 138 (22), 2449–2451. 30571350 10.1161/CIRCULATIONAHA.118.037367

[B33] Anand Paramadhas B. D. Tiroyakgosi C. Mpinda-Joseph P. Morokotso M. Matome M. Sinkala F. (2019). Point prevalence study of antimicrobial use among hospitals across Botswana; findings and implications. Expert Rev. Anti-Infe. Ther. 17 (7), 535–546. 10.1080/14787210.2019.1629288 31257952

[B34] Andreakos E. Tsiodras S. (2020). COVID-19: lambda interferon against viral load and hyperinflammation. EMBO Mol. Med. 12 (e12465), 1–4. 10.15252/emmm.202012465 PMC7267110 32333818

[B35] Ang L. Lee H. W. Choi J. Y. Zhang J. Soo Lee M. (2020). Herbal medicine and pattern identification for treating COVID-19: a rapid review of guidelines. Integr. Med. Res. 9 (2), 100407. 32289016 10.1016/j.imr.2020.100407 PMC7104236

[B36] Anna C. (2020). Protest vs. Africa's 1st COVID-19 vaccine trial shows fears. Available at: https://news.yahoo.com/protest-vs-africas-1st-covid-102207018.html.

[B37] Arhinful E. (2020). Pay attention to Meningitis outbreak in Upper West Region- Parliament directs Health Ministry. Available at: https://citinewsroom.com/2020/04/pay-attention-to-meningitis-outbreak-in-upper-west-region-parliament-directs-health-ministry/.

[B38] Aruru M. Truong H. A. Clark S. (2020). Pharmacy Emergency Preparedness and Response (PEPR): a proposed framework for expanding pharmacy professionals' roles and contributions to emergency preparedness and response during the COVID-19 pandemic and beyond. Res. Soc. Adm. Pharm. 10.1016/j.sapharm.2020.04.002 PMC7146711 32389631

[B39] Asamoah A. (2020). Methodist Church donated GHC 100,000 towards coronavirus fight. Available at: https://www.theghanareport.com/methodist-church-donates-ghc100000-towards-coronavirus-fight/.

[B40] Asamoah J. K. K. Owusu M. A. Jin Z. Oduro F. T. Abidemi A. Gyasi E. O. (2020). Global stability and cost-effectiveness analysis of COVID-19 considering the impact of the environment: using data from Ghana. Chaos Solitions Fractals 140, 110103. 10.1016/j.chaos.2020.110103 PMC7351453 32834629

[B41] Ashly J. (2020). Is Tanzania covering up the real number of coronavirus deaths? Suspicion over official COVID-19 numbers grows as critics accuse gov't of of failing to inform public on true extent. Available at: https://www.aljazeera.com/news/2020/05/tanzania-covering-real-number-coronavirus-deaths-200511054304751.html.

[B42] Associated Press (2020). Refugees Protest Under Coronavirus Lockdown in Rwanda. 17 April 2020. Available at: https://www.voanews.com/covid-19-pandemic/refugees-protest-under-coronavirus-lockdown-rwanda.

[B43] Ataguba J. E. (2020). COVID-19 Pandemic, a War to be Won: Understanding its Economic Implications for Africa. Appl. Health Econ. Health Policy 18 (3), 325–328. 32249362 10.1007/s40258-020-00580-x PMC7130452

[B44] Athumani H. (2020). Ugandan HIV-Positive Volunteer Goes Distance to Deliver ARVs. Available at: https://www.voanews.com/covid-19-pandemic/ugandan-hiv-positive-volunteer-goes-distance-deliver-arvs.

[B45] Auta A. Hadi M. A. Oga E. Adewuyi E. O. Abdu-Aguye S. N. Adeloye D. (2019). Global access to antibiotics without prescription in community pharmacies: A systematic review and meta-analysis. J. Infection 78 (1), 8–18. 10.1016/j.jinf.2018.07.001 29981773

[B46] Bae S. Kim M. C. Kim J. Y. Cha H. H. Lim J. S. Jung J. (2020). Notice of Retraction: Effectiveness of Surgical and Cotton Masks in Blocking SARS-CoV-2. Ann. Internal Med. 173 (1), 79. 10.7326/L20-0745 PMC7273461 32479106

[B47] Baleta A. (2020). COVID-19: SA to start enrolling patients in landmark WHO trial. Available at: https://www.spotlightnsp.co.za/2020/04/01/covid-19-sa-to-start-enrolling-patients-in-landmark-who-trial/

[B48] Bambra C. Riordan R. Ford J. Matthews F. (2020). The COVID-19 pandemic and health inequalities. J. Epidemiol. Community Health, jech-2020–214401. 10.1136/jech-2020-214401 PMC7298201 32535550

[B49] Basu S. (2020). Non-communicable disease management in vulnerable patients during Covid-19. Indian J. Med. Ethics V (2), 103–105. 32393447 10.20529/IJME.2020.041

[B50] Bates I. John C. Seegobin P. Bruno A. (2018). An analysis of the global pharmacy workforce capacity trends from 2006 to 2012. Hum. Resour. Health 16 (1), 3. 29325554 10.1186/s12960-018-0267-y PMC5765699

[B51] Bavier J. (2020). At least 300,000 Africans expected to die in pandemic: U.N. agency. Available at: https://www.reuters.com/article/us-health-coronavirus-africa-un/at-least-300000-africans-expected-to-die-in-pandemic-u-n-agency-idUSKBN21Z1LW.

[B52] Beigel J. H. Tomashek K. M. Dodd L. E. Mehta A. K. Zingman B. S. Kalil A. C. (2020). Remdesivir for the Treatment of Covid-19 - Preliminary Report. N Engl. J. Med. 10.1056/NEJMc2022236 32649078

[B53] Berlin D. A. Gulick R. M. Martinez F. J. (2020). Severe Covid-19. N Engl. J. Med. 10.1056/NEJMcp2009575 32412710

[B54] Bijlmakers L. Mueller D. Kahveci R. Chen Y. van der Wilt G. J. (2017). Integrate-HTA: A Low- And Middle-Income Country Perspective. Int. J. Technol. Assess. Health Care 33 (5), 599–604. 29103380 10.1017/S0266462317000927

[B55] Bissada A.-M. (2020). Coronavirus: 3D print of ventilators, easy and cheap to produce says lead researcher. Available at: https://www.theafricareport.com/28212/coronavirus-3d-print-of-ventilators-easy-and-cheap-to-produce-says-lead-researcher/.

[B56] Blanco J. L. Ambrosioni J. Garcia F. Martinez E. Soriano A. Mallolas J. (2020). COVID-19 in patients with HIV: clinical case series. Lancet HIV 7 (5), e314–e316 32304642 10.1016/S2352-3018(20)30111-9 PMC7159872

[B57] Bloch E. M. Shoham S. Casadevall A. Sachais B. S. Shaz B. Winters J. L. (2020). Deployment of convalescent plasma for the prevention and treatment of COVID-19. J. Clin. Invest. 10.1172/JCI138745 PMC7259988 32254064

[B58] BMJ Best Practice - Coronavirus disease 2019 (COVID-19) (2020). 7 May 2020. https://bestpractice.bmj.com/topics/en-gb/3000168/pdf/3000168/Coronavirus%20disease%202019%20%28COVID-19%29.pdf.

[B59] Bochenek T. Abilova V. Alkan A. Asanin B. de Miguel Beriain I. Besovic Z. (2017). Systemic Measures and Legislative and Organizational Frameworks Aimed at Preventing or Mitigating Drug Shortages in 28 European and Western Asian Countries. Front. Pharmacol. 8, (942), 1–24. 29403372 10.3389/fphar.2017.00942 PMC5779072

[B60] Bode B. Garrett V. Messler J. McFarland R. Crowe J. Booth R. (2020). Glycemic Characteristics and Clinical Outcomes of COVID-19 Patients Hospitalized in the United States. J. Diabetes Sci. Technol. 14 (4), 813–821 32389027 10.1177/1932296820924469 PMC7673150

[B61] Bokpe S. (2020). Watch out for counterfeit chloroquine on the market-FDA warns. Available at: https://www.theghanareport.com/fda/.

[B62] Bonner L. (2020). Kenya Opens First Telemedicine Center For Covid-19 Detection. Available at: https://www.axisimagingnews.com/news/kenya-opens-first-telemedicine-center-for-covid-19-detection.

[B63] Bonnet F. Vanek J. Chen M. (2019). Women and men in the informal economy: a statistical picture. Available at: https://www.ilo.org/wcmsp5/groups/public/—ed_protect/—protrav/—travail/documents/publication/wcms_711798.pdf.

[B64] Borba M. G. S. Almeida Val F. F. Sampaio V. S. Alexandre M. A. A. Melo G. C. Brito M. (2020). Chloroquine diphosphate in two different dosages as adjunctive therapy of hospitalized patients with severe respiratory syndrome in the context of coronavirus (SARS-CoV-2) infection: Preliminary safety results of a randomized, double-blinded, phase IIb clinical trial (CloroCovid-19 Study). MedRxiv preprint. Available at: https://www.medrxiv.org/content/10.1101/2020.04.07.20056424v2.full.pdf. 10.1101/2020.04.07.20056424

[B65] Boulware D. R. Pullen M. F. Bangdiwala A. S. Pastick K. A. Lofgren S. M. Okafor E. C. (2020). A Randomized Trial of Hydroxychloroquine as Postexposure Prophylaxis for Covid-19. N Engl. J. Med. 383 (6), 517–525 32492293 10.1056/NEJMoa2016638 PMC7289276

[B66] Brennen S. Simon F. Howard P. N. Nielsen R. K. (2020). Types, sources, and claims of COVID-19 misinformation. Available at: https://reutersinstitute.politics.ox.ac.uk/types-sources-and-claims-covid-19-misinformation.

[B67] Brooks S. K. Webster R. K. Smith L. E. Woodland L. Wessely S. Greenberg N. (2020). The psychological impact of quarantine and how to reduce it: rapid review of the evidence. Lancet 395 (10227), 912–920. 32112714 10.1016/S0140-6736(20)30460-8 PMC7158942

[B68] Bruton B. Edwards N. (2020). Barriers to mass testing for COVID-19 in Africa. Available at: https://www.atlanticcouncil.org/blogs/africasource/barriers-to-mass-testing-for-covid-19-in-africa/.

[B69] Budoo A. (2020). In Africa, government attempts to fight misinformation are also limiting freedom of expression. Available at: https://www.niemanlab.org/2020/05/in-africa-government-attempts-to-fight-misinformation-are-also-limiting-freedom-of-expression/.

[B70] Burke J. (2020). It's just beginning here': Africa turns to testing as pandemic grips the continent. Available at: https://www.msn.com/en-gb/news/world/its-just-beginning-here-africa-turns-to-testing-as-pandemic-grips-the-continent/ar-BB13de1l?ocid=spartandhp.

[B71] Cadogan C. A. Hughes C. M. (2020). On the frontline against COVID-19: Community pharmacists' contribution during a public health crisis. Res. Soc. Adm. Pharm. 10.1016/j.sapharm.2020.03.015 PMC7270164 32245691

[B72] Campbell A. M. (2020). An increasing risk of family violence during the Covid-19 pandemic: Strengthening community collaborations to save lives. Forensic Sci. Int.: Rep. 2 100089. 10.1016/j.fsir.2020.100089 PMC7152912 38620174

[B73] Cao B. Wang Y. Wen D. Liu W. Wang J. Fan G. (2020). A Trial of Lopinavir-Ritonavir in Adults Hospitalized with Severe Covid-19. N Engl. J. Med. 382 (19), 1787–1799. 32187464 10.1056/NEJMoa2001282 PMC7121492

[B74] Cash R. Patel V. (2020). The art of medicine - Has COVID-19 subverted global health? Lancet 395, 1687–1688. 32539939 10.1016/S0140-6736(20)31089-8 PMC7200122

[B75] Cassini A. Hogberg L. D. Plachouras D. Quattrocchi A. Hoxha A. Simonsen G. S. (2019). Attributable deaths and disability-adjusted life-years caused by infections with antibiotic-resistant bacteria in the EU and the European Economic Area in 2015: a population-level modelling analysis. Lancet Infect. Dis. 19 (1), 56–66. 30409683 10.1016/S1473-3099(18)30605-4 PMC6300481

[B76] Castagnoli R. Votto M. Licari A. Brambilla I. Bruno R. Perlini S. (2020). Severe Acute Respiratory Syndrome Coronavirus 2 (SARS-CoV-2) Infection in Children and Adolescents: A Systematic Review. JAMA Pediatr. 10.1001/jamapediatrics.2020.1467 32320004

[B77] Centre for Disease Prevention and Control (2020). People Who Are at Higher Risk for Severe Illness. Available at: https://www.cdc.gov/coronavirus/2019-ncov/need-extra-precautions/people-at-higher-risk.html?CDC_AA_refVal=https%3A%2F%2Fwww.cdc.gov%2Fcoronavirus%2F2019-ncov%2Fspecific-groups%2Fpeople-at-higher-risk.html.

[B78] CDC COVID-19 Response Team (2020). Preliminary Estimates of the Prevalence of Selected Underlying Health Conditions Among Patients with Coronavirus Disease 2019 - United States, February 12-March 28, 2020. Morbid. Mortal. Wkly. Rep. 69 (13), 382–386. 10.15585/mmwr.mm6913e2 PMC7119513 32240123

[B79] CGTN Africa (2020). Uganda urges public on HIV/AIDS spread amid COVID-19 pandemic. Available at: https://africa.cgtn.com/2020/05/06/uganda-urges-public-on-hiv-aids-spread-amid-covid-19-pandemic/.

[B80] Chaiyachati K. H. Ogbuoji O. Price M. Suthar A. B. Negussie E. K. Bärnighausen T. (2014). Interventions to improve adherence to antiretroviral therapy: a rapid systematic review. AIDS 28 (Suppl 2), S187–S204. 24849479 10.1097/QAD.0000000000000252

[B81] Chan D. K. C. Zhang C. Q. Josefsson K. W. (2020). Why People Failed to Adhere to COVID-19 Preventive Behaviors? Perspectives from an Integrated Behavior Change Model. Infection Control Hosp. Epidemiol., 1–6. 10.1017/ice.2020.245 PMC7253766 32408917

[B82] Chandan J. S. Taylor J. Bradbury-Jones C. Nirantharakumar K. Kane E. Bandyopadhyay S. (2020). COVID-19: a public health approach to manage domestic violence is needed. Lancet Public Health. 5 (6), e309 32401709 10.1016/S2468-2667(20)30112-2 PMC7252171

[B83] Chang A. Y. Gómez-Olivé F. X. Manne-Goehler J. Wade A. N. Tollman S. Gaziano T. A. (2019). Multimorbidity and care for hypertension, diabetes and HIV among older adults in rural South Africa. Bull. World Health Organ 97 (1), 10–23. 30618461 10.2471/BLT.18.217000 PMC6307505

[B84] Channnel News Asia (2020). Malaysia still using hydroxychloroquine to treat COVID-19 patients; health ministry monitoring side effects. 26 May 2020. Available at: https://www.channelnewsasia.com/news/asia/covid-19-malaysia-hydroxychloroquine-still-using-treatment-12771770.

[B85] Chauhan R. P. Dessie Z. G. Noreddin A. El Zowalaty M. E. (2020). Systematic Review of Important Viral Diseases in Africa in Light of the 'One Health' Concept. Pathog. 9 (4), 1–83. 10.3390/pathogens9040301 PMC7238228 32325980

[B86] Chiang C. Y. El Sony A. (2020). Tackling the threat of COVID-19 in Africa: an urgent need for practical planning. Int. J. Tuberculosis Lung Dis. 24 (5), 541–542. 10.5588/ijtld.20.0192 32398210

[B87] Chigome A. K. Matlala M. Godman B. Meyer J. C. (2019). Availability and Use of Therapeutic Interchange Policies in Managing Antimicrobial Shortages among South African Public Sector Hospitals; Findings and Implications. Antibiotics 9 (1), 1–11. 10.3390/antibiotics9010004 PMC7168338 31861923

[B88] Chilinda W. (2020). Government To Recruit 8,000 Health Work In 2020. Available at: https://www.znbc.co.zm/news/government-to-recruit-8000-health-work-in-2020/.

[B89] Chowdhury M. S. Rathod J. Gernsheimer J. (2020). A Rapid Systematic Review of Clinical Trials Utilizing Chloroquine and Hydroxychloroquine as a Treatment for COVID-19. Acad. Emergency Med. 27 (6), 493–504. 10.1111/acem.14005 PMC7267507 32359203

[B90] Clinical Trials Arena (2020). Genentech's arthritis drug tocilizumab shows promise in Covid-19 trial. Available at: https://www.clinicaltrialsarena.com/news/french-early-trial-tocilizumab-covid-19/.

[B91] Cohen I. G. Gostin L. O. Weitzner D. J. (2020). Digital Smartphone Tracking for COVID-19: Public Health and Civil Liberties in Tension. JAMA. 10.1001/jama.2020.8570 32459289

[B92] Conway M. Holt T. Sabow A. Sun I. Y. (2020). Should sub-Saharan Africa make its own drugs? Available at: https://www.mckinsey.com/industries/public-sector/our-insights/should-sub-saharan-africa-make-its-own-drugs

[B93] Cortegiani A. Ingoglia G. Ippolito M. Giarratano A. Einav S. (2020). A systematic review on the efficacy and safety of chloroquine for the treatment of COVID-19. J. Crit. Care 57, 279–283. 32173110 10.1016/j.jcrc.2020.03.005 PMC7270792

[B94] Coulibaly N. (2020). To fight coronavirus, Burkina Faso is tempted by chloroquine. Available at: https://www.theafricareport.com/25416/to-fight-coronavirus-burkina-faso-is-tempted-by-chloroquine/

[B95] Council for International Organizations of Medical Sciences (2020). Medicines assessment during public health emergencies needs good science, best practices and proper communication. Available at: https://cioms.ch/wp-content/uploads/2020/06/CIOMS_WGXII_Statement.pdf.

[B96] Courtemanche C. Garuccio J. Le A. Pinkston J. Yelowitz A. (2020). Strong Social Distancing Measures In The United States Reduced The COVID-19 Growth Rate. Health Affairs. 10.1377/hlthaff.2020.00608 32407171

[B97] Craven M. Mysore M. Singhal S. Wilson M. (2020). COVID-19: Briefing note, April 13, 2020 - Our latest perspectives on the coronavirus pandemic. Available at: https://www.mckinsey.com/business-functions/risk/our-insights/covid-19-implications-for-business

[B98] Cullen W. Gulati G. Kelly B. D. (2020). Mental health in the COVID-19 pandemic. QJM Monthly J. Assoc. Phys. 113 (5), 311–312. 10.1093/qjmed/hcaa110 PMC7184387 32227218

[B99] Cunningham A. C. Goh H. P. Koh D. (2020). Treatment of COVID-19: old tricks for new challenges. Crit. Care 24 (1), 91. 32178711 10.1186/s13054-020-2818-6 PMC7076992

[B100] da Silva W. C. de Araujo V. E. Lima E. Dos Santos J. B. R. Silva M. Almeida P. (2018). Comparative Effectiveness and Safety of Monoclonal Antibodies (Bevacizumab, Cetuximab, and Panitumumab) in Combination with Chemotherapy for Metastatic Colorectal Cancer: A Systematic Review and Meta-Analysis. BioDrugs 32 (6), 585–606. 30499082 10.1007/s40259-018-0322-1 PMC6290722

[B101] Dabanga (2020). OCHA Sudan: Medical supplies may be affected by Covid-19 measures. Available at: https://www.dabangasudan.org/en/relief-news/article/ocha-sudan-medical-supplies-may-be-affected-by-covid-19-measures.

[B102] Daily Monitor (2020). Uganda scientists develop devices to fight Covid-19. Available at: https://www.monitor.co.ug/News/National/Uganda-scientists-develop-devices-to-fight-Covid19/688334-5530170-11h0vedz/index.html.

[B103] Das S. Bhowmick S. Tiwari S. Sen S. (2020). An Updated Systematic Review of the Therapeutic Role of Hydroxychloroquine in Coronavirus Disease-19 (COVID-19). Clin. Drug Invest. 40 (7), 591–601. 10.1007/s40261-020-00927-1 PMC7255448 32468425

[B104] Davis R. (2020). Viral outbreak: Fake news spreads in SA in tandem with Covid-19. Available at: https://www.dailymaverick.co.za/article/2020-03-31-viral-outbreak-fake-news-spreads-in-sa-in-tandem-with-covid-19/.

[B105] Defy (2020). Doing Our Part. Available at: https://www.defy.co.za/doing-our-part/.

[B106] Di Caro B. (2020). COVID-19 in Africa: insights from our 30 April WHO media briefing. Available at: https://www.weforum.org/agenda/2020/04/covid-19-in-africa-insights-from-our-30-april-who-media-briefing/.

[B107] Di Lorenzo G. Di Trolio R. (2020). Coronavirus Disease (COVID-19) in Italy: Analysis of Risk Factors and Proposed Remedial Measures. Front. Med. 7 (140), 1–4. 10.3389/fmed.2020.00140 PMC7161343 32328496

[B108] Dietz W. Santos-Burgoa C. (2020). Obesity and its Implications for COVID-19 Mortality. Obesity 28 (6), 1005. 32237206 10.1002/oby.22818

[B109] Docherty A. B. Harrison E. M. Green C. A. Hardwick H. E. Pius R. Norman L. (2020). Features of 20 133 UK patients in hospital with covid-19 using the ISARIC WHO Clinical Characterisation Protocol: prospective observational cohort study. BMJ 369, m1985. 10.1136/bmj.m1985 32444460 PMC7243036

[B110] Donabedian A. (2005). Evaluating the quality of medical care. 1966. Milbank Q. 83 (4), 691–729. 16279964 10.1111/j.1468-0009.2005.00397.x PMC2690293

[B111] Du R. H. Liang L. R. Yang C. Q. Wang W. Cao T. Z. Li M. (2020). Predictors of Mortality for Patients with COVID-19 Pneumonia Caused by SARS-CoV-2: A Prospective Cohort Study. Eur. Respir. J. 55 (5), 2000524 32269088 10.1183/13993003.00524-2020 PMC7144257

[B112] Ducomble T. Gignoux E. (2020). Learning from a massive epidemic: measles in DRC. Lancet Infect. Dis. 20 (5), 542. 32243817 10.1016/S1473-3099(20)30265-6

[B113] Dugmore H. (2020). Covid-19 leaves SA at the mercy of overseas drug exporters. Available at: https://mg.co.za/article/2020-03-25-covid-19-leaves-sa-at-the-mercy-of-overseas-drug-exporters/.

[B114] Dyer O. (2020). Covid-19: Africa records over 10 000 cases as lockdowns take hold. BMJ (Clin. Res. Ed.) 369, m1439. 10.1136/bmj.m1439 32269023

[B115] EAC Secretariat (2020). East African Community COVID-19 Response Plan. Available at: https://www.eac.int/coronavirus.

[B116] EAC (2020). EAC Partner States directed to support local production of essential medical products and supplies to combat COVID-19 in the region. Available at: https://www.eac.int/press-releases/147-health/1724-eac-partner-state-directed-to-support-local-prodcution-of-essential-medical-products-and-suupliees-to-combat-covid-19-in-the-region.

[B117] East African (2020). Uganda endorses anti-malaria drug under clinical trial to cure Covid-19. Available at: https://www.monitor.co.ug/News/National/Uganda-endorses-anti-malaria-drug-clinical-trial-cure-Covid-19/688334-5531292-rob53m/index.html.

[B118] Ebrahim E. Lakay B. (2020). Mkhize: Health sector must manage chronic patients amid Covid-19 - or it may bring a new challenge. Available at: https://www.health24.com/Medical/Infectious-diseases/Coronavirus/mkhize-health-sector-must-manage-chronic-patients-amid-covid-19-or-it-may-bring-a-new-challenge-20200430.

[B119] ECDC (2020a). Vaccines and treatment of COVID-19. Available at: https://www.ecdc.europa.eu/en/covid-19/latest-evidence/vaccines-and-treatment.

[B120] ECDC (2020b). Ebola outbreak in the Democratic Republic of the Congo. Available at: https://www.ecdc.europa.eu/en/ebola-virus-disease-outbreak-democratic-republic-congo-ongoing.

[B121] Editor Food Business Africa (2020). Trade Kings Foundation launches hygiene drive against Covid-19 in Lusaka. Available at: https://www.foodbusinessafrica.com/2020/04/22/trade-kings-foundation-launches-hygiene-drive-against-covid-19-in-lusaka/.

[B122] Ekor M. (2014). The growing use of herbal medicines: issues relating to adverse reactions and challenges in monitoring safety. Front. Pharmacol. 4 (177), 1–10. 10.3389/fphar.2013.00177 PMC3887317 24454289

[B123] Ellinghaus D. Degenhardt F. Bujanda L. Buti M. Albillos A. Invernizzi P. (2020). Genomewide Association Study of Severe Covid-19 with Respiratory Failure. N Engl. J. Med. 10.1056/NEJMoa2020283 PMC7315890 32558485

[B124] El-Sabaa R. (2020). Egypt's health ministry launches coronavirus mobile application. Available at: http://english.ahram.org.eg/NewsContent/1/64/367263/Egypt/Politics-/Egypts-health-ministry-launches-coronavirus-mobile.aspx.

[B125] El-Sadr W. M. Justman J. (2020). Africa in the Path of Covid-19. N Engl. J. Med. 383 (3), e11. 32302075 10.1056/NEJMp2008193

[B126] EMA (2020). First COVID-19 treatment recommended for EU authorisation. 25 June 2020. Available at: https://www.ema.europa.eu/en/news/first-covid-19-treatment-recommended-eu-authorisation.

[B127] Endomba F. T. Wouna D. L. A. Danwang C. (2020). Mental health during the coronavirus disease 2019 (Covid-19) pandemic: more is still to be done. PAMJ 35 (2), 7. 10.11604/pamj.supp.2020.35.2.22605 PMC7266472 32528618

[B128] Ermisch M. Bucsics A. Vella Bonanno P. Arickx F. Bybau A. Bochenek T. (2016). Payers' Views of the Changes Arising through the Possible Adoption of Adaptive Pathways. Front. Pharmacol. 7 (305), 1–9. 27733828 10.3389/fphar.2016.00305 PMC5039228

[B129] European Medicine Agency (2020a). COVID-19: reminder of risk of serious side effects with chloroquine and hydroxychloroquine. Available at: https://www.ema.europa.eu/en/news/covid-19-reminder-risk-serious-side-effects-chloroquine-hydroxychloroquine.

[B130] European Medicines Agency (2020b). EU authorities agree new measures to support availability of medicines used in the COVID-19 pandemic. Available at: https://www.ema.europa.eu/en/news/eu-authorities-agree-new-measures-support-availability-medicines-used-covid-19-pandemic.

[B131] European Observatory (2016). How can voluntary cross-border collaboration in public procurement improve access to health technologies in Europe? Available at: http://www.euro.who.int/:data/assets/pdf_file/0009/331992/PB21.pdf?ua=1. 29144633

[B132] EY Tax News Update (2020). Zambia issues additional fiscal measures to mitigate the impact of COVID-19. Available at: https://taxnews.ey.com/news/2020-1113-zambia-issues-additional-fiscal-measures-to-mitigate-the-impact-of-covid-19?uAlertID=Sd%2FG8rua1oj6%2Fl58EZ2AiA%3D%3D.

[B133] Fang L. Karakiulakis G. Roth M. (2020). Are patients with hypertension and diabetes mellitus at increased risk for COVID-19 infection? Lancet Respir. Med. 8 (4), e21. 32171062 10.1016/S2213-2600(20)30116-8 PMC7118626

[B134] Fasseeh A. Karam R. Jameleddine M. George M. Kristensen F. B. Al-Rabayah A. A. (2020). Implementation of Health Technology Assessment in the Middle East and North Africa: Comparison Between the Current and Preferred Status. Front. Pharmacol. 11 (15), 1–9. 32153393 10.3389/fphar.2020.00015 PMC7046555

[B135] Ferner R. E. Aronson J. K. (2020). Chloroquine and hydroxychloroquine in covid-19. BMJ 369, m1432. 32269046 10.1136/bmj.m1432

[B136] Ferrario A. Arāja D. Bochenek T. Čatić T. Dankó D. Dimitrova M. (2017). The Implementation of Managed Entry Agreements in Central and Eastern Europe: Findings and Implications. PharmacoEconomics 35 (12), 1271–1285. 28836222 10.1007/s40273-017-0559-4 PMC5684278

[B137] Ford N. Vitoria M. Rangaraj A. Norris S. L. Calmy A. Doherty M. (2020). Systematic review of the efficacy and safety of antiretroviral drugs against SARS, MERS or COVID-19: initial assessment. J. Int. AIDS Soc. 23 (4), e25489. 32293807 10.1002/jia2.25489 PMC7158851

[B138] Forrest A. (2020). Coronavirus: 700 dead in Iran after drinking toxic methanol alcohol to cure Covid-19. Available at: https://www.msn.com/en-gb/news/coronavirus/coronavirus-700-dead-in-iran-after-drinking-toxic-methanol-alcohol-to-cure-covid-19/ar-BB13jC6f?ocid=spartandhp.

[B139] Founou R. C. Founou L. L. Essack S. Y. (2017). Clinical and economic impact of antibiotic resistance in developing countries: A systematic review and meta-analysis. PLoS One 12 (12), 1–18. 10.1371/journal.pone.0189621 PMC5739407 29267306

[B140] Gaborit B. J. Bergmann J. F. Mussini C. Arribas J. R. Behrens G. Walmsley S. (2020). Plea for multitargeted interventions for severe COVID-19. Lancet Infect. Dis. 10.1016/S1473-3099(20)30312-1 PMC7172613 32325035

[B141] Gallagher F. (2020). Tracking hydroxychloroquine misinformation: How an unproven COVID-19 treatment ended up being endorsed by Trump. ABC News. Available at: https://abcnews.go.com/Health/tracking-hydroxychloroquine-misinformation-unproven-covid-19-treatment-ended/story?id=70074235.

[B142] Gao J. Tian Z. Yang X. (2020). Breakthrough: Chloroquine phosphate has shown apparent efficacy in treatment of COVID-19 associated pneumonia in clinical studies. Biosci. Trends 14 (1), 72–73. 32074550 10.5582/bst.2020.01047

[B143] Gautret P. Lagier J. C. Parola P. Hoang V. T. Meddeb L. Mailhe M. (2020). Hydroxychloroquine and azithromycin as a treatment of COVID-19: results of an open-label non-randomized clinical trial. Int. J. Antimicrob. Agents, 105949. 32205204 10.1016/j.ijantimicag.2020.105949 PMC7102549

[B144] Geldsetzer P. (2020). Use of Rapid Online Surveys to Assess People's Perceptions During Infectious Disease Outbreaks: A Cross-sectional Survey on COVID-19. J. Med. Internet Res. 22 (4), e18790–e1879e. 32240094 10.2196/18790 PMC7124956

[B145] Geleris J. Sun Y. Platt J. Zucker J. Baldwin M. Hripcsak G. (2020). Observational Study of Hydroxychloroquine in Hospitalized Patients with Covid-19. N Engl. J. Med. 382 (25), 2411–2418. 32379955 10.1056/NEJMoa2012410 PMC7224609

[B146] Ghana Ministry of Health (2018). Ministry of Food and Agriculture, Ministry of Environment, Science, Technology and Innovation, Ministry of Fisheries and Aquaculture Development. Ghana National Action Plan for Antimicrobial Use and Resistance. 2017 - 2021. Available at: http://www.moh.gov.gh/wp-content/uploads/2018/04/NAP_FINAL_PDF_A4_19.03.2018-SIGNED-1.pdf.

[B147] Ghana News Agency (2020). Successful testing regime and controls inform lifting of lockdown. Issued April 20 2020. Available at: https://newsghana.com.gh/successful-testing-regime-and-controls-inform-lifting-of-lockdown/.

[B148] Ghana Prorities Project (2020). A rapid cost-benefit analysis of moderate social distancing in response to the COVID-19 pandemic in Ghana. 20 May 2020. Available at: https://www.copenhagenconsensus.com/sites/default/files/covid_brief_for_ndpc_ghana_final.pdf.

[B149] GhanaWeb (2020a). Pharmaceutical companies to produce 70% of country's drug needs. Available at: https://www.ghanaweb.com/GhanaHomePage/NewsArchive/Pharmaceutical-companies-to-produce-70-of-country-s-drug-needs-936016.

[B150] GhanaWeb (2020b). Coronavirus: Stop discriminating against recovered patients. Available at: https://www.ghanaweb.com/GhanaHomePage/NewsArchive/Coronavirus-Stop-discriminating-against-recovered-patients-Fred-Drah-926353.

[B151] Ghosal A. Milko V. Coronavirus could erode global fight against other diseases. Available at: https://www.msn.com/en-gb/news/world/coronavirus-could-erode-global-fight-against-other-diseases/ar-BB12HxuG?ocid=spartandhp#image=1.

[B152] Global Coronavirus COVID-19 Clinical Trial Tracker. (2020). Available at: https://www.covid-trials.org/.

[B153] Glied S. Levy H. (2020). The Potential Effects of Coronavirus on National Health Expenditures. JAMA. 10.1001/jama.2020.6644 32338730

[B154] Godman B. Wettermark B. van Woerkom M. Fraeyman J. Alvarez-Madrazo S. Berg C. (2014). Multiple policies to enhance prescribing efficiency for established medicines in Europe with a particular focus on demand-side measures: findings and future implications. Front. Pharmacol. 5, 106. 24987370 10.3389/fphar.2014.00106 PMC4060455

[B155] Godman B. Malmstrom R. E. Diogene E. Jayathissa S. McTaggart S. Cars T. (2014b). Dabigatran - a continuing exemplar case history demonstrating the need for comprehensive models to optimize the utilization of new drugs. Front. Pharmacol. 5, 109. 24959145 10.3389/fphar.2014.00109 PMC4050532

[B156] Godman B. Malmstrom R. E. Diogene E. Gray A. Jayathissa S. Timoney A. (2015). Are new models needed to optimize the utilization of new medicines to sustain healthcare systems? Expert Rev. Clin. Pharmacol. 8 (1), 77–94. 25487078 10.1586/17512433.2015.990380

[B157] Godman B. Bucsics A. Vella Bonanno P. Oortwijn W. Rothe C. C. Ferrario A. (2018). Barriers for Access to New Medicines: Searching for the Balance Between Rising Costs and Limited Budgets. Front. Public Health 6, 328. 30568938 10.3389/fpubh.2018.00328 PMC6290038

[B158] Godman B. Grobler C. Van-De-Lisle M. Wale J. Barbosa W. B. Massele A. (2019). Pharmacotherapeutic interventions for bipolar disorder type II: addressing multiple symptoms and approaches with a particular emphasis on strategies in lower and middle-income countries. Expert Opin. Pharmacother. 20 (18), 2237–2255. 31762343 10.1080/14656566.2019.1684473

[B159] Godman B. (2020). Combating COVID-19: Lessons learnt particularly among developing countries and the implications. Bangladesh J. Med. Sci. S103–8. 10.3329/bjms.v19i0.48413

[B160] Godman B. Haque M. McKimm J. Abu Bakar M. Sneddon J. Wale J. (2020a). Ongoing strategies to improve the management of upper respiratory tract infections and reduce inappropriate antibiotic use particularly among lower and middle-income countries: findings and implications for the future. Curr. Med. Res. Opin. 36 (2), 301–327. 31794332 10.1080/03007995.2019.1700947

[B161] Godman B. Basu D. Pillay Y. Almeida P. Mwita J. C. Rwegerera G. M. (2020b). Ongoing and planned activities to improve the management of patients with Type 1 diabetes across Africa; implications for the future. Hosp. Pract. 48 (2), 51–67. 10.1080/21548331.2020.1745509 32196395

[B162] Godman B. Basu D. Pillay Y. Mwita J. C. Rwegerera G. M. Anand Paramadhas B. D. (2020c). Review of Ongoing Activities and Challenges to Improve the Care of Patients With Type 2 Diabetes Across Africa and the Implications for the Future. Front. Pharmacol. 11 (108), 1–21. 32265688 10.3389/fphar.2020.00108 PMC7098994

[B163] Godman B. McCabe H. Leong T. D. (2020d). Fixed dose drug combinations - are they pharmacoeconomically sound? Findings and implications especially for lower- and middle-income countries. Expert Rev. Pharmacoecon. Outc. Res. 20 (1), 1–26. 10.1080/14737167.2020.1734456 32237953

[B164] González-Sanguino C. Ausín B. Castellanos M. Saiz J. López-Gómez A. Ugidos C. (2020). Mental health consequences during the initial stage of the 2020 Coronavirus pandemic (COVID-19) in Spain. Brain Behav. Immun. 10.1016/j.bbi.2020.05.040 PMC7219372 32405150

[B165] Goodman J. L. Borio L. (2020). Finding Effective Treatments for COVID-19: Scientific Integrity and Public Confidence in a Time of Crisis. JAMA 87, 172–176. 10.1001/jama.2020.6434 32297900

[B166] Gounden P. Barden C. Charter C. (2020). Taking stock: Competition law's response to the novel COVID-19 outbreak. Available at: https://www.cliffedekkerhofmeyr.com/en/news/publications/2020/Competition/competition-alert-1-april-Taking-stock-Competition-laws-response-to-the-novel-COVID-19-outbreak.html.

[B167] Government of The Kingdom of Eswatini (2020). Prime Minister's Statement - Partial Lockdown Strengthened Measures. Available at: http://www.gov.sz/images/CORONA/PM-statement-22-April-2020.pdf.

[B168] Gower P. (2020). UP produces interactive app with real-time data on COVID-19 infections. Available at: https://www.up.ac.za/news/post_2885914-up-produces-interactive-app-with-real-time-data-on-covid-19-infections.

[B169] Guan W.-J. Ni Z.-Y. Hu Y. Liang W.-H. Ou C.-Q. He J.-X. (2020). Clinical Characteristics of Coronavirus Disease 2019 in China. New Engl. J. Med. 382 (18), 1708–1720. 32109013 10.1056/NEJMoa2002032 PMC7092819

[B170] Guo W. Ming F. Dong Y. Zhang Q. Zhang X. Mo P. (2020). A Survey for COVID-19 Among HIV/AIDS Patients in Two Districts of Wuhan, China. Lancet.

[B171] GuruGamer (2020). Warning: An American Died Of Using A Chloroquine Overdose Suggested As A Coronavirus Cure By President Trump. Available at: https://gurugamer.com/features/an-american-died-of-using-a-chloroquine-overdose-suggested-as-a-coronavirus-cure-by-president-trump-9466.

[B172] Habersaat K. B. Betsch C. Danchin M. Sunstein C. R. Böhm R. Falk A. (2020). Ten considerations for effectively managing the COVID-19 transition. Nat. Hum. Behav. 4 (7), 677–687. 32581299 10.1038/s41562-020-0906-x

[B173] Haigh L. (2020). Malaria in Zimbabwe exacerbated by Covid-19. Available at: https://www.itij.com/latest/news/malaria-zimbabwe-exacerbated-covid-19.

[B174] Hall C. (2020). This One Thing Could Be the Key to Reducing the Progression of COVID-19. Available at: https://www.msn.com/en-us/health/medical/this-one-thing-could-be-the-key-to-reducing-the-progression-of-covid-19/ar-BB12Wb84.

[B175] Hamid S. Mir M. Y. Rohela G. K. (2020). Novel coronavirus disease (COVID-19): a pandemic (epidemiology, pathogenesis and potential therapeutics). New Microbes New Infect. 35, 100679. 32322401 10.1016/j.nmni.2020.100679 PMC7171518

[B176] Haque M. Islam S. Iqbal S. Urmi U. L. Kamal Z. M. Shuvo S. A. (2020). Availability and price changes of potential medicines and equipment for the prevention and treatment of COVID-19 among pharmacy and drug stores in Bangladesh; findings and implications. Bangladesh J. Med. Sci. 19 (Special Issue on Covid19), S36–S50.

[B177] Härter G. Spinner C. D. Roider J. Bickel M. Krznaric I. Grunwald S. (2020). COVID-19 in people living with human immunodeficiency virus: a case series of 33 patients. Infection, 1–6. 32394344 10.1007/s15010-020-01438-z PMC7211976

[B178] Hatswell A. J. (2020). Learnings for Health Economics from the Early Stages of the COVID-19 Pandemic. PharmacoEconom. - Open 4 (2), 203–205. 10.1007/s41669-020-00216-9 PMC7147136 32277366

[B179] Hayat K. Rosenthal M. Xu S. Arshed M. Li P. Zhai P. (2020). View of Pakistani Residents toward Coronavirus Disease (COVID-19) during a Rapid Outbreak: A Rapid Online Survey. Int. J. Environ. Res. Public Health 17 (10), 1–10. 10.3390/ijerph17103347 PMC7277197 32408528

[B180] He J. He L. Zhou W. Nie X. He M. (2020). Discrimination and Social Exclusion in the Outbreak of COVID-19. Int. J. Environ. Res. Public Health 17 (8), 1–4. 10.3390/ijerph17082933 PMC7215298 32340349

[B181] Health 24 (2020). Dramatic drop in SA's immunisation rates. 26 June 2020. Available at: https://www.health24.com/Medical/Childhood-diseases/Vaccinations/dramatic-drop-in-sas-immunisation-rates-20200624-4.

[B182] Hedima E. W. Adeyemi M. S. Ikunaiye N. Y. (2020). Community Pharmacists: On the frontline of health service against COVID-19 in LMICs. Res. Soc. Adm. Pharm. 10.1016/j.sapharm.2020.04.017 PMC7167226 32340893

[B183] Hernandez-Villafuerte K. Li R. Hofman K. J. (2016). Bibliometric trends of health economic evaluation in Sub-Saharan Africa. Global Health 12 (1), 50. 27558556 10.1186/s12992-016-0188-2 PMC4997754

[B184] High Commission of the Republic of Zambia (2020). Statement by the Hon. Minister of finance on covid-19. Available at: https://www.zambiahc.org.uk/news_events/statement-by-the-hon-minister-of-finance-on-covid-19/.

[B185] Ho U. (2020). Covid-19 precaution: Some patients on chronic meds to have prescriptions filled for up to four months. Sportlight Health Journalism. 31 March 2020. Available from URL: http://www.msn.com/en-za/health/healthnews/covid-19-precaution-some-patients-on-chronic-meds-to-have-prescriptions-filled-for-up-to-four-months/ar-BB11VVGr

[B186] Hofer U. (2019). The cost of antimicrobial resistance. Nat. Rev. Microbiol. 17 (1), 3. 30467331 10.1038/s41579-018-0125-x

[B187] Hofman K. Goldstein S. (2020). COVID-19 risks forcing SA to make health trade-offs it can ill afford. Available at: https://www.wits.ac.za/covid19/covid19-news/latest/covid-19-risks-forcing-sa-to-make-health-trade-offs-it-can-ill-afford.html.

[B188] Hollingworth S. Odame E. Winch A. (2018). Understanding data needs for HTA in Sub-Saharan Africa – a framework and Ghana case study. Available at: https://www.idsihealth.org/wp-content/uploads/2018/10/Data-Sources-.pdf.

[B189] Houreld K. Lewis D. (2020). In Africa, lack of coronavirus data raises fears of silent epidemic. 8 July 2020. Available at: https://www.yahoo.com/news/africa-lack-coronavirus-data-raises-081603106.html

[B190] Houreld K. Lewis D. McNeill R. Granado S. (2020). Virus exposes gaping holes in Africa's health systems. Available at: https://graphics.reuters.com/HEALTH-CORONAVIRUS/AFRICA/yzdpxoqbdvx/?fbclid=IwAR2Xu9J5CLPuUfxWyzFzi-uiSlixjgxsJDPXh0Loa40HNnEnisi0glXCG_A.

[B191] Huang C. Wang Y. Li X. Ren L. Zhao J. Hu Y. (2020). Clinical features of patients infected with 2019 novel coronavirus in Wuhan, China. Lancet 395 (10223), 497–506. 31986264 10.1016/S0140-6736(20)30183-5 PMC7159299

[B192] Huang I. Lim M. A. Pranata R. (2020). Diabetes mellitus is associated with increased mortality and severity of disease in COVID-19 pneumonia - A systematic review, meta-analysis, and meta-regression. Diabetes Metab. Syndrome 14 (4), 395–403. 10.1016/j.dsx.2020.04.018 PMC7162793 32334395

[B193] Hung I. F.-N. Lung K.-C. Tso E. Y.-K. Liu R. Chung T. W.-H. Chu M.-Y. (2020). Triple combination of interferon beta-1b, lopinavir–ritonavir, and ribavirin in the treatment of patients admitted to hospital with COVID-19: an open-label, randomised, phase 2 trial. Lancet 395 (10238), 1695–1704. 32401715 10.1016/S0140-6736(20)31042-4 PMC7211500

[B194] Hwang T. J. Rabheru K. Peisah C. Reichman W. Ikeda M. (2020). Loneliness and Social Isolation during the COVID-19 Pandemic. Int. Psychogeriatrics 1–15. 10.1017/S1041610220000988 PMC7306546 32450943

[B195] IFRC, UNICEF and WHO (2020). Social Stigma associated with COVID-19. Available at: https://reliefweb.int/sites/reliefweb.int/files/resources/covid19-stigma-guide.pdf.

[B196] Ighobor K. (2020). Together we can win the war against COVID-19. Available at: https://www.un.org/africarenewal/magazine/special-edition-covid-19/together-we-can-win-war-against-covid-19.

[B197] IHME (2020a). New COVID-19 forecasts for Europe: Italy & Spain have passed the peak of their epidemics; UK, early in its epidemic, faces a fast-mounting death toll. Available at: http://www.healthdata.org/news-release/new-covid-19-forecasts-europe-italy-spain-have-passed-peak-their-epidemics-uk-early-its.

[B198] IHME (2020b). New COVID-19 forecasts: US hospitals could be overwhelmed in the second week of April by demand for ICU beds, and US deaths could total 81,000 by July. Available at: http://www.healthdata.org/news-release/new-covid-19-forecasts-us-hospitals-could-be-overwhelmed-second-week-april-demand-icu.

[B199] IMF (2020). IMF Executive Board Approves a US$491.5 Million Disbursement to Uganda to Address the COVID-19 Pandemic. Available at: https://www.imf.org/en/News/Articles/2020/05/06/pr20206-uganda-imf-executive-board-approves-us-million-disbursement-address-the-covid-19-pandemic.

[B200] Inciardi R. M. Lupi L. Zaccone G. Italia L. Raffo M. Tomasoni D. (2020). Cardiac Involvement in a Patient With Coronavirus Disease 2019 (COVID-19). JAMA Cardiol. 5 (7), 1–6. 10.1001/jamacardio.2020.1096 PMC7364333 32219357

[B201] International Alliance of Patients' Organizations (2020). COVID-19 Resources Hub. Available at: https://www.iapo.org.uk/covid-19-resources-hub.

[B202] International Society of Antimicrobial Chemotherapy (2020). Official Statement from International Society of Antimicrobial Chemotherapy (ISAC) - Hydroxychloroquine and azithromycin as a treatment of COVID-19: results of an open-label non-randomized clinical trial (Gautret P et al. PMID 32205204). Available at: https://www.isac.world/news-and-publications/official-isac-statement. 10.1016/j.ijantimicag.2020.105949 PMC7102549 32205204

[B203] IOL (2020). In a Covid-19 world, telemedicine will be the new normal. Available at: https://www.iol.co.za/lifestyle/health/in-a-covid-19-world-telemedicine-will-be-the-new-normal-47357559.

[B204] ISAC/ Elsevier (2020). Joint ISAC and Elsevier statement on Gautret et al. paper [PMID 32205204]. Available at: https://www.isac.world/news-and-publications/isac-elsevier-statement.

[B205] Jayaram K. Leke A. Ooko-Ombaka A. Sun Y. S. (2020). Tackling COVID-19 in Africa - An unfolding health and economic crisis that demands bold action. Available at: https://www.mckinsey.com/featured-insights/middle-east-and-africa/tackling-covid-19-in-africa#.

[B206] Jerving S. (2020). How COVID-19 could complicate treatment for HIV patients. Available at: https://www.devex.com/news/how-covid-19-could-complicate-treatment-for-hiv-patients-96884.

[B207] JKUAT (2020a). JKUAT Trio Develop Apps to Trace, Triage and Manage COVID-19. Available at: http://www.jkuat.ac.ke/jkuat-trio-develop-contact-tracing-and-case-management-apps/.

[B208] JKAUT (2020b). JKUAT's Team of Engineers Invent Ventilators to Fill Shortage. Available at: http://www.jkuat.ac.ke/jkuats-team-of-engineers-invent-ventilators-to-fill-shortage/.

[B209] Jones A. (2020). Coronavirus: How 'overreaction' made Vietnam a virus success. Available at: https://www.bbc.co.uk/news/world-asia-52628283.

[B210] Jose R. J. Manuel A. (2020). COVID-19 cytokine storm: the interplay between inflammation and coagulation. Lancet Respir. Med. 8 (6), e46–e47. 32353251 10.1016/S2213-2600(20)30216-2 PMC7185942

[B211] Journal du Cameroun (2020). Cameroon: Monkey pox hits East region, kills one. Available at: https://www.journalducameroun.com/en/cameroon-monkey-pox-hits-east-region-kills-one/.

[B212] Kabale N. Otsialo M. Makong B. Njeru A. Muchui D. Chepngeno E. (2020). Concern as patients shun health centres. Available at: https://www.nation.co.ke/news/Concern-as-patients-shun-health-centres/1056-5543778-13ejdvwz/index.html.

[B213] Kaine E. Nwokik J. (2020). Scaling Up African Pharmaceutical Manufacturing in a Time of COVID-19. Available at: https://www.cfr.org/blog/scaling-african-pharmaceutical-manufacturing-time-covid-19.

[B214] Kalungia A. Godman B. (2019). Implications of non-prescription antibiotic sales in China. Lancet Infect. Dis. 19 (12), 1272–1273. 31588041 10.1016/S1473-3099(19)30408-6

[B215] Kalungia A. C. Burger J. Godman B. Costa J. O. Simuwelu C. (2016). Non-prescription sale and dispensing of antibiotics in community pharmacies in Zambia. Expert Rev. Anti-Infe. Ther. 14 (12), 1215–1223. 10.1080/14787210.2016.1227702 27548801

[B216] Kansiime S. Mwesigire D. Mugerwa H. (2019). Prevalence of non-communicable diseases among HIV positive patients on antiretroviral therapy at joint clinical research centre, Lubowa, Uganda. PLoS One 14 (8), e0221022. 31398244 10.1371/journal.pone.0221022 PMC6688817

[B217] Kasozi K. I. Mujinya R. Bogere P. Ekou J. Zirintunda G. Ahimbisibwe S. (2020). Pandemic panic and anxiety in developing countries. Embracing One Health offers practical strategies in management of COVID-19 for Africa. PAMJ 35 (2):3. 10.11604/pamj.supp.2020.35.2.22637 PMC7266473 32528614

[B218] Kaufman K. R. Petkova E. Bhui K. S. Schulze T. G. (2020). A global needs assessment in times of a global crisis: world psychiatry response to the COVID-19 pandemic. BJPsych. Open 6 (3), e48. 32250235 10.1192/bjo.2020.25 PMC7211994

[B219] Kavanagh M. M. Erondu N. A. Tomori O. Dzau V. J. Okiro E. A. Maleche A. (2020). Access to lifesaving medical resources for African countries: COVID-19 testing and response, ethics, and politics. Lancet 395 (10238), 1735–1738. 32386564 10.1016/S0140-6736(20)31093-X PMC7252104

[B220] KCE Report (2017). KCE Report number 283. Horizon Scanning For Pharmaceuticals: Proposal For The Beneluxa Collaboration. Available at: http://www.beneluxa.org/sites/beneluxa.org/files/2017-07/Horizon%20scanning_ScientificReport_full.pdf.

[B221] Keates A. K. Mocumbi A. O. Ntsekhe M. Sliwa K. Stewart S. (2017). Cardiovascular disease in Africa: epidemiological profile and challenges. Nat. Rev. Cardiol. 14 (5), 273–293. 28230175 10.1038/nrcardio.2017.19

[B222] Kenna H. A. Poon A. W. de los Angeles C. P. Koran L. M. (2011). Psychiatric complications of treatment with corticosteroids: review with case report. Psychiatry Clin. Neurosci. 65 (6), 549–560. 22003987 10.1111/j.1440-1819.2011.02260.x

[B223] Kenyatta University (2020). K.U Makes Ventilators. Available at: http://www.ku.ac.ke/component/k2/item/1655-k-u-makes-ventilators.

[B224] Khan M. S. Durrance-Bagale A. Legido-Quigley H. Mateus A. Hasan R. Spencer J. (2019). ‘LMICs as reservoirs of AMR': a comparative analysis of policy discourse on antimicrobial resistance with reference to Pakistan. Health Policy Plan 34 (3), 178–187. 30977804 10.1093/heapol/czz022

[B225] Khan M. S. Bory S. Rego S. Suy S. Durrance-Bagale A. Sultana Z. (2020). Is enhancing the professionalism of healthcare providers critical to tackling antimicrobial resistance in low- and middle-income countries? Hum. Resour. Health 18 (1), 10. 32046723 10.1186/s12960-020-0452-7 PMC7014603

[B226] Khunti K. Singh A. K. Pareek M. Hanif W. (2020). Is ethnicity linked to incidence or outcomes of covid-19? BMJ 369, m1548. 32312785 10.1136/bmj.m1548

[B227] Kindzeka M. E. (2019a). More Deaths Feared From Cholera in Cameroon. Available at: https://www.voanews.com/africa/more-deaths-feared-cholera-cameroon.

[B228] Kindzeka M. E. (2019b). Cameroon Launches Vaccination Campaign to Contain Measles Outbreak. Available at: https://www.voanews.com/africa/cameroon-launches-vaccination-campaign-contain-measles-outbreak.

[B229] Kindzeka M. K. (2020). Cameroon Seizes Fake Coronavirus Drugs Sold by Scammers. Available at: https://www.voanews.com/science-health/coronavirus-outbreak/cameroon-seizes-fake-coronavirus-drugs-sold-scammers.

[B230] Kirby T. (2020). Evidence mounts on the disproportionate effect of COVID-19 on ethnic minorities. Lancet Respir. Med. 8 (6), 547–548. 32401711 10.1016/S2213-2600(20)30228-9 PMC7211498

[B231] Kluge H. H. P. Wickramasinghe K. Rippin H. L. Mendes R. Peters D. H. Kontsevaya A. (2020). Prevention and control of non-communicable diseases in the COVID-19 response. Lancet 395 (10238), 1678–1680. 32401713 10.1016/S0140-6736(20)31067-9 PMC7211494

[B232] Knott S. (2020). Covid-19 could mark a deadly turn in Ghana's fight against fake drugs. Available at: https://www.theguardian.com/global-development/2020/apr/30/covid-19-could-mark-a-deadly-turn-in-ghana-fight-against-fake-drugs.

[B233] Kowalska J. D. Skrzat-Klapaczyńska A. Bursa D. Balayan T. Begovac J. Chkhartishvili N. (2020). HIV care in times of the COVID-19 crisis - where are we now in Central and Eastern Europe? Int. J. Infect. Dis. 96, 311–314. 32413608 10.1016/j.ijid.2020.05.013 PMC7211569

[B234] Kozloff N. Mulsant B. H. Stergiopoulos V. Voineskos A. N. (2020). The COVID-19 Global Pandemic: Implications for People With Schizophrenia and Related Disorders. Schizophr. Bull. 46 (4), 752–757. 32343342 10.1093/schbul/sbaa051 PMC7197583

[B235] Kretchy I. A. Asiedu-Danso M. Kretchy J. P. (2020). Medication management and adherence during the COVID-19 pandemic: Perspectives and experiences from low-and middle-income countries. Res. Soc. Adm. Pharm. 10.1016/j.sapharm.2020.04.007 PMC7158799 32307319

[B236] Krubiner C. Keller J. M. Kaufman J. (2020). Balancing the COVID-19 Response with Wider Health Needs: Key Decision-Making Considerations for Low- and Middle-Income Countries. Available at: https://www.cgdev.org/sites/default/files/balancing-covid-19-response-wider-health-needs-key-decision-making-considerations-low.pdf.

[B237] Kumar S. U. Kumar D. T. Christopher B. P. Doss C. G. P. (2020). The Rise and Impact of COVID-19 in India. Front. Med. 7, 250. 10.3389/fmed.2020.00250 PMC7256162 32574338

[B238] Kumwenda T. (2020). CFTC Cautions 11 Pharmacies On Over-charging Items. Available at: https://www.zodiakmalawi.com/nw/national-news/64-news-in-northern-region/1528-cftc-cautions-11-pharmacies-on-over-charging-items.

[B239] Kyeyune H. (2020). Shutdown in Uganda over COVID-19 hits poor hard - Times tough not only due to fears of infection but also because of poverty, says shop owner. Available at: https://www.aa.com.tr/en/africa/shutdown-in-uganda-over-covid-19-hits-poor-hard/1787526.

[B240] Lacobucci G. (2020). NICE issues covid-19 critical care guideline. BMJ 368, m1177.32217519

[B241] Lakay B. (2020). Your 6-month prescription expiring? Your pharmacist may extend it, new regulations state. Available at: https://www.health24.com/Medical/Infectious-diseases/Coronavirus/your-6-month-prescription-expiring-your-pharmacist-may-extend-it-new-regulations-state-20200430-2.

[B242] LaMarca S. (2020). Scientists In Senegal Developed A $1 COVID-19 Testing Kit And Plan To Export Millions To African Countries. Available at: https://tanksgoodnews.com/2020/04/29/senegal-1-testing-kit/.

[B243] Lancet editorial (2020). COVID-19 in Africa: no room for complacency. Lancet 395, 1669. 32473665 10.1016/S0140-6736(20)31237-X PMC7255759

[B244] Lapolla P. Mingoli A. Lee R. (2020). Deaths from COVID-19 in healthcare workers in Italy - what can we learn? Infection Control Hosp. Epidemiol. 1–4. 10.1017/ice.2020.241 PMC7256220 32408922

[B245] Larson H. (2020). Blocking information on COVID-19 can fuel the spread of misinformation - Governments need to think twice before they suppress messages related to COVID-19. Nature 580, 306. 32231320 10.1038/d41586-020-00920-w

[B246] Le Roux C. Dramowski A. (2020). Personal protective equipment (PPE) in a pandemic: Approaches to PPE preservation for South African healthcare facilities. S. Afr. Med. J. 110 (6), 466–468. 32880554 10.7196/SAMJ.2020.v110i6.14831

[B247] Li Q. Guan X. Wu P. Wang X. Zhou L. Tong Y. (2020). Early Transmission Dynamics in Wuhan, China, of Novel Coronavirus-Infected Pneumonia. N Engl. J. Med. 382 (13), 1199–1207. 31995857 10.1056/NEJMoa2001316 PMC7121484

[B248] Li W. Yang Y. Liu Z. H. Zhao Y. J. Zhang Q. Zhang L. (2020). Progression of Mental Health Services during the COVID-19 Outbreak in China. Int. J. Biol. Sci. 16 (10), 1732–1738. 32226291 10.7150/ijbs.45120 PMC7098037

[B249] LinksCommunity (2020). Leveraging Technology to Improve Health Care During the COVID-19 Pandemic and Beyond. Available at: https://linkscommunity.org/assets/PDFs/cov039_telemedicine_v3_14may2020.pdf.

[B250] Littlejohn E. (2020). Hydroxychloroquine use in the COVID-19 patient. Cleveland Clin. J. Med. 10.3949/ccjm.87a.ccc011 32371558

[B251] Liwa A. C. Smart L. R. Frumkin A. Epstein H.-A. B. Fitzgerald D. W. Peck R. N. (2014). Traditional herbal medicine use among hypertensive patients in sub-Saharan Africa: a systematic review. Curr. Hypertens. Rep. 16 (6), 437. 24764197 10.1007/s11906-014-0437-9 PMC4076776

[B252] Logie C. H. (2020). Lessons learned from HIV can inform our approach to COVID-19 stigma. J. Int. AIDS Soc. 23, e25504. 32365283 10.1002/jia2.25504 PMC7197953

[B253] Lorgelly P. K. Adler A. (2020). Impact of a Global Pandemic on Health Technology Assessment. Appl. Health Econ. Health Policy 18 (3), 339–343 32377982 10.1007/s40258-020-00590-9 PMC7202921

[B254] Lubega M. Ekol J. E. (2020). Preparing communities to receive persons recently suspected or diagnosed with COVID-19. Pan Afr. Med. J. 35 (2), 21. 33623546 10.11604/pamj.supp.2020.35.2.23202 PMC7875737

[B255] Luo L. Jiang J. Wang C. Fitzgerald M. Hu W. Zhou Y. (2020). Analysis on herbal medicines utilized for treatment of COVID-19. Acta Pharm. Sin. B. 10.1016/j.apsb.2020.05.007 PMC7251357 32834949

[B256] Luo P. Liu Y. Qiu L. Liu X. Liu D. Li J. (2020). Tocilizumab treatment in COVID-19: A single center experience. J. Med. Virol. 92 (7), 814–818. 32253759 10.1002/jmv.25801 PMC7262125

[B257] Luo E. Zhang D. Luo H. Liu B. Zhao K. Zhao Y. (2020). Treatment efficacy analysis of traditional Chinese medicine for novel coronavirus pneumonia (COVID-19): an empirical study from Wuhan, Hubei Province, China. Chin. Med. 15 (34), 1–13. 32308732 10.1186/s13020-020-00317-x PMC7156896

[B258] Lusakatimes (2020). Government Suspended customs duties and VAT on additional medical supplies used in the fight against COVID-19. Available at: https://www.lusakatimes.com/2020/04/20/government-suspended-customs-duties-and-vat-on-additional-medical-supplies-used-in-the-fight-against-covid-19/.

[B259] Mabuka-Maroa J. (2020). Few clinical trials are done in Africa: COVID-19 shows why this urgently needs to change. Available at: https://theconversation.com/few-clinical-trials-are-done-in-africa-covid-19-shows-why-this-urgently-needs-to-change-135117.

[B260] MacQuilkan K. Baker P. Downey L. Ruiz F. Chalkidou K. Prinja S. (2018). Strengthening health technology assessment systems in the global south: a comparative analysis of the HTA journeys of China, India and South Africa. Glob. Health Action 11 (1), 1527556. 30326795 10.1080/16549716.2018.1527556 PMC6197020

[B261] Mahase E. (2020a). Doctors welcome critical care audit of covid-19 patient. BMJ 368, m1201. 32209544 10.1136/bmj.m1201

[B262] Mahase E. (2020b). Covid-19: EU states report 60% rise in emergency calls about domestic violence. BMJ 369, m1872. 32393463 10.1136/bmj.m1872

[B263] Manfredi G. Kotzalidis G. D. Sani G. Koukopoulos A. E. Savoja V. Lazanio S. (2010). Persistent interferon-ß-1b-induced psychosis in a patient with multiple sclerosis. Psychiatry Clin. Neurosci. 64 (5), 584–586. 20939157 10.1111/j.1440-1819.2010.02122.x

[B264] Marini J. J. Gattinoni L. (2020). Management of COVID-19 Respiratory Distress. JAMA. 10.1001/jama.2020.6825 32329799

[B265] Markovic-Pekovic V. Grubisa N. Burger J. Bojanic L. Godman B. (2017). Initiatives to Reduce Nonprescription Sales and Dispensing of Antibiotics: Findings and Implications. J. Res. Pharm. Pract. 6 (2), 120–125. 28616436 10.4103/jrpp.JRPP_17_12 PMC5463547

[B266] Marra L. P. Araujo V. E. Silva T. B. Diniz L. M. Guerra Junior A. A. Acurcio F. A. (2016). Clinical Effectiveness and Safety of Analog Glargine in Type 1 Diabetes: A Systematic Review and Meta-Analysis. Diabetes Ther. 7 (2), 241–258. 27048292 10.1007/s13300-016-0166-y PMC4900976

[B267] Martinez-Alvarez M. Jarde A. Usuf E. Brotherton H. Bittaye M. Samateh A. L. (2020). COVID-19 pandemic in west Africa. Lancet Global Health 8 (5), e631–e632. 32246918 10.1016/S2214-109X(20)30123-6 PMC7186549

[B268] Masango T. A. (2020). Misuse of chloroquine, azithromycin and lopinavir-ritonavir for the prevention and/or potential treatment of Covid-19. Available at: https://www.biznews.com/briefs/2020/03/24/pharmacy-body-anti-malaria-drugs-covid-19.

[B269] Masina L. (2020a). Malawi President Announces New Measures Against Coronavirus. Available at: https://www.voanews.com/covid-19-pandemic/malawi-president-announces-new-measures-against-coronavirus.

[B270] Masina L. (2020b). UN in Malawi Launches Emergency Appeal for COVID-19 Response. Available at: https://www.voanews.com/covid-19-pandemic/un-malawi-launches-emergency-appeal-covid-19-response.

[B271] Massonnaud C. Roux J. Crépey P. (2020). COVID-19: Forecasting short term hospital needs in France. MedRxiv preprint. Available at: https://www.medrxiv.org/content/10.1101/2020.03.16.20036939v1.full.pdf. 10.1101/2020.03.16.20036939

[B272] Matengo D. (2019). Cameroon declares polio emergency. Available at: https://africa.cgtn.com/2019/06/01/cameroon-declares-polio-emergency/.

[B273] Matos R. I. Chung K. K. (2020). DoD COVID-19 Practice Management Guide. Available at: http://www.med.umich.edu/surgery/mcccn/documents/DoD-COVID-19-Practice-Management-Guide-V10.

[B274] Matshediso M. (2020). Gauteng Health adds COVID-19 feature to Mpilo App. Available at: https://www.vukuzenzele.gov.za/gauteng-health-adds-covid-19-feature-mpilo-app.

[B275] Mbuagbaw L. Sivaramalingam B. Navarro T. Hobson N. Keepanasseril A. Wilczynski N. J. (2015). Interventions for Enhancing Adherence to Antiretroviral Therapy (ART): A Systematic Review of High Quality Studies. AIDS patient Care STDs 29 (5), 248–266. 25825938 10.1089/apc.2014.0308 PMC4410452

[B276] McCaffrey D. (2020). Analysis: Africa's unexpected COVID-19 figures. Available at: https://www.euronews.com/2020/05/12/analysis-africa-s-unexpected-covid-19-figures.

[B277] McMaster University. COVID-END (2020). COVID-19 Evidence Network to support Decision-making. Available at: https://www.mcmasterforum.org/networks/covid-end.

[B278] Media Foundation West Africa (2020). Curbing Misinformation in a COVID -19 Era, Ghana's Approach. Available at: https://www.mfwa.org/issues-in-focus/curbing-misinformation-in-a-covid-19-era-ghanas-approach/.

[B279] Medical Brief (2020a). Cape Town's BCG trials threatened by demands from advocacy group. Available at: https://www.medicalbrief.co.za/archives/cape-towns-bcg-trials-threatened-by-demands-from-advocacy-group/.

[B280] Medical Brief (2020b). Health Department 'taking steps' over shortage of antidepressants and antipsychotics. Available at: https://www.medicalbrief.co.za/archives/health-department-taking-steps-over-shortage-of-antidepressants-and-antipsychotics/.

[B281] Mehra M. R. Desai S. S. Ruschitzka F. Patel A. N. (2020). Retracted: Hydroxychloroquine or chloroquine with or without a macrolide for treatment of COVID-19: a multinational registry analysis. Lancet. 10.1016/S0140-6736(20)31180-6 PMC7255293 32450107

[B282] Mehta H. B. Ehrhardt S. Moore T. J. Segal J. B. Alexander G. C. (2020). Characteristics of registered clinical trials assessing treatments for COVID-19: a cross-sectional analysis. BMJ Open 10 (6), e039978. 10.1136/bmjopen-2020-039978 PMC7286283 32518212

[B283] Mehtar S. Preiser P. Lakhe N. A. Bousso A. TamFum J.-J. M. Kallay O. (2020). Limiting the spread of COVID-19 in Africa: one size mitigation strategies do not fit all countries. Lancet Global Health. 8 (7), e881–e883. 32530422 10.1016/S2214-109X(20)30212-6 PMC7195296

[B284] Mendelson M. Matsoso M. (2015). The South African Antimicrobial Resistance Strategy Framework. AMR Control, 54–61.

[B285] Meng L. Qiu H. Wan L. Ai Y. Xue Z. Guo Q. (2020). Intubation and Ventilation amid the COVID-19 Outbreak: Wuhan's Experience. Anesthesiology 132 (6), 1317–1332. 32195705 10.1097/ALN.0000000000003296 PMC7155908

[B286] Mensah G. A. Roth G. A. Sampson U. K. Moran A. E. Feigin V. L. Forouzanfar M. H. (2015). Mortality from cardiovascular diseases in sub-Saharan Africa, 1990-2013: a systematic analysis of data from the Global Burden of Disease Study 2013. Cardiovasc. J. Afr. 26 (2 Suppl 1), S6–10. 25962950 10.5830/CVJA-2015-036 PMC4557490

[B287] Miljković N. Godman B. Kovačević M. Polidori P. Tzimis L. Hoppe-Tichy T. (2020). Prospective Risk Assessment of Medicine Shortages in Europe and Israel: Findings and Implications. Front. Pharmacol. 11 (357), 1–4. 32273845 10.3389/fphar.2020.00357 PMC7114887

[B288] Mining Review (2020). South's Africa COVID-19 PPE shortage being addressed. Available at: https://www.miningreview.com/health-and-safety/souths-africa-covid-19-ppe-shortage-being-addressed/.

[B289] Ministry of Communications, Republic of Ghana (2020). Launch of GH COVID-19 Tracker App. Available at: https://www.moc.gov.gh/launch-gh-covid-19-tracker-app.

[B290] Ministry of Health, VietNam (2020). Hướng dẫn chẩn đoán và điều trị viêm đường hô hấp cấp do SARS-CoV-2 (COVID-19) phiên bản lần thứ 3. Available at: https://kcb.vn/huong-dan-chan-doan-va-dieu-tri-viem-duong-ho-hap-cap-do-sar-cov-2-covid-19-phien-ban-lan-thu-3.html.

[B291] Mitjà O. Clotet B. (2020). Use of antiviral drugs to reduce COVID-19 transmission. Lancet Global Health 8 (5), e639–ee40. 32199468 10.1016/S2214-109X(20)30114-5 PMC7104000

[B292] Modisakeng C. Matlala M. Godman B. Meyer J. C. (2020). Medicine shortages and challenges with the procurement process among public sector hospitals in South Africa; findings and implications. BMC Health Serv. Res. 20 (1), 234. 32192481 10.1186/s12913-020-05080-1 PMC7082963

[B293] Monteleone G. Sarzi-Puttini P. C. Ardizzone S. (2020). Preventing COVID-19-induced pneumonia with anticytokine therapy. Lancet Rheumatol. 2 (5), E255–E256. 10.1016/S2665-9913(20)30092-8 PMC7193140 32368737

[B294] Moore W. G. (2020). Curfews are a safer plan than total lockdowns to slow Covid-19's spread in informal economies. Available at: https://qz.com/africa/1836458/curfews-not-lockdowns-will-slow-covid-19-spread-in-africa/.

[B295] Moorkens E. Vulto A. G. Huys I. Dylst P. Godman B. Keuerleber S. (2017). Policies for biosimilar uptake in Europe: An overview. PLoS One 12 (12), e0190147. 29284064 10.1371/journal.pone.0190147 PMC5746224

[B296] MSF (2019). A multidisciplinary approach to stem the spread of cholera. Available at: https://www.msf.org/how-stem-spread-cholera-cameroon-and-lake-chad-region.

[B297] MSF (2020). Eswatini: Responding to COVID-19 in a country already fighting a dual HIV/TB epidemic. Available at: https://www.msf.org/responding-covid-19-eswatini.

[B298] Mueller D. Tivey D. Croce D. (2017). Health-technology assessment: Its role in strengthening health systems in developing countries. South. Afr. J. Public Health 2 (1), 6–11.

[B299] Mukokinya M. Opanga S. Oluka M. Godman B. (2018). Dispensing of antimicrobials in Kenya: A cross-sectional pilot study and its implications. J. Res. Pharm. Pract. 7 (2), 77–82. 30050960 10.4103/jrpp.JRPP_17_88 PMC6036869

[B300] Mules I. (2020). Tanzania under fire from WHO for lackluster response to COVID-19 pandemic. Available at: https://www.dw.com/en/tanzania-under-fire-from-who-for-lackluster-response-to-covid-19-pandemic/a-53304699.

[B301] Muraya J. (2020). Govt turns spotlight on refugee camps as COVID-19 hits 490 in Kenya. Available at: https://www.capitalfm.co.ke/news/2020/05/govt-turns-spotlight-on-refugee-camps-as-covid-19-hits-490-in-kenya/.

[B302] Murthy S. Leligdowicz A. Adhikari N. K. (2015). Intensive care unit capacity in low-income countries: a systematic review. PLoS One 10 (1), e0116949. 25617837 10.1371/journal.pone.0116949 PMC4305307

[B303] Mutahi B. (2020). Coronavirus: The fear of being sentenced to a Kenyan quarantine centre. Available at: https://www.bbc.co.uk/news/world-africa-52326316.

[B304] Mwai P. Giles C. (2020). Coronavirus in Tanzania: What do we know? Available at: https://www.bbc.co.uk/news/world-africa-52723594.

[B305] N Gage Consulting (2017). Egypt's Pharmaceutical Sector Following Bold Economic Reforms: Challenges and Opportunities. Available at: https://www.ngage-consulting.com/downloads/Pharmaceutical_PDF_Final_Version_K_and_A.pdf.

[B306] Nachimuthu S. Vijayalakshmi R. Sudha M. Viswanathan V. (2020). Coping with diabetes during the COVID - 19 lockdown in India: Results of an online pilot survey. Diabetes Metab. Syndrome 14 (4), 579–582. 10.1016/j.dsx.2020.04.053 PMC7211739 32416527

[B307] Neil S. Campbell E. M. (2020). Fake Science: XMRV, COVID-19 And The Toxic Legacy of Dr Judy Mikovits. AIDS Res. Hum. Retrov. 36 (7), 545–549 . 10.1089/aid.2020.0095 PMC7398426 32414291

[B308] Newton P. N. Bond K. C. (2020). COVID-19 and risks to the supply and quality of tests, drugs, and vaccines. Lancet Global Health. 8 (6), e754–e755. 32278364 10.1016/S2214-109X(20)30136-4 PMC7158941

[B309] Ng Y. Li Z. Chua Y. X. Chaw W. L. Zhao Z. Er B. (2020). Evaluation of the Effectiveness of Surveillance and Containment Measures for the First 100 Patients with COVID-19 in Singapore - January 2-February 29, 2020. MMWR Morbid. Mortal. Wkly. Rep. 69 (11), 307–311. 10.15585/mmwr.mm6911e1 PMC7739977 32191691

[B310] Nga L. Phuong L. Anh P. (2020). Hanoi man OD's on rumored malaria drug cure for Covid-19. 23 March 2020. Available at: https://e.vnexpress.net/news/news/hanoi-man-od-s-on-rumored-malaria-drug-cure-for-covid-19-4073488.html

[B311] Nicholas T. Mandaah F. V. Esemu S. N. Vanessa A. B. T. Gilchrist K. T. D. Vanessa L. F. (2020). COVID-19 knowledge, attitudes and practices in a conflict affected area of the South West Region of Cameroon. PAMJ 35 (2), 34. 10.11604/pamj.supp.2020.35.2.22963 33623559 PMC7875723

[B312] Nigeria CDC (2018). A home coming: My visit to Liberia's new National Public Health Institute. Available at: https://ncdc.gov.ng/blog/post/29/?t=a-home-coming:-my-visit-to-liberia%E2%80%99s-new-national-public-health-institute.

[B313] Nigeria Centre for Disease Control (2020a). Guidelines and Protocols - National Interim Guidelines for Clinical Management of COVID-19. Available at: https://ncdc.gov.ng/themes/common/docs/protocols/177_1584210847.pdf.

[B314] Nigeria Centre for Disease Control (2020b). An update of Lassa fever outbreak in Nigeria. Available at: https://ncdc.gov.ng/diseases/sitreps/?cat=5&name=An%20update%20of%20Lassa%20fever%20outbreak%20in%20Nigeria.

[B315] NIH ClinicalTrials.gov (2020a). BCG Vaccination for Healthcare Workers in COVID-19 Pandemic. Available at: https://clinicaltrials.gov/ct2/show/NCT04379336?cond=COVID-19&cntry=ZA&draw=2.

[B316] NIH ClinicalTrials.gov (2020b). COVID-19 Vaccine (ChAdOx1 nCoV-19) Trial in South African Adults With and Without HIV-infection. Available at: https://clinicaltrials.gov/ct2/show/NCT04444674?cond=COVID-19&cntry=ZA&draw=2&rank=7.

[B317] NIH (2020a). The National Institutes of Health COVID-19 Treatment Guidelines Panel Provides Recommendations for Dexamethasone in Patients with COVID-19. 25 June 2020. Available at: https://www.covid19treatmentguidelines.nih.gov/dexamethasone/.

[B318] NIH (2020b). NIH Clinical Trial Shows Remdesivir Accelerates Recovery from Advanced COVID-19. 29 April 2020. Available at: https://www.niaid.nih.gov/news-events/nih-clinical-trial-shows-remdesivir-accelerates-recovery-advanced-covid-19.

[B319] NIH (2020c). NIH halts clinical trial of hydroxychloroquine. 20 June 2020. Available at: https://www.nhlbi.nih.gov/news/2020/nih-halts-clinical-trial-hydroxychloroquine.

[B320] Nkeck J. R. Tsafack E. E. Ndoadoumgue A. L. Endomba F. T. (2020). An alert on the incautious use of herbal medicines by sub-Saharan African populations to fight against the COVID-19. PAMJ 35 (2), 26. 10.11604/pamj.supp.2020.35.2.23161 33623551 PMC7875753

[B321] Nkengasong J. N. Mankoula W. (2020). Looming threat of COVID-19 infection in Africa: act collectively, and fast. Lancet 395 (10227), 841–842. 32113508 10.1016/S0140-6736(20)30464-5 PMC7124371

[B322] Nordling N. (2020). Unproven herbal remedy against COVID-19 could fuel drug-resistant malaria, scientists warn. Available at: https://www.sciencemag.org/news/2020/05/unproven-herbal-remedy-against-covid-19-could-fuel-drug-resistant-malaria-scientists.

[B323] Nussbaumer-Streit B. Mayr V. Dobrescu A. I. Chapman A. Persad E. Klerings I. (2020). Quarantine alone or in combination with other public health measures to control COVID-19: a rapid review. Cochrane Database Syst. Rev. 4, Cd013574. 32267544 10.1002/14651858.CD013574 PMC7141753

[B324] Nyaira S. (2020). Ways Africa's Free Trade Area could help mitigate effects of COVID-19. Available at: https://www.un.org/africarenewal/web-features/coronavirus/ways-africa%E2%80%99s-free-trade-area-could-help-mitigate-effects-covid-19.

[B325] Nyavor G. (2020). Breakthrough as Ghana researchers develop rapid diagnostic testing for Covid-19. Available at: https://www.myjoyonline.com/news/health/breakthrough-as-ghana-researchers-develop-rapid-diagnostic-testing-for-covid-19/.

[B326] O'Neill L. A. J. Netea M. G. (2020). BCG-induced trained immunity: can it offer protection against COVID-19? Nat. Rev. Immunol. 20 (6), 335–337. 32393823 10.1038/s41577-020-0337-y PMC7212510

[B327] OCHA Zimbabwe (2020). Situation Reports Highlights. 7 May 2020. Available at: https://reliefweb.int/sites/reliefweb.int/files/resources/Situation%20Report%20-%20Zimbabwe%20-%206%20May%202020.pdf.

[B328] OCHA (2020). Sudan Situation Report Updated. 19 March 2020. Available at: https://reliefweb.int/sites/reliefweb.int/files/resources/Situation%20Report%20-%20Sudan%20-%2019%20Mar%202020.pdf.

[B329] OECD (2020). The Covid-19 Crisis in Egypt. Available at: https://www.oecd.org/mena/competitiveness/The-Covid-19-Crisis-in-Egypt.pdf.

[B330] Okonjo-Iweala N. Coulibaly B. S. Thiam T. Kaberuka D. Songwe V. Masiyiwa S. (2020). Africa needs debt relief to fight COVID-19. Available at: https://www.brookings.edu/opinions/africa-needs-debt-relief-to-fight-covid-19/.

[B331] Okwen P. M. Maweu I. Grimmer K. Margarita Dizon J. (2019). Evaluation of all African clinical practice guidelines for hypertension: Quality and opportunities for improvement. J. Eval. Clin. Pract. 25 (4), 565–574. 29901241 10.1111/jep.12954

[B332] Olafusi E. (2020). The World Health Organisation (WHO) says Nigeria has expressed interest to be part of the global solidarity drug trial to combat COVID-19. Available at: https://www.thecable.ng/who-nigeria-has-expressed-interest-to-be-part-of-covid-19-drug-trials.

[B333] Ong S. E. Koh J. J. K. Toh S. E. S. Chia K. S. Balabanova D. McKee M. (2018). Assessing the influence of health systems on Type 2 Diabetes Mellitus awareness, treatment, adherence, and control: A systematic review. PLoS One 13 (3), e0195086. 29596495 10.1371/journal.pone.0195086 PMC5875848

[B334] Ongole J. J. Rossouw T. M. Fourie P. B. Stoltz A. C. Hugo J. Marcus T. S. (2020). Sustaining essential healthcare in Africa during the COVID19 pandemic. Available at: https://www.theunion.org/news-centre/news/body/IJTLD-June-0214_Ongole.pdf. 10.5588/ijtld.20.0214 32553005

[B335] Oni T. Youngblood E. Boulle A. McGrath N. Wilkinson R. J. Levitt N. S. (2015). Patterns of HIV, TB, and non-communicable disease multi-morbidity in peri-urban South Africa- a cross sectional study. BMC Infect. Dis. 15, 20. 25595711 10.1186/s12879-015-0750-1 PMC4300166

[B336] Outbreak News (2020). Cameroon monkeypox: Follow-up and more details. Available at: http://outbreaknewstoday.com/cameroon-monkeypox-follow-up-and-more-details-15866/.

[B337] Pan D. Sze S. Minhas J. S. Bangash M. N. Pareek N. Divall P. (2020). The impact of ethnicity on clinical outcomes in COVID-19: A systematic review. EClinicalMedicine 23, 100404. 32632416 10.1016/j.eclinm.2020.100404 PMC7267805

[B338] Paranjpe I. Fuster V. Lala A. Russak A. Glicksberg B. S. Levin M. A. (2020). Association of Treatment Dose Anticoagulation with In-Hospital Survival Among Hospitalized Patients with COVID-19. J. Am. Coll. Cardiol. 76 (1), 122–124. 32387623 10.1016/j.jacc.2020.05.001 PMC7202841

[B339] Pareek M. Bangash M. N. Pareek N. Pan D. Sze S. Minhas J. S. (2020). Ethnicity and COVID-19: an urgent public health research priority. Lancet 395 (10234), 1421–1422. 32330427 10.1016/S0140-6736(20)30922-3 PMC7173801

[B340] Parpia A. S. Ndeffo-Mbah M. L. Wenzel N. S. Galvani A. P. (2016). Effects of Response to 2014-2015 Ebola Outbreak on Deaths from Malaria, HIV/AIDS, and Tuberculosis, West Africa. Emerg. Infect. Dis. 22 (3), 433–441. 26886846 10.3201/eid2203.150977 PMC4766886

[B341] Pauloluniyi (2020). First African SARS-CoV-2 genome sequence from Nigerian COVID-19 case. Available at: http://virological.org/t/first-african-sars-cov-2-genome-sequence-from-nigerian-covid-19-case/421.

[B342] Payne C. (2020). COVID-19 in Africa. Nat. Hum. Behav. 4 (5), 436–437. 32246105 10.1038/s41562-020-0870-5

[B343] Pearson J. Nguyen P. (2020). Vietnam to ease nationwide coronavirus lockdown. Available at: https://uk.reuters.com/article/uk-health-coronavirus-vietnam/vietnam-to-ease-nationwide-coronavirus-lockdown-idUKKCN2241LD.

[B344] Perencevich E. N. Diekema D. J. Edmond M. B. (2020). Moving Personal Protective Equipment Into the Community: Face Shields and Containment of COVID-19. JAMA. 10.1001/jama.2020.7477 32347911

[B345] Perry Wilson F. (2020). COVID-19, Hydroxycloroquine, and the Death of Evidence-Based Medicine. Available at: https://www.methodsman.com/blog/covid-19-evidence.

[B346] Phiri C. (2020). African Countries Urged To Collaborate More In The Fight Against Covid-19. Available at: https://zambiareports.com/2020/04/30/african-countries-urged-collaborate-fight-covid-19/.

[B347] Pilling D. (2020). Low Covid-19 death toll raises hopes Africa may be spared worst. Available at: https://www.ft.com/content/e9cf5ed0-a590-4bd6-8c00-b41d0c4ae6e0.

[B348] Pitts P. J. (2019). Towards Meaningful Engagement for the Patient Voice. Patient - Patient-Centered Outc. Res. 12 (4), 361–363. 10.1007/s40271-019-00366-x 31165399

[B349] Pitts P. J. (2020). Our Most Powerful Weapon to Fight COVID-19: Patient Involvement. Patient - Patient-Centered Outc. Res. 13 (3), 255. 10.1007/s40271-020-00421-y PMC7160609 32301034

[B350] Politi D. (2020). Nigeria Reports Chloroquine Poisonings as Trump Keeps Pushing Drug Against Coronavirus. Available at: https://slate.com/news-and-politics/2020/03/nigeria-chloroquine-poisonings-trump-pushing-drug-coronavirus.html.

[B351] Pradhan D. Biswasroy P. Kumar Naik P. Ghosh G. Rath G. (2020). A Review of Current Interventions for COVID-19 Prevention. Arch. Med. Res. 51 (5), 363–374. 32409144 10.1016/j.arcmed.2020.04.020 PMC7190516

[B352] President Republic of Kenya (2020a). Africa To Pursue Loan Waivers As Safeguard Against Adverse Economic Impact Of Coronavirus. Available at: https://www.president.go.ke/2020/04/04/africa-to-pursue-loan-waivers-as-safeguard-against-adverse-economic-impact-of-coronavirus/.

[B353] President Republic of Kenya (2020b)Presidential Address on The State Interventions to Cushion Kenyans Against Economic Effects Of Covid-19 Pandemic on 25th March, 2020. Available at: https://www.president.go.ke/2020/03/25/presidential-address-on-the-state-interventions-to-cushion-kenyans-against-economic-effects-of-covid-19-pandemic-on-25th-march-2020/.

[B354] Prokunina-Olsson L. Alphonse N. Dickenson R. E. Durbin J. E. Glenn J. S. Hartmann R. (2020). COVID-19 and emerging viral infections: The case for interferon lambda. J. Exp. Med. 217 (5), 1–4. 10.1084/jem.20200653 PMC7155807 32289152

[B355] Public Health England (2020). Disparities in the risk and outcomes of COVID-19. Available at: https://assets.publishing.service.gov.uk/government/uploads/system/uploads/attachment_data/file/892085/disparities_review.pdf.

[B356] Rajendran D. K. Rajagopal V. Alagumanian S. Santhosh Kumar T. Sathiya Prabhakaran S. P. Kasilingam D. (2020). Systematic literature review on novel corona virus SARS-CoV-2: a threat to human era. VirusDisease 31 (2), 161–173. 32656310 10.1007/s13337-020-00604-z PMC7288266

[B357] Rajkumar R. P. (2020). COVID-19 and mental health: A review of the existing literature. Asian J. Psychiatry 52, 102066. 10.1016/j.ajp.2020.102066 PMC7151415 32302935

[B358] Ramalingam S. Graham C. Dove J. Morrice L. Sheikh A. (2019). A pilot, open labelled, randomised controlled trial of hypertonic saline nasal irrigation and gargling for the common cold. Sci. Rep. 9 (1), 1015. 30705369 10.1038/s41598-018-37703-3 PMC6355924

[B359] Ramalingam S. Graham C. Dove J. Morrice L. Sheikh A. (2020). Hypertonic saline nasal irrigation and gargling should be considered as a treatment option for COVID-19. J. Global Health 10 (1), 010332. 10.7189/jogh.10.010332 PMC7193539 32395245

[B360] Reade M. C. Finfer S. (2014). Sedation and delirium in the intensive care unit. N Engl. J. Med. 370 (5), 444–454. 24476433 10.1056/NEJMra1208705

[B361] Recovery Collaborative Group Horby P. Lim W.S. Emberson Mafham M. JR Bell J.L. Linsell L. (2020). Dexamethasone in Hospitalized Patients with Covid-19 - Preliminary Report. N Engl J Med. 10.1056/NEJMoa2021436 PMC7383595 32678530

[B362] Recovery Trial (2020a). No clinical benefit from use of hydroxychloroquine in hospitalised patients with COVID-19. Available at: https://www.recoverytrial.net/news/statement-from-the-chief-investigators-of-the-randomised-evaluation-of-covid-19-therapy-recovery-trial-on-hydroxychloroquine-5-june-2020-no-clinical-benefit-from-use-of-hydroxychloroquine-in-hospitalised-patients-with-covid-19.

[B363] Recovery Trial (2020b). Statement from the Chief Investigators of the Randomised Evaluation of COVid-19 thERapY (RECOVERY) Trial on lopinavir-ritonavir, 29 June 2020. No clinical benefit from use of lopinavir-ritonavir in hospitalised COVID-19 patients studied in RECOVERY. Available at: https://www.recoverytrial.net/files/lopinavir-ritonavir-recovery-statement-29062020_final.pdf.

[B364] Recovery Trial (2020a). No clinical benefit from use of hydroxychloroquine in hospitalised patients with COVID-19. Available at: https://www.recoverytrial.net/news/statement-from-the-chief-investigators-of-the-randomised-evaluation-of-covid-19-therapy-recovery-trial-on-hydroxychloroquine-5-june-2020-no-clinical-benefit-from-use-of-hydroxychloroquine-in-hospitalised-patients-with-covid-19.

[B365] Rees V. Dexamethasone to be available on NHS as COVID-19 treatment. 22 June 2020. Available at: https://www.europeanpharmaceuticalreview.com/news/121560/dexamethasone-to-be-available-on-nhs-as-covid-19-treatment/

[B366] ReliefWeb (2020a). Democratic Republic of Congo: Coronavirus (COVID-19) Situation Report 5. Available at: https://reliefweb.int/report/democratic-republic-congo/democratic-republic-congo-coronavirus-covid-19-situation-report-5.

[B367] ReliefWeb (2020b). Namibia launches COVID-19 Communication Centre. Available at: https://reliefweb.int/report/namibia/namibia-launches-covid-19-communication-centre.

[B368] Ren Y. Zhou Y. Qian W. Zhang X. Liu Z. Wang R. (2020). Letter to the Editor “A longitudinal study on the mental health of general population during the COVID-19 epidemic in China”. Brain Behav. Immun. 87, 132–133. 32387510 10.1016/j.bbi.2020.05.004 PMC7201232

[B369] Republic of South Africa Government Gazette (2020). Exclusion Of Schedule 2, Schedule 3 And Schedule 4 Substances From The Operation Of Certain Provisions Of The Medicines And Related Substances Act, 1965 (Act No. 101 of 1965). April 2020. Available at: https://edit.laws.africa/works/akn/za/act/gn/2020/r481/media/publication/za-act-gn-2020-r481-publication-document.pdf.

[B370] Review Online (2020). No lithium tablets: anti-depressant shortage looms. Available at: https://reviewonline.co.za/404809/no-lithium-tablets-anti-depressant-shortage-looms/.

[B371] Rich D. (2020). Covid-19: In Cameroon, chloroquine therapy hailed by French expert becomes state protocol. Available at: https://www.france24.com/en/20200503-covid-19-in-cameroon-a-chloroquine-therapy-hailed-by-french-expert-becomes-state-protocol.

[B372] Richardson S. Hirsch J. S. Narasimhan M. Crawford J. M. McGinn T. Davidson K. W. (2020). Presenting Characteristics, Comorbidities, and Outcomes Among 5700 Patients Hospitalized With COVID-19 in the New York City Area. JAMA 323 (20), 2052–2059. 32320003 10.1001/jama.2020.6775 PMC7177629

[B373] Rios-González C. M. (2020). Knowledge, attitudes and practices towards COVID-19 in Paraguayans during outbreaks: a quick online survey. Available at: https://preprints.scielo.org/index.php/scielo/preprint/download/149/179/164

[B374] Roberto M. (2020). Testing COVID-19 costs KSh 100 in Senegal while Kenyan hospital charges KSh 10k for same service. Available at: https://www.msn.com/en-xl/news/other/testing-covid-19-costs-ksh-100-in-senegal-while-kenyan-hospital-charges-ksh-10k-for-same-service/ar-BB13gPE3.

[B375] Rosenberg E. S. Dufort E. M. Udo T. Wilberschied L. A. Kumar J. Tesoriero J. (2020). Association of Treatment With Hydroxychloroquine or Azithromycin With In-Hospital Mortality in Patients With COVID-19 in New York State. JAMA 323 (24), 2493–2502. 32392282 10.1001/jama.2020.8630 PMC7215635

[B376] Roumier M. Paule R. Groh M. Vallee A. Ackermann F. (2020). Interleukin-6 blockade for severe COVID-19. medrxiv 04 (20), 20061861.

[B377] Roussi A. Maxmen A. (2020). African nations missing from coronavirus trials. Nature. 10.1038/d41586-020-01010-7 32242116

[B378] Rubin E. J. Harrington D. P. Hogan J. W. Gatsonis C. Baden L. R. Hamel M. B. (2020). The Urgency of Care during the Covid-19 Pandemic - Learning as We Go. N Engl. J. Med. 382 (25), 2461–2462. 32379956 10.1056/NEJMe2015903 PMC7224605

[B379] Ryan D. H. Ravussin E. Heymsfield S. (2020). COVID 19 and the Patient with Obesity - The Editors Speak Out. Obesity 28 (5), 847. 32237212 10.1002/oby.22808 PMC7228389

[B380] Saba A. Jika T. (2020). Eastern Cape's PPE shortage endangers healthcare workers. Available at: https://mg.co.za/article/2020-04-23-eastern-capes-ppe-shortage-endangers-healthcare-workers/

[B381] SADC (2020). Statement by the SADC Executive Secretary, H.E. Dr Stergomena Lawrence Tax on Covid-19 and Gender Based Violence and Domestic Violence. Available at: https://www.sadc.int/news-events/news/statement-sadc-executive-secretary-he-dr-stergomena-lawrence-tax-covid-19-and-gender-based-violence-and-domestic-violence/.

[B382] SAHPRA (2020a). SAHPRA cautions against medicine stockpiling including Chloroquine containing products. Available at: http://www.sahpra.org.za/wp-content/uploads/2020/03/SAHPRA-communique_Chloroquine-Stockpiling_23032020.pdf.

[B383] SAHPRA (2020b). SAHPRA STATEMENT: BCG Vaccination and COVID-19 Disease. Available at: http://www.sahpra.org.za/wp-content/uploads/2020/04/SAHPRA-STATEMENT_BCGVaccinations-and-COVID-19.pdf.

[B384] Saleem Z. Godman B. Hassali M. A. Hashmi F. K. Azhar F. Rehman I. U. (2019). Point prevalence surveys of health-care-associated infections: a systematic review. Pathog. Global Health 1–15. 10.1080/20477724.2019.1632070 PMC6758614 31215326

[B385] Salum G. A. Rehmenklau J. F. Csordas M. C. Pereira F. P. Castan J. U. Ferreira A. B. (2020). Supporting people with severe mental health conditions during the COVID-19 pandemic: considerations for low- and middle-income countries using telehealth case management. Rev. Bras. Psiquiatria. 10.1590/1516-4446-2020-1078 PMC7430400 32578690

[B386] Sanders J. M. Monogue M. L. Jodlowski T. Z. Cutrell J. B. (2020). Pharmacologic Treatments for Coronavirus Disease 2019 (COVID-19): A Review. JAMA. 10.1001/jama.2020.6019 32282022

[B387] SAMRC (2020). COVID-19 research - Systematic Reviews to inform Public Health Guidance on COVID-19. Available at: https://www.samrc.ac.za/intramural-research-units/covid-19-research.

[B388] SAPC (2020). South African Pharmacy Council discourages panic-fuelled bulk purchases of essential supplies and medicinal items amid the coronavirus outbreak. Media Statement. 22 March 2020. Available at: https://www.sapc.za.org/Media/Default/Documents/SAPC%20Media%20Statement_Panic-fueled%20purchases.pdf.

[B389] Sarma P. Kaur H. Kumar H. Mahendru D. Avti P. Bhattacharyya A. (2020). Virological and clinical cure in COVID-19 patients treated with hydroxychloroquine: A systematic review and meta-analysis. J. Med. Virol. 92 (7), 776–785. 32297988 10.1002/jmv.25898 PMC7262144

[B390] Scavone C. Brusco S. Bertini M. Sportiello L. Rafaniello C. Zoccoli A. (2020). Current pharmacological treatments for COVID-19: what's next? Br. J. Pharmacol. 10.1111/bph.15072 PMC7264618 32329520

[B391] Schaaf H. S. du Preez K. Kruger M. Solomons R. Taljaard J. J. Rabie H. (2020). Bacille Calmette-Guérin (BCG) vaccine and the COVID-19 pandemic: responsible stewardship is needed. Available at: https://www.theunion.org/news-centre/news/body/IJTLD-0267_Schaaf.pdf. 10.5588/ijtld.20.0267 32718410

[B392] Schellack N. Bronkhorst E. Coetzee R. Godman B. Gous A. G. S. Kolman S. (2018). SASOCP position statement on the pharmacist's role in antibiotic stewardship 2018. South Afr. J. Infect. Dis. 33 (1), 28–35.

[B393] School of Public Health Makerere University (2020). Inside the Makerere Covid-19 Innovation activities. Available at: https://sph.mak.ac.ug/news/inside-makerere-covid-19-innovation-activities.

[B394] Schroder M. Bossert A. Kersting M. Aeffner S. Coetzee J. Timme M. (2020). COVID-19 in Africa: outbreak despite interventions? MedRxiv preprint. Available at: https://www.medrxiv.org/content/10.1101/2020.04.24.20077891v1.full.pdf. 10.1101/2020.04.24.20077891.t PMC7925578 33654164

[B395] Sciama Y. (2020). Is France's president fueling the hype over an unproven coronavirus treatment? Available at: https://www.sciencemag.org/news/2020/04/france-s-president-fueling-hype-over-unproven-coronavirus-treatment.

[B396] Seaton A. (2020). COVID-19 and its impact on antimicrobial stewardship. Available at: https://revive.gardp.org/covid-19-and-its-impact-on-antimicrobial-stewardship/.

[B397] Serwornoo M. Abrokwah R. (2020). Ghana: Coronavirus and the media. Available at: https://en.ejo.ch/ethics-quality/ghana-coronavirus-and-the-media.

[B398] Shah M. Sachdeva M. Dodiuk-Gad R. P. (2020). COVID-19 and Racial Disparities. J. Am. Acad. Dermatol. 10.1016/j.jaad.2020.04.046 PMC7162783 32305444

[B399] Shalhoub S. (2020). Interferon beta-1b for COVID-19. Lancet 395 (10238), 1670–1671. 32401712 10.1016/S0140-6736(20)31101-6 PMC7211497

[B400] Shepherd B. van der Mark N. (2020). Beyond Lockdown: Africa's Options for Responding to COVID-19. Available at: https://www.chathamhouse.org/expert/comment/beyond-lockdown-africa-s-options-responding-covid-19.

[B401] Shi C. Wang C. Wang H. Yang C. Cai F. Zeng F. (2020). Clinical observations of low molecular weight heparin in relieving inflammation in COVID-19 patients: A retrospective cohort study. medrxiv. 10.1101/2020.03.28.20046144 PMC7719364 32881340

[B402] Simpson B. W. (2020). In COVID-19, the Africa CDC Faces Its Greatest Challenge. Available at: https://www.globalhealthnow.org/2020-03/covid-19-3-year-old-africa-cdc-faces-its-greatest-challenge.

[B403] Singh D. R. Sunuwar D. R. Karki K. Ghimire S. Shrestha N. (2020a). Knowledge and Perception Towards Universal Safety Precautions During Early Phase of the COVID-19 Outbreak in Nepal. J. Community Health, 1–7. 32405905 10.1007/s10900-020-00839-3 PMC7220640

[B404] Singh A. K. Singh A. Singh R. Misra A. (2020b). Hydroxychloroquine in patients with COVID-19: A Systematic Review and meta-analysis. Diabetes Metab. Syndrome 14 (4), 589–596. 10.1016/j.dsx.2020.05.017 PMC7215156 32417708

[B405] Smith G. (2020). Stamping out misinformation in Kenya's COVID-19 fight - Volunteers and activists work to diffuse spread of fake information, false 'remedies' as Kenya steps up pandemic battle. Available at: https://www.aljazeera.com/news/2020/04/stamping-misinformation-kenya-covid-19-fight-200424195805081.html.

[B406] So-Armah K. Freiberg M. S. (2018). HIV and Cardiovascular Disease: Update on Clinical Events, Special Populations, and Novel Biomarkers. Curr. HIV/AIDS Rep. 15 (3), 233–244. 29752699 10.1007/s11904-018-0400-5 PMC6230511

[B407] South African Government (2020a). President Ramaphosa launches the Africa Medical Supplies Platform to help fight COVID-19 Coronavirus Pandemic. Available at: https://www.gov.za/speeches/president-ramaphosa-launches-africa-medical-supplies-platform-help-fight-covid-19.

[B408] South African Government (2020b). Regulations and Guidelines - Coronavirus Covid-19. Available at: https://www.gov.za/coronavirus/guidelines.

[B409] South African Government (2020c). President Cyril Ramaphosa: Additional Coronavirus COVID-19 economic and social relief measures. 21 April 2020. Available at: https://www.gov.za/speeches/president-cyril-ramaphosa-additional-coronavirus-covid-19-economic-and-social-relief.

[B410] South African Government (2020d). Social grants - Coronavirus COVID-19. Available at: https://www.gov.za/coronavirus/socialgrants.

[B411] Southern African Health Technology Assessment Society (SAHTAS) (2019). http://www.htasa.org.za/.

[B412] Sterne J. A. C. Murthy S. Diaz J. V. Slutsky A. S. Villar J. Angus D. (2020). Association Between Administration of Systemic Corticosteroids and Mortality Among Critically Ill Patients With COVID-19: A Meta-analysis. *Jama* (EPrint). 10.1001/jama.2020.17023 PMC7489434 32876694

[B413] Takyi-Boadu C. (2020). Government Officials Donate 3 months Salaries to Help Fight Covid-19. Available at: https://dailyguidenetwork.com/government-officials-donate-3months-salaries-to-help-fight-covid-19/.

[B414] Tamam L. Yerdelen D. Ozpoyraz N. (2003). Psychosis associated with interferon alfa therapy for chronic hepatitis B. Ann. Pharmacother. 37 (3), 384–387. 12639168 10.1345/aph.1C266

[B415] Tang N. Bai H. Chen X. Gong J. Li D. Sun Z. (2020). Anticoagulant treatment is associated with decreased mortality in severe coronavirus disease 2019 patients with coagulopathy. J. Thromb. Haemostasis 18 (5), 1094–1099. 32220112 10.1111/jth.14817 PMC9906401

[B416] Tarkang E. E. (2020). The fight against COVID-19 in sub-Saharan Africa-a threat to the continuous management of HIV patients: application of the action areas of the Ottawa charter for health promotion. PAMJ 35 (2), 25. 10.11604/pamj.supp.2020.35.2.23224 33623550 PMC7875743

[B417] Tembo L. (2020). Coping with school closures during the COVID-19 pandemic - Keeping children learning is a priority. Available at: https://www.unicef.org/malawi/stories/coping-school-closures-during-covid-19-pandemic.

[B418] Thai P. Q. Rabaa M. A. Luong D. H. Tan D. Q. Quang T. D. Quach H.-L. (2020). The first 100 days of SARS-CoV-2 control in Vietnam. Clin Infect. Dis. 10.1093/cid/ciaa1130 PMC7454342 32738143

[B419] The East African (2020). Seven die, 134 treated after cholera outbreak in northern Kenya. Available at: https://www.theeastafrican.co.ke/scienceandhealth/Cholera-kills-7-in-northern-Kenya/3073694-5538830-c21ggo/index.html.

[B420] The Guardian (2020a). High hopes' drug for Covid-19 treatment failed in full trial. April 2020. Available at: https://www.msn.com/en-gb/news/coronavirus/high-hopes-drug-for-covid-19-treatment-failed-in-full-trial/ar-BB136z6g?ocid=spartandhp.

[B421] The Guardian (2020b). Coronavirus: world's biggest trial of drug to treat Covid-19 begins in UK. Available at: https://www.theguardian.com/world/2020/apr/17/world-biggest-drug-trial-covid-19-uk.

[B422] The Lancet Editorial (2020). Expression of concern: Hydroxychloroquine or chloroquine with or without a macrolide for treatment of COVID-19: a multinational registry analysis. Lancet (London England) 395 (10240), e102–e10e. 10.1016/S0140-6736(20)31290-3 PMC7269709 32504543

[B423] The Reportor (2020). Lesotho now doing own COVID-19 testing. 26 June 2020. Available at: https://www.pressreader.com/lesotho/the-reporter/20200626/281500753517045.

[B424] The World Bank (2020a). World Bank Group Supports Ghana's COVID-19 Response. Available at: https://www.worldbank.org/en/news/press-release/2020/04/02/world-bank-group-supports-ghanas-covid-19-response.

[B425] The World Bank (2020b). Kenya Receives $50 Million World Bank Group Support to Address COVID-19 Pandemic. Available at: https://www.worldbank.org/en/news/press-release/2020/04/02/kenya-receives-50-million-world-bank-group-support-to-address-covid-19-pandemic.

[B426] Thornicroft G. Rose D. Kassam A. Sartorius N. (2007). Stigma: ignorance, prejudice or discrimination? Br. J. Psychiatry J. Ment. sci. 190, 192–193. 10.1192/bjp.bp.106.025791 17329736

[B427] Thornton J. (2020). Covid-19: Keep essential malaria services going during pandemic, urges WHO. BMJ 369, m1637. 32327414 10.1136/bmj.m1637

[B428] Tih F. (2020). African countries ease COVID-19 lockdown restrictions - Governments say there is need to resume economic activities with imperative to contain virus. Available at: https://www.aa.com.tr/en/africa/african-countries-ease-covid-19-lockdown-restrictions/1827900.

[B429] Tilangi P. Desai D. Khan A. Soneja M. (2020). Hydroxychloroquine prophylaxis for high-risk COVID-19 contacts in India: a prudent approach. Lancet Infect. Dis. 10.1016/S1473-3099(20)30430-8 PMC7255125 32450054

[B430] Times of Swaziland (2020). Over 100 Companies to Lay Off Staff. Available at: http://www.times.co.sz/news/127933-over-100-companies-to-lay-off-staff.html.

[B431] Tomlinson C. (2020). Covid-19: Is South Africa prepared for medicine shortages? (City Press). Available at: https://city-press.news24.com/News/covid-19-is-south-africa-prepared-for-medicine-shortages-20200316-2.

[B432] Tomazini B. M. Maia I. S. Cavalcanti A. B. Berwanger O. Rosa R. G. Veiga V. C. (2020). Effect of Dexamethasone on Days Alive and Ventilator-Free in Patients With Moderate or Severe Acute Respiratory Distress Syndrome and COVID-19: The CoDEX Randomized Clinical Trial. Jama (EPrint). 10.1001/jama.2020.17021 PMC7489411 32876695

[B433] Toniati P. Piva S. Cattalini M. Garrafa E. Regola F. Castelli F. (2020). Tocilizumab for the treatment of severe COVID-19 pneumonia with hyperinflammatory syndrome and acute respiratory failure: A single center study of 100 patients in Brescia, Italy. Autoimmun. Rev. 19 (7), 102568. 32376398 10.1016/j.autrev.2020.102568 PMC7252115

[B434] TradingEconomics (2020). Swaziland Interest Rate. Available at: https://tradingeconomics.com/swaziland/interest-rate.

[B435] Tu Y. F. Chien C. S. Yarmishyn A. A. Lin Y. Y. Luo Y. H. Lin Y. T. (2020). A Review of SARS-CoV-2 and the Ongoing Clinical Trials. Int. J. Mol. Sci. 21 (7), 1–19. 10.3390/ijms21072657 PMC7177898 32290293

[B436] UK Department of Health and Social Care (2020). Clinical trial approved to help the NHS treat COVID-19 patients using plasma. April 2020. Available at: https://www.gov.uk/government/news/clinical-trial-approved-to-help-the-nhs-treat-covid-19-patients-using-plasma.

[B437] UK Medicines & Healthcare Products Regulatory Agency EAMS 11972/0001 Remdesivir 100 mg powder for concentrate for solution for infusion. May 2020. Available at: https://www.gov.uk/government/publications/early-access-to-medicines-scheme-eams-scientific-opinion-remdesivir-in-the-treatment-of-patients-hospitalised-with-suspected-or-laboratory-confirme/treatment-protocol-for-healthcare-professionals_eams-119720001-remdesivir-100-mg-powder-for-concentrate-for-solution-for-infusion.

[B438] UN News (2020). Vaccine bottlenecks from COVID lockdown put children's lives at stake: UNICEF. Available at: https://news.un.org/en/story/2020/05/1063002.

[B439] UNAIDS (2019) Global HIV & AIDS statistics — 2019 fact sheet. Available at: https://www.unaids.org/sites/default/files/media_asset/UNAIDS_FactSheet_en.pdf.

[B440] Ung C. O. L. (2020). Community pharmacist in public health emergencies: Quick to action against the coronavirus 2019-nCoV outbreak. Res. Soc. Adm. Pharm. 16 (4), 583–586. 10.1016/j.sapharm.2020.02.003 PMC7129623 32081569

[B441] UNHCR (2020). Across West Africa dual challenge of conflict and coronavirus threatens millions of people. Available at: https://www.unhcr.org/uk/news/latest/2020/4/5e99b5074/across-west-africa-dual-challenge-conflict-coronavirus-threatens-millions.html.

[B442] UNICEF Malawi (2020) UNICEF Malawi Supply Update on Coronavirus Disease (COVID-19) Response - Prepositioned and locally procured supplies provide critical input to Malawi response in combating COVID-19. Available at: https://www.unicef.org/malawi/coronavirus-disease-covid-19/unicef-malawi-supply-update-coronavirus-disease-covid-19-response.

[B443] UNICEF South Africa (2020). Immunization against vaccine-preventable diseases is essential to protect children African Vaccination Week 2020 focuses on the importance of immunization in a time of COVID-19. Available at: https://www.unicef.org/southafrica/stories/immunization-against-vaccine-preventable-diseases-essential-protect-children.

[B444] UNICEF (2020). COVID-19: Gavi and UNICEF to secure equipment and diagnostics for lower-income countries. Available at: https://www.unicef.org/press-releases/covid-19-gavi-and-unicef-secure-equipment-and-diagnostics-lower-income-countries.

[B445] United Nations Africa Renewal (2020) Coronavirus Updates and Resources. 7 May 2020. Available at: https://www.un.org/africarenewal/news/coronavirus/new-who-estimates-190-000-people-could-die-covid-19-africa-if-not-controlled.

[B446] United Nations Economic Commission for Africa (2020a). UN sets up regional knowledge hub to fight COVID-19. Available at: https://www.uneca.org/stories/un-sets-regional-knowledge-hub-fight-covid-19.

[B447] United Nations Economic Commission for Africa (2020b). COVID-19 in Africa: Protecting Lives and Economies. Available at: https://www.uneca.org/sites/default/files/PublicationFiles/eca_covid_report_en_24apr_web1.pdf.

[B448] United Nations Malawi (2020). COVID-19 Update. Available at: https://reliefweb.int/sites/reliefweb.int/files/resources/Malawi-COVID-19-Situation-Update-03.04.20.pdf.

[B449] United Nations (2020a). Policy Brief: COVID-19 and the Need for Action on Mental Health. Available at: https://www.un.org/sites/un2.un.org/files/un_policy_brief-covid_and_mental_health_final.pdf.

[B450] United Nations (2020b). Policy Brief: Impact of COVID-19 in Africa. Available at: https://www.un.org/sites/un2.un.org/files/sg_policy_brief_on_covid-19_impact_on_africa_may_2020.pdf.

[B451] University of Ghana News Release (2020). University of Ghana (UG) Scientists Sequence Genomes of Novel Coronavirus. Available at: https://www.ug.edu.gh/news/news-release-university-ghana-scientists-sequence-genomes-novel-coronavirus.

[B452] University of Nairobi (2020). UoN Ventilators, ready for production model. Available at: https://www.uonbi.ac.ke/news/uon-ventilators-ready-production-model.

[B453] US Food and Drug Administration (2020). Emergency Use Authorization (EUA) for emergency use of remdesivir for the treatment of hospitalized coronavirus disease (COVID-19) patients. Available at: https://www.fda.gov/media/137564/download.

[B454] US National Library of Medicine - ClinicalTrials.gov (2020a). Impact of Nasal Saline Irrigations on Viral Load in Patients With COVID-19. Available at: https://clinicaltrials.gov/ct2/show/NCT04347538.

[B455] US National Library of Medicine - ClinicalTrials.gov (2020b). Gargling and Nasal Rinses to Reduce Oro- and Nasopharyngeal Viral Load in Patients With COVID-19. Available at: https://clinicaltrials.gov/ct2/show/NCT04344236.

[B456] US National Library of Medicine (2020). ClinicalTrials.gov. Available at: https://clinicaltrials.gov/ct2/results?cond=COVID-19&cntry=EG&age_v=&gndr=&type=Intr&rslt=&Search=Apply.

[B457] van den Heever A. (2020). Projections on SA health system and whether there are enough hospital beds to cope. Available at: https://www.dailymaverick.co.za/article/2020-03-16-projections-on-sa-health-system-and-whether-there-enough-hospital-beds-to-cope/.

[B458] Vardavas C. I. Nikitara K. (2020). COVID-19 and smoking: A systematic review of the evidence. Tobacco Induced Dis. 18, 20. 10.18332/tid/119324 PMC7083240 32206052

[B459] Vella Bonanno P. Bucsics A. Simoens S. Martin A. P. Oortwijn W. Gulbinovic J. (2019). Proposal for a regulation on health technology assessment in Europe - opinions of policy makers, payers and academics from the field of HTA. Expert Rev. Pharmacoecon. Outc. Res. 19 (3), 251–261. 10.1080/14737167.2019.1575730 30696372

[B460] Vellingiri B. Jayaramayya K. Iyer M. Narayanasamy A. Govindasamy V. Giridharan B. (2020). COVID-19: A promising cure for the global panic. Sci. Total Environ. 725, 138277. 32278175 10.1016/j.scitotenv.2020.138277 PMC7128376

[B461] Wang C. Pan R. Wan X. Tan Y. Xu L. Ho C. S. (2020a). Immediate Psychological Responses and Associated Factors during the Initial Stage of the 2019 Coronavirus Disease (COVID-19) Epidemic among the General Population in China. Int. J. Environ. Res. Public Health 17 (5) 1–25. 10.3390/ijerph17051729 PMC7084952 32155789

[B462] Wang C. Pan R. Wan X. Tan Y. Xu L. McIntyre R. S. (2020b). A longitudinal study on the mental health of general population during the COVID-19 epidemic in China. Brain Behav. Immun. 87, 40–48. 32298802 10.1016/j.bbi.2020.04.028 PMC7153528

[B463] Wang C. J. Ng C. Y. Brook R. H. (2020). Response to COVID-19 in Taiwan: Big Data Analytics, New Technology, and Proactive Testing. JAMA. 10.1001/jama.2020.3151 32125371

[B464] Wang Y. Zhang D. Du G. Du R. Zhao J. Jin Y. (2020). Remdesivir in adults with severe COVID-19: a randomised, double-blind, placebo-controlled, multicentre trial. Lancet 395 (10236), 1569–1578. 32423584 10.1016/S0140-6736(20)31022-9 PMC7190303

[B465] Warrington T. P. Bostwick J. M. (2006). Psychiatric adverse effects of corticosteroids. Mayo Clin. Proc. 81 (10), 1361–1367. 17036562 10.4065/81.10.1361

[B466] Webster P. (2020). Virtual health care in the era of COVID-19. Lancet 395 (10231), 1180–1181. 32278374 10.1016/S0140-6736(20)30818-7 PMC7146660

[B467] WHO Regional Office Africa (2020a). COVID-19 - Situation Update for the WHO African Region - 1 July 2020. External Situation Report 18. Available at: https://apps.who.int/iris/bitstream/handle/10665/332929/SITREP_COVID-19_WHOAFRO_20200701-eng.pdf.

[B468] WHO Regional Office Africa (2020b). COVID-19 in the WHO African Region. Available at: https://www.afro.who.int/health-topics/coronavirus-covid-19.

[B469] WHO Regional Office Africa (2020c). African countries move from COVID-19 readiness to response as many confirm cases. 29 April 2020. Available at: https://www.afro.who.int/health-topics/coronavirus-covid-19.

[B470] WHO Regional Office Africa (2020d). COVID-19 - External Situation Report 9. 29 April 2020. Available at: https://apps.who.int/iris/bitstream/handle/10665/331935/SITREP_COVID-19_WHOAFRO_20200429-eng.pdf.

[B471] WHO Coronavirus disease (COVID-19) Situation Report – 151. Available at: https://www.who.int/docs/default-source/coronaviruse/situation-reports/20200619-covid-19-sitrep-151.pdf?sfvrsn=8b23b56e_2.

[B472] WHO (2019a). Global Tuberculosis Report Executive Summary. Available at: https://www.who.int/tb/publications/global_report/tb19_Exec_Sum_15October2019.pdf?ua=1.

[B473] WHO (2019b). Global Tuberculosis Report (Full). Available at: https://apps.who.int/iris/bitstream/handle/10665/329368/9789241565714-eng.pdf?ua=1.

[B474] WHO (2019c) World Malaria Report 2019. Available at: https://www.who.int/publications-detail/world-malaria-report-2019.

[B475] WHO (2020a). Coronavirus disease (COVID-19) Situation Report – 163. Available at: https://www.who.int/docs/default-source/coronaviruse/situation-reports/20200701-covid-19-sitrep-163.pdf?sfvrsn=c202f05b_2.

[B476] WHO (2020b). Modes of transmission of virus causing COVID-19: implications for IPC precaution recommendations. Available at: https://www.who.int/news-room/commentaries/detail/modes-of-transmission-of-virus-causing-covid-19-implications-for-ipc-precaution-recommendations.

[B477] WHO (2020c) COVID-19 Strategy update. Available at: https://www.who.int/docs/default-source/coronaviruse/covid-strategy-update-14april2020.pdf?sfvrsn=29da3ba.

[B478] WHO (2020d). COVID-19 Strategic Preparedness and Response Plan - OPERATIONAL PLANNING GUIDELINES TO SUPPORT COUNTRY PREPAREDNESS AND RESPONSE. Available at: https://www.who.int/docs/default-source/coronaviruse/covid-19-sprp-unct-guidelines.pdf.

[B479] WHO (2020e). WHO discontinues hydroxychloroquine and lopinavir/ritonavir treatment arms for COVID-19. 4 July 2020. Available at: https://www.who.int/news-room/detail/04-07-2020-who-discontinues-hydroxychloroquine-and-lopinavir-ritonavir-treatment-arms-for-covid-19.

[B480] WHO (2020f). Bacille Calmette-Guérin (BCG) vaccination and COVID-19 - Scientific Brief. Available at: https://www.who.int/news-room/commentaries/detail/bacille-calmette-gu%C3%A9rin-(bcg)-vaccination-and-covid-19.

[B481] WHO (2020g). Mental health and psychosocial considerations during the COVID-19 outbreak. Available at: https://www.who.int/publications-detail/WHO-2019-nCoV-MentalHealth-2020.1.

[B482] WHO (2020h) WHO Director-General's opening remarks at the media briefing on COVID-19 - 25 May 2020. Available at: https://www.who.int/dg/speeches/detail/who-director-general-s-opening-remarks-at-the-media-briefing-on-covid-19—25-may-2020.

[B483] WHO (2020i). Emergency Ministerial meeting on COVID-19 organized by the African Union and the Africa Centres for Disease Control and Prevention. Available at: https://www.who.int/dg/speeches/detail/emergency-ministerial-meeting-on-covid-19-organized-by-the-african-union-and-the-africa-centres-for-disease-control-and-prevention.

[B484] WHO (2020j). Coronavirus disease (COVID-19) pandemic. Available at: https://www.who.int/emergencies/diseases/novel-coronavirus-2019.

[B485] WHO (2020k). Launch of the Lomé Initiative. Available at: https://www.who.int/dg/speeches/detail/launch-of-the-lom%C3%A9-initiative.

[B486] WHO (2020l). “Solidarity” clinical trial for COVID-19 treatments. Available at: https://www.who.int/emergencies/diseases/novel-coronavirus-2019/global-research-on-novel-coronavirus-2019-ncov/solidarity-clinical-trial-for-covid-19-treatments.

[B487] WHO (2020m). Ebola virus disease – Democratic Republic of the Congo. Available at: https://www.who.int/csr/don/23-April-2020-ebola-drc/en/.

[B488] WHO (2020n). Protecting lifesaving immunization services during COVID-19: New guidance from WHO. Available at: https://www.who.int/immunization/news_guidance_immunization_services_during_COVID-19/en/.

[B489] Wilkinson L. Grimsrud A. (2020). The time is now: expedited HIV differentiated service delivery during the COVID-19 pandemic. J. Int. AIDS Soc. 23, e25503. 32378345 10.1002/jia2.25503 PMC7203569

[B490] Williams F. M. K. Freidin M. B. Mangino M. Couvreur S. Visconti A. Bowyer R. C. E. (2020). Self-reported symptoms of covid-19 including symptoms most predictive of SARS-CoV2 infection, are heritable. MedRxiv preprint. Available at: https://www.medrxiv.org/content/10.1101/2020.04.22.20072124v2.full.pdf. 10.1101/2020.04.22.20072124 33558003

[B491] Wits University (2020a) The first Covid-19 vaccine trial in South Africa begins. 23 June 2020. Available at: https://www.wits.ac.za/covid19/covid19-news/latest/the-first-covid-19-vaccine-trial-in-south-africa-begins.html.

[B492] Wits University (2020b). Wits engineers make face shields to protect healthcare workers. Available at: https://www.wits.ac.za/covid19/covid19-news/latest/wits-engineers-make-face-shields-to-protect-healthcare-workers.html.

[B493] Woldesemayat E. M. (2020). Chronic Diseases Multimorbidity among Adult People Living with HIV at Hawassa University Comprehensive Specialized Hospital, Southern Ethiopia. Int. J. Chronic Dis. 2020, 2190395. 32099838 10.1155/2020/2190395 PMC6998747

[B494] World Bank (2020). South Sudan: World Bank Provides $7.6 Million in Support of Coronavirus Emergency Response. Available at: https://www.worldbank.org/en/news/press-release/2020/04/09/south-sudan-world-bank-provides-76-million-in-support-of-coronavirus-emergency-response.

[B495] World Food Programme (2020). COVID-19 will double number of people facing food crises unless swift action is taken. Available at: https://www.wfp.org/news/covid-19-will-double-number-people-facing-food-crises-unless-swift-action-taken.

[B496] World Health Organisation Europe (2020) Health Systems Respond to COVID-19 Technical Guidance 2 - Creating surge capacity for acute and intensive care - Recommendations for the WHO European Region . Available at: http://www.euro.who.int/:data/assets/pdf_file/0006/437469/TG2-CreatingSurgeAcuteICUcapacity-eng.pdf.

[B497] World Health Organisation (2019). Global Health Observatory data repository. Number of people (all ages) living with HIV - Estimates by WHO region. Available at: https://apps.who.int/gho/data/view.main.22100WHO?lang=en.

[B498] World Health Organisation (2020a). Naming the coronavirus disease (COVID-19) and the virus that causes it. Available at: https://www.who.int/emergencies/diseases/novel-coronavirus-2019/technical-guidance/naming-the-coronavirus-disease-(covid-2019)-and-the-virus-that-causes-it.

[B499] World Health Organisation (2020b). Operational considerations for case management of COVID-19 in health facility and community: interim guidance, 19 March 2020. Available at: https://apps.who.int/iris/handle/10665/331492.

[B500] World Health Organisation (2020c). The potential impact of health service disruptions on the burden of malaria: a modelling analysis for countries in sub-Saharan Africa. Available at: https://apps.who.int/iris/bitstream/handle/10665/331845/9789240004641-eng.pdf?sequence=1&isAllowed=y.

[B501] World Health Organization (2020d) Guiding principles for immunization activities during the COVID-19 pandemic: interim guidance, 26 March 2020. Available at: https://apps.who.int/iris/handle/10665/331590.

[B502] World Health Organisation (2020e). Medical Product Alert N°4/2020 - Falsified chloroquine products circulating in the WHO region of Africa. Available at: https://www.who.int/docs/default-source/essential-medicines/drug-alerts20/drug-alert-4/n-4-2020-falsified-chloroquine-en.pdf?sfvrsn=c4354802_6.

[B503] World Health Organisation (2020f). Coronavirus disease (COVID-19) advice for the public: Myth busters. Available at: https://www.who.int/emergencies/diseases/novel-coronavirus-2019/advice-for-public/myth-busters.

[B504] Worldometer (2020). African Countries by population. Available at: https://www.worldometers.info/population/countries-in-africa-by-population/.

[B505] Wu Z. McGoogan J. M. (2020). Characteristics of and Important Lessons From the Coronavirus Disease 2019 (COVID-19) Outbreak in China: Summary of a Report of 72 314 Cases From the Chinese Center for Disease Control and Prevention. JAMA 323 (13), 123942. 10.1001/jama.2020.2648 32091533

[B506] Wu F. Wang A. Liu M. Wang Q. Chen J. Xia S. (2020). Neutralizing antibody responses to SARS-CoV-2 in a COVID-19 recovered patient cohort and their implications. MedRxiv preprint. Available at: https://www.medrxiv.org/content/10.1101/2020.03.30.20047365v2.full.pdf. 10.1101/2020.03.30.20047365

[B507] Xiang Y. T. Yang Y. Li W. Zhang L. Zhang Q. Cheung T. (2020). Timely mental health care for the 2019 novel coronavirus outbreak is urgently needed. Lancet Psychiatry 7 (3), 228–229. 32032543 10.1016/S2215-0366(20)30046-8 PMC7128153

[B508] Xinhua (2020a). Uganda braces for economic impact of COVID-19. Available at: http://www.xinhuanet.com/english/2020-03/20/c_138898637.htm.

[B509] Xinhua (2020b). China's Jack Ma gives Zambia COVID-19 medical supplies. Available at: https://www.focac.org/eng/zfgx_4/jmhz/t1760745.htm.

[B510] Xu X. Ong Y. K. Wang D. Y. (2020). Role of adjunctive treatment strategies in COVID-19 and a review of international and national clinical guidelines. Mil. Med. Res. 7 (1), 22 . 32370766 10.1186/s40779-020-00251-x PMC7199873

[B511] Yang Y. (2020). Use of herbal drugs to treat COVID-19 should be with caution. Lancet 395 (10238), 1689–1690. 32422123 10.1016/S0140-6736(20)31143-0 PMC7228688

[B512] Yao H. Chen J. H. Xu Y. F. (2020). Patients with mental health disorders in the COVID-19 epidemic. Lancet Psychiatry 7 (4), e21. 32199510 10.1016/S2215-0366(20)30090-0 PMC7269717

[B513] Zhang C. Wu Z. Li J. W. Zhao H. Wang G. Q. (2020). The cytokine release syndrome (CRS) of severe COVID-19 and Interleukin-6 receptor (IL-6R) antagonist Tocilizumab may be the key to reduce the mortality. Int. J. Antimicrob. Agents 105954, 1–6. 10.1016/j.ijantimicag.2020.105954 PMC7118634 32234467

[B514] Zhang L. Yu J. Zhou Y. Shen M. Sun L. (2020). Becoming a Faithful Defender: Traditional Chinese Medicine against Coronavirus Disease 2019 (COVID-19). Am. J. Chin. Med. 48 (4), 763–777. 32349517 10.1142/S0192415X2050038X

[B515] Zheng Z. Peng F. Xu B. Zhao J. Liu H. Peng J. (2020). Risk factors of critical & mortal COVID-19 cases: A systematic literature review and meta-analysis. J. Infection. 10.1016/j.jinf.2020.04.021 PMC7177098 32335169

[B516] Zheng S. Q. Yang L. Zhou P. X. Li H. B. Liu F. Zhao R. S. (2020). Recommendations and guidance for providing pharmaceutical care services during COVID-19 pandemic: A China perspective. Res. Soc. Adm. Pharm. 10.1016/j.sapharm.2020.03.012 PMC7102520 32249102

[B517] Zhong H. Wang Y. Zhang Z. L. Liu Y. X. Le K. J. Cui M. (2020). Efficacy and safety of current therapeutic options for COVID-19 - lessons to be learnt from SARS and MERS epidemic: A systematic review and meta-analysis. Pharmacol. Res. 157, 104872. 32360583 10.1016/j.phrs.2020.104872 PMC7192121

[B518] Zhong B. L. Luo W. Li H. M. Zhang Q. Q. Liu X. G. Li W. T. (2020). Knowledge, attitudes, and practices towards COVID-19 among Chinese residents during the rapid rise period of the COVID-19 outbreak: a quick online cross-sectional survey. Int. J. Biol. Sci. 16 (10), 1745–1752. 32226294 10.7150/ijbs.45221 PMC7098034

[B519] Zhou F. Yu T. Du R. Fan G. Liu Y. Liu Z. (2020). Clinical course and risk factors for mortality of adult inpatients with COVID-19 in Wuhan, China: a retrospective cohort study. Lancet 395 (10229), 1054–1062. 32171076 10.1016/S0140-6736(20)30566-3 PMC7270627

[B520] Zhu Z. Lu Z. Xu T. Chen C. Yang G. Zha T. (2020). Arbidol monotherapy is superior to lopinavir/ritonavir in treating COVID-19. J. Infection 81 (1), e21–ee3. 10.1016/j.jinf.2020.03.060 PMC7195393 32283143

[B521] Zurek K. (2020). COVID-19: President Akufo-Addo lifts partial lockdown effective Monday. Available at: https://www.graphic.com.gh/news/general-news/prez-akufo-addo-covid-update-7.html.

