# Supplementary material for: Response to the Novel Corona Virus (COVID-19) Pandemic Across Africa: Successes, Challenges, and Implications for the Future
Source: Front Pharmacol. 2020 Sep 11;11:1205. doi: 10.3389/fphar.2020.01205 (PMC7533592; doi:10.3389/fphar.2020.01205)
Supplement: Supplementary file 1 [file DataSheet_1.pdf]

Appendix

Table 1A – Epidemiology of COVID-19 up to end June 2020 across Africa and developments over time

Country	Epidemiology of COVID-19 across Africa
Algeria	• By 14 April 2020, there were 2070 recorded cases and 326 recorded deaths in Algeria giving a CFR of 15.7% (1)<br>• This increased to 4838 recorded cases and 470 recorded deaths in Algeria by 5 May 2020 giving a CFR of 9.7% (2), with Algeria seen as having a high importation risk of COVID-19 from China (3)<br>• By 12 May there were 6067 recorded cases and 515 recorded deaths giving a CFR of 8.5% (4), and by 19 May there were 7377 recorded cases in Algeria and 561 recorded deaths giving a CFR of 7.6% (5)<br>• By 26 May, there were 8,697 reported cases and 617 recorded deaths giving a CFR of 7.1% (6)<br>• By 30 June, there were 13907 reported cases and 912 recorded deaths giving a CFR of 6.6% (7)
Botswana	• By 14 April 2020, there were 13 recorded cases and 1 recorded death in Botswana giving a CFR of 7.7 % (1)<br>• This increased to 23 recorded cases and still only one recorded death by 5 May 2020 giving a CFR of 4.3% (2)<br>• By 12 May 2020 there were 24 recorded cases with still only one recorded death giving a CFR of 4.2% (4), and by 19 May there were 25 recorded cases in Botswana with still only one recorded death giving a CFR of 4.0% (5)<br>• By 26 May, there were 35 reported cases and still only one recorded death giving a CFR of 2.9% (6)<br>• By 30 June, there were 227 reported cases and still only on recorded death giving a CFR of 0.4% (7)
Burkina Faso	• By 14 April 2020, there were 515 recorded cases and 28 recorded deaths in Burkina Faso giving a CFR of 5.4% (1)<br>• This increased to 689 recorded cases and 48 recorded deaths by 5 May 2020 giving a CFR of 7.0% (2)<br>• By 12 May 2020 there were 766 recorded cases with 51 recorded deaths giving a CFR of 6.7% (4), and by 19 May there were 806 recorded cases in Burkina Faso with 52 recorded deaths giving a CFR of 6.5% (5)<br>• By 26 May, there were 845 reported cases and 53 recorded deaths giving a CFR of 6.3% (6)<br>• By 30 June, there were 962 reported cases and still only 53 recorded deaths giving a CFR of 5.5% (7)
Cameroon	• By 14 April 2020, there were 855 recorded cases and 15 recorded deaths in Cameroon giving a CFR of 1.8% (1, 8)<br>• This increased to 2265 recorded cases and 108 recorded deaths by 5 May 2020 giving a CFR of 4.8% (2) with the first case recorded on 6 March 2020 (9, 10)<br>• By 12 May there were 2689 recorded cases with 125 recorded deaths giving a CFR of 4.6% (4), and by 19 May there were 3529 recorded cases in Cameroon with 140 recorded deaths giving a CFR of 4.0% (5)<br>• By 26 May, there were 5436 reported cases and 177 recorded deaths giving a CFR of 3.3% (6)<br>• By 30 June, there were 12592 reported cases and 313 recorded deaths giving a CFR of 2.5% (7)<br>• However, there are concerns may be an underestimate due to issues with the robustness of the testing system (9)
Democratic Republic of Congo	• The first recorded case in the Republic was on 10 March 2020 and by 14 April 2020 there were 254 recorded cases and 21 recorded deaths giving a CFR of 8.3% (1)

	• This increased to 705 recorded cases and 34 recorded deaths by 5 May 2020 giving a CFR of 4.8% (2)<br>• By 12 May 2020 there were 1169 recorded cases with 50 recorded deaths giving a case fatality ratio of 4.3% (4), and by 19 May there were 1731 recorded cases in the Republic with 60 recorded deaths giving a CFR of 3.5% (5)<br>• By 26 May, there were 2403 reported cases and 67 recorded deaths giving a CFR of 2.8% (6)<br>• By 30 June, there were 7039 reported cases and 169 recorded deaths giving a CFR of 2.4% (7)
Egypt	• As at 23 April, 2020, there were 27 central laboratories affiliated to the Egyptian Ministry of Health to test for COVID-19 with a total of 36 PCR equipment Egypt seen as having a high importation risk of COVID-19 from China (3)<br>• As of 23 April 2020, Egypt had undertaken 90,000 tests with 3891 positive cases, 1004 recovered and 287 deaths with a CFR of 7.4% (11, 12). This is likely to be an under-estimation of the total number of cases and an over-estimation of the CFR (13)<br>• As of 12 May, there were 9746 reported cases with 533 reported deaths (14), and of 19 May there were 12,764 reported cases and 645 recorded deaths in Egypt giving a CFR of 5.1% (15)<br>• By 27 May there were 18756 reported cases with 767 recorded deaths giving a CFR of 4.3% (16)<br>• By 30 June, there were 68311 reported cases and 2953 recorded deaths giving a CFR of 4.3% (17)
Eswatini (Swaziland)	Up to 21st April 2020:<br>• Total confirmed cases: 31<br>• Total deaths: 1 (CFR: 3.2%)<br>• Total recovered: 8<br>• Total under follow up: 157<br><br>As of 1 May 2020 (18):<br>• Tests conducted: 2262 (Mbabane Govt Hosp, Lubombo Referral Hospital with ongoing training to increase capacity)<br>• Total confirmed cases: 116 (Active no symptoms: 39, active mild: 43; active moderate to severe: 2; active severe 1; active clinical status pending: 1)<br>• Total recovered: 18<br>• Total deaths: 1<br><br>As of 5 May 2020, there were 119 recorded cases and one death giving a CFR of 0.8% (2). By 12 May, there were 184 recorded cases and 2 deaths giving a CFR of 1.1% (4) and by 19 May there were 208 recorded cases in Eswatini and still only 2 recorded deaths giving a CFR of 1.0% (5). By 26 May, there were 261 reported cases and still only 2 recorded deaths giving a CFR of 0.8% (6), and by 30 June, there were 812 reported cases and only 11 recorded deaths giving a CFR of 1.4% (7)<br><br>Screening for COVID-19 is helped by:<br>• Identification of positive cases through commissioned regional rapid response teams and screening at entry points of hospitals, with persons asked to isolate as a preventive measure to eliminate the risk of potential infection before screening<br>• Patients accessing the emergency toll-free line reporting symptoms of COVID-19<br>• Ongoing contact tracing<br>• Intensified screening at clinics close to informal crossings as well as for returned travellers
Ethiopia	• By 14 April 2020, there were 82 recorded cases and 3 recorded deaths in Ethiopia giving a CFR of 3.7% (1)

	• This increased to 145 recorded cases and 4 recorded deaths by 5 May 2020 giving a CFR of 2.8% (19)<br>• As of 12 May, there were 265 recorded cases with 5 recorded deaths giving a CFR of 1.9% (4), and by 19 May there were 365 recorded cases in Ethiopia with still only 5 recorded deaths giving a CFR of 1.4% (5)<br>• By 26 May, there were 701 reported cases and only 6 recorded deaths giving a CFR of 0.9% (6)<br>• However by 30 June, there were 5846 reported cases and 103 recorded deaths giving a CFR of 1.8% (7)
Ghana	In Ghana (19 April, 2020):<br>• Number of tests performed: over 60,000, with currently 2 universities and 1 reference laboratory in Ghana handling the testing (20)<br>• Number of positive tests: 1042<br>• Number recovered 99<br>• Number died: 9<br><br>The introduction of drones has facilitated testing in rural areas (21). As of 19th April, Ghana was the only country in Africa to have conducted more than sixty thousand tests, ranked first in Africa in terms of the number of administered tests per million people (20).<br><br>In Ghana (4 May 2020) there were (22):<br>• Number of positive tests: 2719 (61% male)<br>• Number of recoveries: 294<br>• Number of deaths: 18<br><br>• This increased to 5127 recorded cases with 22 recorded deaths by 12 May 2020 giving a CFR of 0.4% (4). By 19 May there were 6096 recorded cases in Ghana with 31 recorded deaths giving a CFR of 0.5% (5)<br>• By 26 May, there were 6964 reported cases and 32 recorded deaths giving a CFR of 0.5% (6)<br>• By 30 June, there were 17741 reported cases and 112 recorded deaths giving a CFR of 0.6% (7)
Kenya	• Kenya reported its first COVID-19 positive case on 12th March 2020, and by 14 April 2020, there were 216 recorded cases and 9 recorded deaths giving a CFR of 4.2% (1, 23)<br>• This increased to 535 recorded cases and 23 recorded deaths in Kenya by 5 May 2020 giving CFR of 4.3% (2)<br>• Between 13 March 2020 and 9 May 2020, there have been 649 reported cases, most of which have been in the capital city Nairobi and the coastal city Mombasa, with 30 deaths to date (24)<br>• This increased to 715 recorded cases with 36 recorded deaths between 25 February and 12 May 2020 giving a CFR of 5.0% (4)<br>• By 14 May, there were 758 recorded cases, 284 recovered cases and 42 deaths in Kenya (25), and by 28 May there were 1618 recorded cases, 421 recovered cases and 58 deaths giving a CFR of 3.6% (25)<br>• By 30 June, this had increased to 6366 reported cases, 2039 recovered cases and 148 recorded deaths giving a CFR of 2.3% (7, 25)
Lesotho	• Currently there are no laboratories testing for COVID-19 in Lesotho, with tests sent to South Africa for analysis if needed (no mass testing to date – 29 May 2020)<br>• By 12 May 2020, Lesotho had recorded its first case (4) with still only one recorded case by 19 May 2020 (5). This rose to 2 cases by 26 May 2020 and no deaths (6)<br>• However by 30 June, there were 27 reported cases but still no recorded deaths (7)
Malawi	As of 23 April 2020 (with the first cases reported on 3 April 2020 (26)):<br>• Number of tests performed: 553

	• Number of confirmed tests: 33<br>• Number recovered: 3<br>• Number of active cases: 27<br>• Number who are believed to have died as a result of the virus: 3<br><br>By 5 May 2020, there were 41 recorded cases and 3 reported deaths giving a CFR of 7.3% (2)<br><br>As of 9 May 2020 (27):<br>• Number of suspected cases and received samples: 1,188<br>• Number of tests performed: 1,231<br>• Number of confirmed tests: 56<br>• Number recovered: 14<br>• Number of active cases: 39 of which five are health workers<br>• Number who are believed to have died as a result of the virus: 3<br><br>• This increased to 58 recorded cases with still only 3 recorded deaths by 12 May 2020 giving a CFR of 5.2% (4)<br>• By 19 May, there were 71 recorded cases in Malawi with still only 3 recorded deaths giving a CFR of 4.2% (5), and by 26 May, there were 101 reported cases and only 4 recorded deaths giving a CFR of 4.0% (6)<br>• However by 30 June, there were 1265 reported cases and 16 recorded deaths giving a CFR of 1.3% (7)
Namibia	• By 14 April 2020, there were 16 recorded cases and no recorded deaths in Namibia (1). All cases were detected following quarantine after entry into Namibia at the Airport.<br>• The figures did not change by 5 May 2020, i.e. 16 recorded cases and no reported deaths (2). The numbers were the same on the 12 May (4)<br><br>As of 13 May 2020:<br>• Number of tests performed:1750 (NB: All are to be retested)<br>• Number of new cases: 0<br>• Number with active disease: 5<br>• Number of hospitals: 4 referral hospital & WCH<br><br>• By 19 May, there were still on 16 recorded cases in Namibia with still no recorded deaths (5), and by 26 May, there were 21 reported cases with still no recorded deaths (6)<br>• By 30 June, there were 203 reported cases but still no recorded deaths (7)
Nigeria	As of 23 April:<br>• Number of tests performed: 10061<br>• Number of positive tests: 981 (with 15 laboratory facilities in Nigeria)<br>• Number recovered: 197<br>• Number who have died as a result of the virus: 31 with a belief that the majority of deaths were outside established treatment centres<br><br>As of 3 May 2020 (28):<br>• Number of tests performed: 18536<br>• Number of positive tests: 2558 (69% males)<br>• Number recovered: 400<br>• Number who have died as a result of the virus: 87 (CFR = 3%)<br><br>• This increased to 4787 recorded cases with 158 recorded deaths between 25 February and 12 May 2020 giving a CFR of 3.3% (4), and by 19 May there were 6401 recorded cases in Nigeria with 192 recorded deaths giving a CFR of 3.0% (5)<br>• By 26 May, there were 8344 reported cases and 249 recorded deaths giving a CFR of 3.0% (6)

	• However by 30 June, there were 25694 reported cases and 590 recorded deaths giving a CFR of 2.3% (7)<br><br>Overall, the cases in Nigeria are predominantly mild to moderate and largely asymptomatic based on ongoing community surveys
Rwanda	• By 14 April 2020, there were 134 recorded cases and no recorded deaths in Rwanda (1)<br>• This increased to 261 recorded cases and still no recorded deaths by 5 May 2020 (2), and by 12 May, there were 286 recorded cases with still no recorded deaths (4)<br>• By 14 May, there were 287 recorded cases, 168 recovered cases and still no recorded deaths in Rwanda, and by 28 May there were 349 recorded cases, 245 recovered cases and still no recorded deaths (25)<br>• By 30 June, this had increased to 1025 reported cases, 447 recovered cases and only 2 recorded deaths (7, 25)
Senegal	• By14 April 2020, there were 299 recorded cases and 2 recorded deaths in Senegal giving a CFR of 0.7% (1)<br>• This increased to 1239 recorded cases and 11 recorded deaths by 5 May 2020 giving a CFR of 0.8% (2)<br>• By 12 May, there were 1995 recorded cases with 19 recorded deaths giving a CFR of 1.0% (4), and by 19 May, there were 2617 recorded cases and 30 recorded deaths in Senegal giving a CFR of 1.1% (5)<br>• By 26 May, there were 3161 reported cases and 37 recorded deaths giving a CFR of 1.2% (6)<br>• By 30 June, there were 6793 reported cases and 112 recorded deaths giving a CFR of 1.6% (7)
South Africa	As of 23 April 2020:<br>• Number of tests performed: 143,570 (89,501 in private and 54,069 in public)<br>• Number of positive tests: 9796<br>• Number in ICUs: 411 (hospital admissions)<br>• Number recovered: 1473<br>• Number died as a result of the virus: 75<br><br>• By late April there will be 9 national laboratories able to undertake testing in South Africa, with mobile laboratories bringing patient samples to these facilities; however, most tests are still being undertaken in the private sector (29)<br>• As of 5 May 2020, there were 7572 recorded cases and 148 recorded deaths giving a CFR of 2.0% (2)<br>• By 12 May 2020, there were 11,350 recorded cases with 206 recorded deaths giving a CDR of 1.8% (4), and by 19 May, there were 17200 recorded cases and 312 recorded deaths in South Africa giving a CFR of 1.8% (5)<br>• By 26 May, there were 24264 reported cases and 524 recorded deaths giving a CFR of 2.2% (6)<br>• However 30 June, there were 151209 reported cases and 2657 recorded deaths giving a CFR of 1.8% (7)
Sudan	• As of 23 April, Sudan has 2 operating laboratories and performed a total of 600 tests with162 positive cases, 14 recovered, 30 in ICU and 13 deaths<br>• By 12 May, there were 1687 reported cases and 78 deaths giving a CFR of 4.6% (14), and by 19 May this had risen to 2591 recorded cases and 105 recorded deaths giving a CFR of 4.1% (15)<br>• By 27 May, there were 4146 reported cases and 184 recorded deaths giving a CFR of 4.4% (16)<br>• By 30 June, there were 9258 reported cases and 572 recorded deaths giving a CFR of 6.8% (17)

	• There has been recognised under-reporting of cases in Sudan with only highly suspected cases with severe symptoms typically being tested because of limited resources for testing
Tanzania	• By 14 April 2020, there were 53 recorded cases and 3 recorded deaths in Tanzania giving a CFR of 5.7% (1)<br>• This increased to 480 recorded cases and 18 recorded deaths by 5 May 2020 giving a case fatality ratio of 3.8% (2)<br>• By 12 May there were 509 recorded cases with 21 recorded deaths in Tanzania giving a CFR of 4.1% (4), and on14 May, there were still only 509 recorded cases, 183 recovered cases and 21 deaths with the same rates reported on 21 May, 28 May and 30 June 2020 (7, 25)<br>• However, there are concerns regarding the appreciable under-reporting of both cases and deaths due to COVID-19 in Tanzania (30, 31)
Uganda	• The first case of COVID-19 was reported in Uganda on 21 March 2020 (32)<br>• Between 25 February and 5 May 2020, there were 97 recorded cases and currently no recorded deaths in Uganda (2)<br>• This increased to 126 recorded cases and still no recorded deaths by 12 May 2020 (4)<br>• By 14 May, there were 160 recorded cases, 63 recovered cases and still no recorded deaths, and by 28 May 2020 there were 317 recorded cases, 69 recovered cases and still no recoded deaths (25)<br>• By 30 June, this had increased to 889 reported cases, 817 recovered cases and still no recorded deaths (7, 25)
Zambia	• Zambia confirmed its first two cases of COVID-19 on 18 March 2020 (33), and by 14 April 2020, there were 45 recorded cases and 2 recorded deaths in Zambia giving a CFR of 4.4 % (1)<br>• This increased to 139 recorded cases and 4 recorded deaths by 5 May 2020 giving a CFR of 2.9% (2)<br>• As of 8 May 2020:<br>o There were 167 confirmed cases in total, 111 recoveries and 4 deaths giving a CFR of 2.4% (34). This was up from 74 confirmed cases, 35 discharged and 3 deaths on 22 April 2020 (35)<br>o 12095 test results processed at 3 designated laboratories in Zambia giving a 1.38% positivity rate (34)<br>• By 12 May 2020, there were 441 recorded cases with 7 recorded deaths giving a CFR of 1.6% (4), and by 19 May, there were 772 recorded cases and still only 7 recorded deaths in Zambia giving a CFR of 0.9% (5)<br>• By 26 May, there were 920 reported cases and still only 7 recorded deaths giving a CFR of 0.8% (6)<br>• By 30 June, there were 1594 reported cases and 24 recorded deaths giving a CFR of 1.5% (7)
Zimbabwe	• The first imported case of COVID-19 was reported on 21 March 2020, with local transmission starting on 24 March (36, 37)<br>• As of 23 April 2020, Zimbabwe had 6 testing laboratories (1 national and 5 at sub-national levels). A total of 5,450 tests had been conducted by 23 April with 28 positive cases, 2 recoveries/discharged and 4 deaths<br>• By 5 May 2020, there were 34 recorded cases and 4 recorded deaths giving a CFR of 12% (2)<br>• As of 7 May, there have been a total of 9,594 rapid screening tests and 8,141 PCR diagnostic tests, 34 recorded cases, 9 recoveries and 4 deaths (38)<br>• By 12 May 2020, there were 37 recorded cases with 4 recorded deaths giving a CFR of 10.8% (4), and by 19 May, there were 46 recorded cases and still only 4 recorded deaths in Zimbabwe giving a CFR of 8.7% (5)<br>• By 26 May, there were 56 reported cases with still only 4 recorded deaths giving a CFR of 7.1% (6)<br>• However 30 June, there were 591 reported cases and still only 7 recorded deaths giving a CFR of 1.8% (7)

NB: CFR = Case fatality ratio; ICU = Intensive care units

Table 2A – Ongoing activities regarding COVID-19 across Africa (**to be updated and in line with Table 2 and explaining extended lockdown where pertinent)**

Country	Activities and challenges to address COVID-19
Algeria	• March 17 - Algeria suspended all land borders as well as air and maritime links (39)<br>• March 23 - a full lockdown of the Wilayat of Blida and a partial lockdown of the capital Algiers (39)<br>• April 24 - the government took steps to ease lockdown restrictions by allowing several businesses to reopen. Businesses included those selling materials for building and public works, appliances, fabrics, clothing and shoes, home and office furniture, pastries and hairdressers as well as urban transport by taxis (40)<br>• The lockdown was eased as the restrictions had significantly hit the economy, which was already under appreciable financial pressure due to a sharp fall in global oil prices, and in view of the ongoing social impact. The situation is continually being monitored (40)<br>• June 7 – Lockdown restrictions further eased; however the curfew will remain in place and borders still closed (41)
Botswana	• March 16 – Social distancing measures imposed (42)<br>• March 18 – Closure of all learning institutions with effect from March 23 (42)<br>• March 19 - Funerals shall be brief and during burials, strict hand washing and surface cleaning is recommended for all equipment and to further minimise risk, no food service shall be provided to those in attendance (42)<br>• March 20 - Declaration of a state of public health emergency by the Director of Public Health, with the state of emergency endorsed for another 6 months in early April 2020 (42, 43)<br>• March 23 - Establishment of a high-level national response committee chaired by the President which has been meeting three times a week since March 23 and adjournment of parliament with members mandated to sensitize their constituencies to fight the pandemic (42). Measures include (42, 44):<br>o Strict border control including closure of some borders (from March 20) and screening for COVID-19 at other ports of entry, immediate suspension of issuance of visa for travellers from high-risk countries with citizens from China, Japan, South Korea, Iran, USA, UK, Austria, Belgium, Denmark, France, Germany, Italy, Netherlands, Norway, Spain, Sweden and Switzerland prohibited from entering Botswana<br>o Restrictions on local and international air travel and 14-day quarantine from those entering the country apart from truck drivers (from March 24)<br>o March 28 - All international and domestic commercial flights suspended (45, 46)<br>• April 2 – Lockdown measures introduced severely restricting movement with essential service employees needed permits from the government for their movements (47); subsequently lifted on May 22 (48)<br>• 21 laboratory scientists receiving WHO training on specimen collection and shipment across districts with six more currently being trained to enhance testing capabilities<br>• Establishment of 10 isolation and case management centres spread across the country<br>• May 1 - Mandatory use of surgical or home-made face masks in public places (46), with surgical masks for health care workers/frontline workers;

	N95 for those attending COVID-19 suspects with full PPE for those attending confirmed COVID-19 cases.<br>• To reduce misinformation on COVID-19, dissemination of information restricted to the Director of Public Health and the WHO, with defaulters liable to payment of fines or imprisonment or both<br>• Establishment of a COVID-19 relief fund to cushion the socio-economic impact of the pandemic (42)
Cameroon	• The government established coordination systems at all levels equipped with the necessary training and resources to undertake preventive measures and case management<br>• 18 March - Preventive measures included (9, 49, 50):<br>o Closure of borders<br>o Closure of schools, vocational training centres and professional schools<br>o Public to strictly observe hygiene measures including frequent hand washings, social distancing, avoiding touching eyes, nose and mouth, and respiratory hygiene<br>o Other measures include a ban on gatherings of more than 50 people and disinfection of markets and other public places and compulsory wearing of masks in public places (from 13 April)<br>• Restrictions were eased early May to help address economic and social hardships (51)<br>• Establishing a surveillance system with hot lines and integrated remote database (geotags and DHIS2) as well as case reporting, contact tracing mechanisms with a 72.19% success rate currently and case management with guidelines. In addition, establishing laboratories for PCR testing and scaling up from 3 initially to 10 currently<br>• Establishing specialized case isolation and treatment centres in all regional hospitals with plans to treat and feed patients at no cost.<br>• Community engagement through media including, radio, TV, and social media with special attention to the indigenous people, with an estimated 9 million people reached by 19th April 2020.<br>• The government promoting local initiatives including the production of medicines including chloroquine by the Ministry of Research enhanced by concerns with counterfeit medicines (52). In addition, household production of cloth masks and hand sanitizers.<br>• Partnership, technical support and donations from NGOs, development agencies, foreign governments, corporate bodies and religious institutions including PPE (53)
Democratic Republic of Congo	• The Republic noted its first case of COVID-19 on 10 March 2020, with the WHO providing testing kits to the National Institute for Biomedical Research in Kinshasa to help track the virus (8)<br>• 3 March – Quarantine measures introduced for travellers returning from high-risk countries (54)<br>• 24 March - A state of emergency was declared resulting in the closure of all borders to non-essential traffic and a ban on all trips between the capital and the country's 25 provinces, with the state of emergency subsequently extended (55). This builds on experiences with other infectious diseases in the Republic (56)<br>• 2 May – Lockdown measures extended to 15 May (57)<br>• 6 June – Curfew measures remain and travel (international and domestic) still suspended (58)<br>• Ongoing activities include (with the support of UNICEF) (59):<br>o 215 radio stations and 32 television channels have continued their COVID-19 awareness campaigns, particularly in the 5 most affected provinces with essential messages on prevention as well as other individual and collective protection measures<br>• Delivering infection, prevention and control supplies as well as Community Action Committee members raising the awareness of COVID-19 and

	talking about the importance of decontaminating premises and psychosocial support
Egypt	• Early commencement of testing of suspected cases, contact tracing, and case management at isolation hospitals as well as the availability of well-equipped ambulances for transporting confirmed cases. This was in recognition that early detection, testing and tracing in Egypt would help to control the infection and mortality, also helped by mandatory vaccination against TB with the BCG vaccine (60)<br>• The MOH has developed standard treatment protocols for COVID-19 confirmed cases in its hospitals as well as preparing fever hospitals (affiliated to MOH and distributed all over Egypt) and converting other hospitals to quarantine hospitals to diagnose and treat patients (61)<br>• Good coordination between public and private hospitals to increase the number of health care facilities available for isolation and treatment of patients with COVID-19<br>• Egyptian MOH through TV/Radio/social media/mobile apps (Sehat Misr) have increased public awareness about WHO-advised basic protective measures and encouraged the public to follow these. In addition, dedicated hot lines are available to citizens regarding COVID-19 related needs including seeking information and help<br>• 25 March - The government imposed a night time curfew and other lockdown measures (13, 62). In addition, all educational institutions have been closed with instigation of distant learning and the suspension of incoming commercial passenger flights, banning of all social, entertainments, sporting and religious gatherings with social distancing imposed. However, there are concerns especially among the poor and initially screening from international flights only applied to travellers from China (63, 64)<br>• The Armed Forces:<br>o Has helped with the availability of both face masks and sanitizers for all citizens as there were protective gear shortages initially as well has helping to sanitise State Institutions and other settings to slow down infection rates (65, 66)<br>o Established a field hospital at Cairo's International Exhibition Centre as well as other field hospitals with a total capacity of 4000 beds as well as providing 50 ICU ambulances and medical aircraft (67, 68)<br>• Egypt has also sent medicines and medical aids to other African countries to help them in their fight against the pandemic (69-71)
Eswatini (Swaziland)	• 30 January – First draft of the National Contingency Plan for Novel Coronavirus (COVID-19) issued with the help of the WHO and UNICEF (72)<br>• 11 March - Pandemic declared a State of Emergency, with the National Disaster Management Agency of 2006 used to declare a state of emergency leading to a partial lockdown in Eswatini. This included the institution of legislation, regulations and guidelines for disaster response<br>• 27 March - A partial lockdown directive was issued including: closure of borders, screening at borders and airports with mandatory 14-day quarantining for returning passengers (10 centres overall in Eswatini), domestic travel restrictions, public transport only for essential movement, and limited gatherings socially with security forces deployed to enforce the lockdown (73, 74). As a result of the lockdown many religious organisations are meeting via Zoom or teleconferencing<br>• 27 March - A person, an institution or organization that indulges in misinformation or provides details of the COVID-19 infection status of any person commits an offence and shall, on conviction, be liable to a fine not exceeding twenty thousand Emalangeni (E20,000.00) or imprisonment for a term not exceeding five years (75)

	• April - The lockdown was initially eased in April but re-instated on 24 April following the doubling of cases in a week (76, 77). This is likely to remain; however, there are concerns with enforcement in reality<br>• Contact tracing undertaken through Emergency Service and Regional Emergency Response Teams, with screening for COVID-19 symptoms for contacts of positive cases through the use of a commissioned regional rapid response teams as well as screening at entry points of hospitals and returning travellers. Initially, samples were sent to South Africa NICD laboratory for testing which caused challenges. However, the Country Laboratory started a verification process in March which was completed 17/04/2020 enabling in-country testing for COVID-19 to increase testing abilities. There is also increased capacity at the Mbabane Molecular Laboratory including the establishment of systems for seamless reporting of results<br>• All frontline healthcare workers should be tested for COVID-19 to ensure their protection for continued service delivery; however, there is currently a shortage of test kits although this is being addressed<br>• Lubombo Referral Hospital designated as a COVID-19 Management Facility for admitting confirmed cases and screening<br>• Distribution of PPE to all health facilities with supplies and equipment helped by donations from Jack Ma Foundation (78), with surgical masks and respirators initially reserved for health service personnel. The EU, Taiwan and others have also donated monies and supplies to Eswatini to help with the COVID-19 pandemic (79, 80)<br>• 22 April – Ministry of Health emphasized prevention management including hand washing and proper wearing of masks (81) with sanitizers available at entrances of supermarkets and businesses although concerns with availability. The University of Eswatini as well as a wholesale pharmaceutical company in Eswatini are manufacturing hand sanitiser to help reduce shortages<br>• Groups such as WHO and UNICEF are helping with the promotion of hygiene measures as well as providing washing supplies. UNICEF is also working with bilateral partners to bridge the gap in the provision of adequate WASH facilities at designated COVID-19 facilities. UNICEF in collaboration with MoH Environmental Health Department has provided 3 mobile showers and other wash support to help with hygiene at COVID-19 referral hospitals. The government is also planning to have another site for asymptomatic cases which will also require WASH support<br>• UNICEF also supports sensitization of adolescents on prevention of HIV and COVID-19. In partnership with Super Buddies, MoH Sexual and Reproductive Health and Save the Children supports Community Adolescent Treatment Supporters to provide information online through WhatsApp groups and Facebook.<br>• In addition, the Government has called for commuter omnibuses to operate with reduced number of passengers and reduced number of omnibuses on specific routes with passengers needing a permit to travel. There is also now a law that everyone boarding public transport must wear a mask with a press statement against sharing of masks. Travel on roads is also restricted, typically truck drivers transporting essential products<br>• 6 May – Further emphasising preventative measures (82)
Ethiopia	• Ethiopia instigated a state of emergency early to try and curb the spread of COVID-19 including closing schools and borders, banning public gatherings and events, requiring most employees to work from home and spraying the main streets in the capital with disinfectant (83-85). Key dates include (86):<br>○ 16 March - 10-point measure plan including banning large gatherings, closing educational institutions with the exception of higher learning institutions, limiting gatherings in religious

	institutions and launching a national hygiene and preventive measure movement<br>o 20 March – banning flights to more than 30 countries<br>o 22 March – closing land borders<br>o 1 April – The Northern Tigray region has become the first regional state in Ethiopia to have a COVID-19 testing lab (this alsong with initiatives to increase the level of testing from a previous low base (83)<br>o 3 April - Jack Ma donation of PPE distributed<br>o 23 April – Northern Tigray relaxes its state of emergency<br>• The Government is also working on providing protective face masks, medical kits and disinfectants generally for its citizens (84)<br>• This builds on initiatives that were introduced in 2018 to help prevent the spread of Ebola from the Democratic Republic of Congo where 39 cases of were reported between April 4 and May 13 and 19 deaths (87)
Ghana	There have been multiple activities in Ghana to try and reduce the impact of COVID-19. These include:<br>• 16 March – Restrictions on entry into Ghana, 14-day quarantining for those entering, suspension of public gatherings, closure of schools, private burials restricted to 25 people with 1-metre social distancing (88)<br>• 22 March – Closure of all borders (89)<br>• 7 April - Five local manufacturing companies selected to immediately start producing nose masks and other forms of PPE to help fight the pandemic given current shortages (90, 91)<br>• Early April - wearing of face masks compulsory in public places in the Greater Accra region and the Cape Coast Metropolis, as well as in other places where difficult to social distance<br>• 21 April – FDA publishes recommendations for home-made masks (92)<br>• 15 June – Mandatory wearing of masks in public places due to a a surge in COVID-19 cases in recent weeks (93)<br><br>In adddition:<br>• The population are also being generally educated about hand washing, which is seen as effective, as well as avoiding touching eyes, noses and mouths where possible. In addition, the populace has been advised to seek medical care early where perceived symptoms - seen as easier after the lockdown as any suspected case can be tested with assistance at health centres and confirmed cases rapidly transferred to treatment centres. Alongside this, enhancing testing abilities especially outside of major cities in the country with the help of drones (21, 94)<br>• Religious organisations have offered isolation units for quarantined cases, with private companies offering help including PPE and test kits and the US Army helping to build clinics (95)<br>• Two hospitals in the Greater Accra Region; Ga East Municipal and Bank of Ghana Hospitals have been fully dedicated as COVID-19 treatment centres, whilst other major hospitals across the country have been identified as centres to support the management of this disease<br>• To help with the management of severe patients; Ghana initially had over 100 ventilators in the health system as of January 2020. Subsequently, 307 ambulances were procured for the National Ambulance Service (NAS) equipped with mobile ventilators, with arrangements for the procurement of 50 more ventilators (96)<br>• The Food and Drugs Authority (FDA) in Ghana has also fast-tracked 24-hour testing and approval of alcohol (>70 %) based hand sanitizers to mitigate the shortage of imported products
Kenya	• The Ministry of Health taskforce to deal with COVID-19 launched and co-ordinating county efforts in combating COVID-19 with early initiatives including (97):

	○ Enhanced surveillance at all points of entry, health facilities, and communities<br>○ Increasing diagnostic capacity established at National Influenza Centre laboratory and other network of laboratories which include Kenya Medical Research Institute laboratories. Currently, there are 15 Government approved testing laboratories including 7 in public hospitals, 4 in Private hospitals, and 2 Government Agencies<br>○ Establishing coordination committees at both policy and technical levels<br>○ Ongoing training of health care staff on prevention, early detection, management of suspected/confirmed cases<br>○ Advising against non-essential travel<br><br>Key dates include:<br>• 13 March - suspension of public gatherings, meetings, religious events and games, Subsequently, suspending travel in and out of Kenya, banning religious and social gatherings as well as imposing a nationwide curfew between 7pm and 5am (27 March) which has been enforced (98-101)<br>• 19 March – lockdown measures prohibiting movement in and out of Nairobi and Mombasa cities, closure of markets, hotels and social places, with additional lockdown measures in the residential areas identified as hotspots; only essential services are being provided (102, 103), with further bans introduced 6 April (104)<br>• 22 March – Mass testing for International flights and 14-day quarantine for all those entering Kenya (105), with quarantine for 14 days (at own cost) for returning travellers<br>• 25 March – closure of land border with Uganda as well as suspending international flights (105)<br>• 5 April – Extending the cancellation of all incoming international passenger flights to at least May (105)<br>• 8 May - The government has taken on the responsibility of meeting all costs of quarantine and improvement of quarantine facilities to encourage citizens to get tested<br><br>In addition:<br>• Daily national briefings by MOH on COVID-19 status (new cases, recoveries and deaths) and the re-enforcement of containment strategies to keep the public informed and sensitized.<br>• The wearing of face masks has now become compulsory and people face arrest for not wearing one (106), alongside increased public education on the need for frequent handwashing, sanitizing, social distancing and wearing masks<br>• The government has also increased the procurement of PPE for healthcare workers, with enhanced local production of masks and other PPE as well as sanitizers to address shortages with the quality being checked by the Pharmacy and Poisons Board (PPB) and Kenya Bureau of Standards (KEBS) (107-109)<br>• There have also been donations of medical supplies including testing kits from the Jack Ma foundation (110) with additional support from the World Bank to address current shortages (111). The Equity Bank has also donated PPEs for health workers country wide<br>• The production of local testing kits and vaccine development by KEMRI is also underway to address concerns (112)<br>• The government is also currently increasing community testing and is encouraging citizens to voluntarily get tested, with ongoing efforts to produce testing kits locally. Students have developed an App for contact tracing as well as improving triage and management of COVID-19 patients (113) and Telkos Safaricom has donated thermal thermometers worth USD100,000 to support screening capability

	• Ventilators have also been developed and built locally to address shortages (114-116). In addition, local artisans have developed 'hands free' hand washing machines to prevent contamination during and after washing (117)<br>• Expansion of hospitals and creation of new infectious disease units to cater for increasing numbers of COVID-19 cases. This includes the Government working with schools, colleges, universities, and private guest houses and hotels to make isolation centres available all over the country.<br>• The Government has empowered all the three level six hospitals in the country to deal with moderate to severe cases and designated three special treatment centres for COVID-19 patients in Nairobi and at each of the County Hospitals across the 47 counties in Kenya<br>• There has also been the development and revision of treatment guidelines for managing COVID-19 patients, case definition, guidelines for screening and detection of COVID-19, guidelines for HIV management during COVID 19 as well as guidelines for Reproductive and Family Planning Care against the background of COVID-19 (118). Guidelines for physiotherapy support, Psychological support and home-based care have also been developed<br>• Kenyatta National Hospital and University of Nairobi have organized and coordinated daily COVID-19 related webinars on topical areas of concern with experts and health institutions sharing the latest information and updates to improve national responses. International experts from major COVID-19 affected countries have also participated in the webinars harnessing learnings from global experiences<br>• A number of local Banks, philanthropists and professional groups have made significant monetary and material support towards COVID-19 containment and social support.<br>• Efforts to employ more healthcare workers (8000) and to provide COVID-19 monthly allowances to motivate currently employed healthcare workers<br>• Local electronic and print media, volunteers and other groups addressing misinformation about COVID-19 and possible treatments (98)
Lesotho	• The national response command centre in Lesotho provides guidance on all matters concerning COVID-19<br>• Key dates include:<br>○ 30 March - Lockdown measures were introduced (119). Concerns though that hygiene measures are difficult for many due to the scarcity of water and poor access to sanitizers. Lockdown measures are now being eased (5 May) due to socioeconomic challenges although still regulations on wearing masks and enforcing business owners to ensure patient safety through social distancing and hand sanitisation as ongoing concerns (120, 121)<br>○ 30 March – Borders with South Africa closed (119)<br>• The government through the District Healthcare Teams (DHMT) supports healthcare facilities through capacity development and commodities including masks, sanitizers, and soap/ disinfectants and seeks to ensure functional taps for washing hands in all healthcare institutions combined with education on hand washing techniques. People are also being educated on how to cover their mouth and nose when coughing and to wash their hands after being in contact with respiratory secretions<br>• Local and international private organisations and NGO's have provided support to the MOH with PPE and testing equipment, as well as supported educational programs on COVID-19. NGOs have also helped provide online educational programs as well as supplies of sanitizers, masks and soap<br>• Religious organisation and private business owners have been providing food parcels to support the poor and the vulnerable as well as purchasing laboratory equipment and providing financial support to the government (private companies)

	• There are still challenges with supplemental oxygen supply in Lesotho as well as insufficient ventilation apparatus (both invasive and non-invasive)
Malawi	Ongoing measures to reduce the number of COVID-19 cases with concerns with limited availability of healthcare facilities include:<br>• 7 March – Special Cabinet Committee on COVID-19 started operating to co-ordinate activities (122)<br>• 20 March – COVID-19 was declared a National Disaster with the introduction of a Cabinet Committee on Coronavirus, which was later restructured into Presidential Taskforce on Coronavirus (123)<br>• 21 March – Banning of public gatherings including religious gatherings and wedding ceremonies (122)<br>• 23 March – Closure of schools and colleges (122)<br>• 1 April - Suspension of international flights (122, 124) and travellers put under follow-up procedures to reduce potential transmission (125)<br>• 18 April - A national lockdown was due to come into effect – but suspended before being effected due to a court challenge by a human rights organisation (126-128)<br>• Introduction of COVID-19 workplace guidelines that included the introduction of work shifts and reducing the number of people going to work by telling all staff not providing essential services to operate from home (129)<br>• Strengthening of surveillance in all border posts as well as increasing the number of testing facilities to 5 in all regions throughout the country. The targeted number of testing facilities across the country is 15, achieved by May 2020 following activities to increase the number of facilities (125)<br>• By 29 April 2020, institutional isolation centres and treatment centres in Blantyre (Southern region), Lilongwe (central region) and Mzuzu (northern region) had been opened<br>• Patients with mild symptoms treated at their home. However, there are plans to start taking COVID-19 patients to specific treatment centres, with an ongoing strategy of trace, test and treat<br>• Recruiting new healthcare workers, aiming to recruit 2000 new health care providers (HCPs) (comprising mostly nurses and clinical officers) as well as 1,500 health surveillance assistants (HSAs) to intensify hygiene in the communities<br>• Acquisition of more Personal Protective Equipment (PPEs) for HCPs and HSA
Namibia	• All public health hospitals in Namibia have established isolation units for holding patients with positive testing for COVID-19, with hospitals currently having sufficient PPE to protect their health care workers<br>• All public health hospitals are also still in a position to manage mild/moderate cases of COVID-19 with four intermediate referral hospitals and the National referral hospital equipped with ventilators to manage severe cases of COVID-19<br>• Key dates include:<br>o 14 March – Banning of all large gatherings (130)<br>o 15 March – School closures and suspending International flights (130)<br>o 24 March – All foreign travellers banned from entering Namibia (131)<br>o 27 March - Lockdown restrictions were introduced in Namibia to help contain the spread of the virus with the public urged to remain at home and avoid public gatherings with enforcement if needed (132), and extended mid April until early May (133)<br>• From May 5, there has been some easing of restrictions. These include:<br>o Allowing travel between the different Regions in Namibia and within the cities and towns, with several businesses allowed to re-open including shopping malls, retail stores, hair dressers and

	restaurants but all subject to strict health and hygiene measures. All individuals will be expected to wear masks in public (134)<br>o Foreign nationals will continue to be banned from entry, with returning Namibian citizens quarantined for 14 days (134)<br>• Restrictions are still in force against large gatherings. As such, no students are allowed at education institutions; church gatherings are prohibited; and any social gatherings for entertainment or other purposes remain prohibited
Nigeria	• The Nigerian Centre for Disease Control (NCDC) took the lead by activating the National Emergency Operating Centre (EOC). This included protocols for case definitions, diagnosis and testing, contact tracing/surveillance and case management, self-isolation, rational use of PPE, and establishment of laboratories nationwide (135)<br>• The NCDC with other partners including UN agencies like WHO, UNICEF as well as Nigerian media organisations have sought to educating the public on COVID-19 and the implications<br>• The President of Nigeria inaugurated a multi-ministerial and multi-agency Presidential Task Force headed by the Secretary to The Government of the Federation with membership including the ministers of health, information, internal affairs, foreign affairs, education and the Director-General of the NCDC among others to coordinate activities (136). These include closure of borders, a ban on all international and local air flights (effective from 23 March 2020); lockdowns; closure of all schools; instigation of preventive measures including social distancing/ ban on social and religious gathering and hand sanitizing with alcohol-based sanitizer. In addition, distribution of relief packages in the form of cash and food items to those identified as 'most vulnerable' due to hunger and poverty<br>• There are concerns though that many Nigerians do not have access to water and basic sanitation to readily instigate required hygiene measures (137)<br>• 30 March – Cessation of all non-essential movements in Lagos and FCT, all citizens to stay at home and travel to and from other states postponed, all air travel suspended (137, 138)<br>• Alongside this, State governors have taken a leadership as the coordinators of the response activities in their States, with some States initially making the wearing of face masks mandatory in public places (26 April) (139), with the policy now adopted nationally with penalties for violations. However, there are concerns that face masks are not been worn correctly (137, 140)<br>• Faith based organisations have also responded by educating their members on the need for compliance with measures instituted by the federal and state governments as well as donations of food and other items<br>• The EU has also donated €50million to Nigeria to help tackle the virus (141)<br>• The NCDC and others also issue regular statements and reports to help address misinformation<br>• The National Agency for Food and Drug Administration and Control (NAFDAC) has also reported concerns with falsified chloroquine (142)
Rwanda	• The limited deaths in Rwanda (2 deaths up to up end June) may have been helped by messages of frequent hand-washing and staying at home, with unnecessary movement and visits outside the home prohibited except for essential services with automated screening also available from Mid March onwards (7, 143-145)<br>• In addition, the speed with identifying potential cases and their admission to hospital<br>• 19 April - Wearing of masks also become compulsory early in the pandemic, helped by local production (144)<br>• End April - 29,395 citizens had been tested for COVID-19 giving a prevalence of 0.7% (145)

	• Drones are being used for street control/messaging and transporting medicines to patients including those for cancer patients (146), building on their existing use to transport blood products and medicines to hospitals and patients in remote regions (147, 148). Telephones have also been instrumental not just for spreading information, but also communication between people during lockdown to ease the burden<br>• Robots are also being used in hospitals<br>• 4 May - some easing of lockdown measures including allowing some people to return to work and markets open for essential vendors (149); however re-instated in some localities in late June with a rise in new cases (150)
South Africa	• Joint command centres were established at the National, Provincial, and District levels chaired by the President, Premiers and Mayors respectively. The teams comprised of experts from government, private companies, academic and non-governmental sectors in line with WHO operational guidance (151, 152). These structures also included experts from international organizations such as WHO and CDC-USA<br>• South Africa declared a national state of disaster in terms of Section 3 of the Disaster Management Act (Act no 57 of 2002) (153). Subsequently, Stage 5 Lock down was introduced on 26/03/2020, which included a complete travel ban, was eased to Level 4 with effect from 04/05/2020 (154-156) [Section 1: Normal activity can resume with precautions and health guidelines followed at all times; Section 3: Easing of some restrictions including work and social activities; Section 4: Some activity allowed subject to extreme precautions to limit community transmission and outbreaks; Section 5: Drastic measures are required to contain the spread of the virus including lock downs, travel bans and social distancing (157, 158)]<br>• 27 March - Lockdown measures introduced (155, 159), and included the closure of educational establishments and leisure activities, maintaining social distancing enforced in shopping malls and other complexes where there are controls on the number of people allowed in facilities at any one time and a reduction in public transport, large gatherings prohibited, and wearing of face masks in public. In addition, travel bans with enforcement through interdepartmental collaboration (152, 155, 159, 160). However, it is acknowledged that such measures are difficult to implement in townships (161)<br>• April 2020 - Mass-screening was introduced, with all patients regularly screened at hospitals, with health care workers screened and tested daily if required. In addition, nursing personnel at the PHCs have been trained to collect samples for testing for COVID-19. South Africa uses RCT-PCR technique for diagnosis, with all symptomatic cases being tested, undertaken in both public and private centres in each of the 52 districts<br>• Early April 2020 - Standardisation of PPE use in healthcare facilities, with the use of facial masks/ facial coverage and hand sanitizers regulated (162). The push system has been used to address shortages with PPE with public awareness that PPE should be made available to healthcare professionals first. Public institutions have also conducted facility assessments on the number of oxygen supply points and the number of ventilators to help prepare for patient influxes<br>• There have also been mass education by civic organisations and others regarding the signs and symptoms of COVID-19 and the benefits of lockdown coupled with national free hotlines and WhatsApp numbers to obtain information as well as the development of self-check Apps to guide patients on what to do if they suspect infection (10 April) (163)<br>• Isolation centres were established in each of the 9 provinces for COVID-19 patients who cannot self-isolate. In some Provinces, e.g. Gauteng, dedicated hospitals have been created including Steve Biko Academic Hospital in the Tshwane District

	• A multidisciplinary team at Wits University has also used their design and engineering skills to create face shields to help address current shortages (164). This is in addition to local firms designing and building ventilators to address shortages (165). South Africa is also repurposing laboratories from researching HIV into potential treatment approaches for COVID-19 (166)<br>• There were concerns with a shortage of ICU beds in South Africa; however, this was managed in line with ICU triage and rationing guidelines (167, 168) by increasing the number of ICU beds in the hospitals (169)<br>• There are also hygiene measures in place, e.g. among health facilities the number of taps has been increased at the main gates and other strategic positions with detergent available for the public to use before entering healthcare premises<br>• Since the start of the lockdown, 28,000 community health care workers have also been mobilised for active case-finding in vulnerable communities through door-to-door screening and testing to help limit the spread of the virus (170)<br>• Such measures could expand with the potential for 45,000 South Africans dying from COVID-19, down from earlier estimates of 120,000 and 150,000 (171). However, there are ongoing concerns that if the lockdown is relaxed too early this will lead to exponential increases in the number of cases and mortality (159)<br>• The South African government has made the spreading of misinformation concerning COVID-19 an offence punishable by a fine, six months' imprisonment, or both (172). In addition, the South African Pharmacy Council have warned against the misuse of hydroxychloroquine, azithromycin and antiretroviral medicines for the treatment of COVID-19 building on concerns from the regulatory agency given the lack of evidence (173, 174)<br>• The South African government has also amended its medicine and related substances Act to extent the period of repeat prescription from 6 months to 12 months for schedule 2-4 drugs to minimise hospital visits and reduce patients exposure to COVID-19 (175)
Sudan	• The Sudan is seen as a country with a relatively high vulnerability for importation of COVID 19 (3); however, with currently poor resources for quarantine facilities for suspected cases at airports and hospitals, and weak tracing capacity to contact suspected cases community transmission could be easily established<br>• To address this, the Federal Government inaugurated a National Ministerial Committee with the MoH leading the planning and implementation of the response activities. These include (176-178):<br>o Early/ Mid March – Schools, airports and borders closed as well as restaurants and cafes<br>o Mid March – Pharmacists asking citizens not to discard empty hand sanitizer bottles as they will refill them free of charge<br>o 30 March – Inter-state public transportation halted and a country wide curfew between 18:00 and 06:00<br>o 18 April – Further lockdown measures introduced<br>o Ongoing – closure of schools<br><br>• However ongoing concerns include (176-178):<br>o Extremely limited access to safe water and sanitation for an appreciable number of citizens and poor hygiene practices by many will further increase risk of transmission<br>o Testing (PCR) is currently limited – mainly suspected cases with severe symptoms because of limited resources for testing, which are currently only available in two facilities in the country<br>o Only the isolation and treatment centres in the capital, Khartoum, are relatively well equipped (having ventilators and oxygen supply), others at the state levels have variable capacities and equipment

	◦ Prices of facemasks have increased ten fold in Khartoum (by 24 March)<br>◦ The country response plan to COVID-19 launched in May 2020 also discussed raising awareness (social distancing, hand washing, use of masks, hand sanitizers) using direct contact and mass media; screening at point of entry; home isolation with regular follow-up; testing (PCR) of suspected cases; isolation of confirmed and suspected cases; travel bans; partial lockdown; and strengthening (training, equipping) of the health system (178)<br><br>• Other activities include:<br>• International agencies such as WHO and UNICEF provide technical support to MOH. They also support with testing materials, surgical masks and others. Sudan FETP (field epidemiology training programme) and graduates are involved in all these activities at all levels of the health system<br>• There is an adopted policy for the use of masks and other protective facial coverage principally for health personnel; however, the ability to cover all health personnel is currently hampered by lack of resources
Tanzania	• Tanzania appears to have taken a different approach to COVID-19 compared with other African countries in the belief that stringent social isolation measures would severely damage the economy (31, 179). This appears contrary to activities of the other East African nations contained in their strategy document (180)<br>• Large public gatherings have been banned and schools and universities closed; however, markets and places of worship remain open (31)<br>• More recently (12 May 2020), the WHO in collaboration with the Ministry of Health is converting a commercial site in the capital city into a 500-bed capacity COVID-19 treatment centre to help cope with the pandemic (14)
Uganda	• Uganda has implemented a considerable number of measures to help limit the impact of COVID-19 building on previous experiences (181)<br>• 20 March - Lockdown measures initiated even before the first case was registered, and extended further until May although slowly releasing Mid May onwards (32, 182-185). Measures included: closure of educational Institutions; suspension of communal prayers as well as discos, sports and cinemas; stopping all public political rallies, cultural gatherings or conferences; closing borders and banning international travel to infected countries (although cargo transport must continue but with the minimum number of people); mandatory quarantine for 14 days (at own cost) for returning travellers; maximum number of people allowed at e.g. weddings, burials and other gatherings; suspending weekly or monthly markets such as cattle auction markets (ebikomera) but non-agricultural gatherings at e.g. factories, financial institutions, media, and private Security companies allowed if Standard Operating Procedures are followed although all non-food shops closed and stopping of public transport although private vehicles allowed but up to a maximum of three people (not taxis). Crucial employees should be allowed to camp around the factory area to prevent infection spreading. Construction sites should also continue if they can encamp their workers. Super-markets should remain open but with clear SOPs that restrict numbers that enter and leave the site at a given time and the handling of trolleys within the super-markets, with home deliveries encouraged<br>• Alongside this - advising the public to maintain hygiene measures such as regular washing with soap and water or using sanitizers and disinfecting surfaces as well as good nutrition and other measures to strengthen the body’s immune system (186)<br>• In addition:<br>◦ Regular presidential addresses to the nation with consultation with health experts at the MoH to give an update of COVID-19 and

	provide guidance with information Communication and educational material developed by the MoH disseminated through electronic and print media. Media campaigns using prominent people to raise awareness about COVID-19<br>o Task forces established at national, regional and district levels to coordinate the response and provide guidelines for the prevention, case management and surveillance of patients, with a multi sectoral approach used to coordinate and enforce guidelines<br>o Joint testing and observing testing protocols for truck drivers crossing into Uganda from the East African Region – End May (187). In addition, the adoption of a mobile application known as "Truck Drivers Journey Management System", to monitor the drivers throughout their journey as well as seek Uganda drivers for trucks entering Uganda (188)<br>o Mandatory wearing of masks when in public in addition to further guidance for truck drivers (21 May) (189)<br>o Food distribution to 1.5 million urban poor affected by the lockdown (190)
Zambia	• The Zambia National Public Health Institute (ZNPHI) triggered a national COVID-19 response following the outbreak in China and other countries, with the outbreak formally declared in Zambia on 8 March 2020 (35). Zambia is largely relying on disease surveillance, infection prevention, health promotion, case detection and management, and enhanced human resource capital development as key strategies to prevent COVID-19 spread locally (191).<br>• Initial responses included (starting Mid March) (35, 191, 192):<br>o Instituting mandatory 14-day quarantine for travellers entering Zambia, regulation of COVID-19 infected areas, establishment and conversion of facilities into isolation centres, restricting social gatherings, limiting non-essential travel within and outside geographical regions with confirmed cases, as well as regulations regarding businesses and vending to include social distancing and hygiene measures. Wearing of face masks was made mandatory in all public places. More recent measures include temporarily closing the border with Tanzania on May 11 following a spike in cases and to enable considerable screening and testing in the Boarder town (193)<br>o Where COVID-19 cases have been detected, mass screening and testing has been enhanced by ZNPHI with now 3 designated testing centres (4245 samples received by 22 April)<br>o Sensitisation of the population through electronic and print media on preventive measures to limit the spread of COVID-19. Messages focusing on encouraging people to stay home, observe social distancing, practice hand hygiene and wear face masks in public places. This has been aided by a Transaid team working in rural Zambia to ensure communities have critical information about COVID-19 (194)<br>o Active procurement of disinfectants and PPE including gloves, face masks, aprons and hand hygiene supplies. Use of PPE has been enhanced in the quarantine and treatment centres<br>o Closure of all educational institutions and consideration of e-learning platforms, with a planned reduction of working hours and encouraging employers to ensure non-essential workers work from home<br>• Alongside this, first and second level hospitals continuing to provide routine primary healthcare and prevention health services along with some faith-based organisations and other facilities re-furbished and used as isolation centres

	• Donation of COVID-19 rapid test kits, assorted PPE, hand sanitisation equipment and hospital equipment by local companies, NGOs and other organisations (195, 196)<br>• Ministry of Health training and recruiting front-line health workers to enhance case management, surveillance and infection, prevention and control measures
Zimbabwe	• On the 17 March, the Government in Zimbabwe declared COVID-19 a national disaster and 31 March 2020, the Government of Zimbabwe launched the National COVID 19 Preparedness and Response Plan involving an Inter-ministerial Taskforce with 9 pillars of the response each headed by a pillar lead (197, 198). The pillars are: Country level Coordination, Planning and Monitoring, Points of Entry (POE), National Laboratories, Case Management, Infection Prevention and Control (IPC), Surveillance, Rapid Response Teams (RRTs) and Case Investigation, Risk Communication and Community Engagement, Operational support and Logistics and Law enforcement and security.<br>• Activities included:<br>o Declaration of a state of national disaster and bans on gatherings of more than 100 people (including church gatherings)<br>o Closure of all borders allowing only Zimbabwe nationals and residents to enter the country. All returnees have to be quarantined for 21 days post-arrival.<br>o Closure of all schools ahead of normal end of terms/session<br>o National lockdown permitting movements for only essential services (healthcare, food, security, fuel stations and supermarkets) on 30 March (decreed on 27th March to take effect on 30th March) - now further extended by 14 days from 1 May 2020 (199, 200)<br>o Establishment of COVID-19 treatment centres at one of the two infectious diseases hospitals in Harare and another one in Bulawayo. In addition, ongoing transformation of existing national, provincial, district and local health facilities to accommodate COVID 19 related cases. Alongside this, support from religious organisations and the private sectors for renovation of designated treatment centres as well as technical support from NGOs and international agencies including the WHO<br>• National guidelines have been updated to include COVID-19 and its management<br>• Local production of PPE and hand sanitizer upscaled with production of other products like disinfectants, liquid soap, vitamin C and paracetamol syrup along with support from relief agencies to enhance supply and distribution of PPE (200, 201)<br>• The measures appear to be effective in reducing transmission with testing upscaled from a low base (197). However, there are acknowledged challenges with the shortage of water supply in public places and homes and ineffective social distancing especially where relief materials and food are being distributed<br>• There has been considerable misinformation and false health advice circulating on social media in Zimbabwe. A law was now been enacted to deter fake news with up to 20 years in prison if found guilty (202)<br>• The Government outlined its plans for economic support for industries and individuals affected by COIVD-19 through its Economic Recovery and Stimulus Package (Table 3)

NB: MOH = Ministry of Health; PPE = Personal Protective Equipment

Table 3A – Shortages of medicines and other items and ongoing activities to address these

Country	Medicine and other shortages across Africa following COVID-19
Botswana	• No shortages of medicines have been recorded to date (29 May 2020)<br>• There have also been no reports to date of counterfeit medicines
Eswatini (Swaziland)	• There have been challenges with shortages of medicines for NCDs especially for hypertension with currently no production facilities in Eswatini (rely on importation principally from South Africa). However, the Ministry of Health has ensured the procurement of all insulins on the Essential Medicine List, and they are available at all levels of care during the pandemic<br>• In retail pharmacies, many medicines have been out of circulation for a period of time including ascorbic acid, chloroquine, ivermectin and immune boosters. The influenza vaccine has also been out of stock in retail pharmacies from 23rd April<br>• The Ministry of Health via the Directorate of Pharmaceutical services is sensitizing physicians, patients and pharmacists on the issue of fake medicines including chloroquine to address concerns
Ghana	• Currently approximately 70% of essential medicines are imported principally from China and India, with currently 15 local pharmaceutical manufacturing companies accounting for the remainder. The intention is to increase local production of essential medicines to 70% within three years (203)<br>• The closure of borders has limited the availability of APIs which could result in future shortages of essential medicines<br>• Active communication with the public regarding falsified chloroquine following endorsement of its potential for the treatment of patients with COVID-19 (204)<br>• Endorsement of hydroxychloroquine has resulted in shortages among some community pharmacies. There have also been shortages of vitamins and immune boosters in some pharmacies
Egypt	• There have been no shortages of medicines reported to date (29 May 2020) in Egypt<br>• There are approximately 120 pharmaceutical companies in Egypt (less than 10 multinationals with local production bases) with local supply covering approximately 90% of local demand (35% by local players and 65% by multinational companies) enhanced by Government activities to increase local production of medicines (205)
Kenya	• Currently there appears to be no shortages of essential medicines in Kenya helped by local manufacturing (206)<br>• However, there are concerns with local manufacturers in Kenya reporting there are issues with for instance the Indian pharmaceutical supply chain with India currently supplying 37% of Kenya's finished pharmaceuticals by value and a high proportion of sales volume (207); with the situation being monitored<br>• Overall there are 43 local manufacturers supplying 28% of local medicine requirement is met by local manufacturing – with principally anti-infectives (40% of local manufacturing) produced (208)
Lesotho	Currently, the supply chain of all medicines and general hospital commodities has remained intact with no drug shortages reported to date. However, the situation is being monitored
Malawi	• There has been no notable effect on the medicine supply chain to date in Malawi to date (29 may 2020); however, this may change with government attention currently diverted towards COVID-19 rather than other priority disease areas<br>• Having said this, there has been a scramble among citizens to purchase chloroquine and azithromycin as a result of reports that they are being used for the management of COVID-19 patients

Nigeria	• There are concerns with shortages of some essential medicines for diabetes, hypertension and malaria due to a sustained break in the supply chain, exacerbated by current import bans on goods from China and India (currently account for 70% of drug use). This will lead to real pressures within the next 3 months (August 2020) or earlier if not addressed (209, 210)<br>• There are currently 115 registered pharmaceutical manufacturing companies in Nigeria supplying 30% of current medicine need including analgesics, antimalarials, antibiotics, antiretrovirals, antacids, antihistamines, haematinics, cough and cold remedies, anti-ulcer medicines, antihypertensives and antidiabetics<br>• To help with this, a N100 billion (over US$256 million) intervention fund in healthcare loans has been set aside by the federal government intended to be made available to pharmaceutical companies and healthcare practitioners intending to expand/build capacity. In addition, the identification of a few key local pharmaceutical companies granted funding to support the procurement of raw materials and equipment to boost local drug production
South Africa	• Currently, there appear to be no major concerns with medicine shortages helped by local production; however, this is likely to change as the pandemic continues address this. There are concerns with the availability of medicines to treat patients with mental health disorders exacerbated by the lack of API with ongoing steps to try and address this (211, 212)<br>• There are an appreciable number of pharmaceutical manufacturing companies in South Africa with more than 50 currently listed in the Johannesburg Stock Exchange, with a significant portion of raw materials also manufactured locally, although South Africa also imports from other countries including India and China<br>• All medicines supplied in South Africa must be approved by the South African Health Product Regulatory Authority, reducing the availability of sub-standard medicine
Sudan	• Medicines are principally imported from China and India mainly with Sudan already experiencing medicine and medical supply shortages prior to COVID-19 outbreak as a result of the economic and political crisis, e.g. there was a 20% decrease in Sudan's medicine imports in 2019 compared to 2017 (213). Shortages have continued to worsen in 2020<br>• To help address this, drug shortages in Sudan are mainly handled by the National Medical Supplies Fund (NMSF), which monitors the situation and is in daily contact with importers and local manufacturers to help address the situation especially for medicines on the national essential medicines list.<br>• The NMSF approaches drug shortages approached through VEN/ WHO analysis technique according to their potential health impact of individual medicines (214)<br>• Overall, there are 25 registered pharmaceutical manufacturing companies in Sudan with functional capacity ranging from 40% to 85% meeting 38% of the demand for essential medicines.<br>• Currently, no current reports about circulation of such medicines in Sudanese market despite earlier concerns (215)
Uganda	• Medicines are sourced by Uganda National Medical Stores with for instance hydroxychloroquine (HCQ) sourced from India<br>• The Ugandan President has announced plans to begin to manufacture HCQ with raw materials from India – building on agreed strategies with the East African Community to increase local manufacture of supplies and pharmaceuticals (180)
Zambia	• Zambia has put in place a robust national strategic plan to help identify essential medicines shortages or potential shortages using the national electronic Logistics Management Information System (eLMIS) at all levels of the pharmaceutical supply chain (216)

	• In addition, Zambia has an established Essential Medicines List (EML) and Standard Treatment Protocols (STGs) for health care workers to refer to with alternatives are stipulated (217)<br>• Despite these initiatives, Zambia is currently undersupplied with essential medicines and dependent on imports of pharmaceutical supplies, e.g. there is currently there is a lack of supply of hydroxychloroquine following the COVID-19 outbreak exacerbated by reports of effectiveness in scientific journals and the media<br>• There are currently 10 licensed pharmaceutical manufacturing companies in Zambia (6 local privately owned and 4 multinational) functioning at varied capacity with supply of APIs mainly from China and India and some excipients locally. Companies in Zambia are principally involved in manufacturing, packaging and re-packaging of various dosage forms of essential medicines for the local and regional export market<br>• The pandemic has hastened discussions to improve the capacity of local pharmaceutical companies to produce medicines and commodities, and be less reliant on imports. Incentives include the government removing taxes on raw materials used in pharmaceutical manufacture of alcohol-based hand sanitizers and other related medical equipment
Zimbabwe	• Medicines are imported mainly from India (60%) and approximately 10% of essential medicines are manufactured locally<br>• The country has put in place the Medicines Logistics Information System to detect and manage shortages. Measures undertaken include redistribution of medicines and the provision of alternate medicines for first line choices. There is also the possibility of shelf-life extension of some medicines about to expire through conducting stability tests<br>• Zimbabwe currently has a shortage of medicines for NCDs even before the COVID-19 pandemic, and this will continue with most medicines procured out-of-pocket<br>• There have also been delays and rescheduling of shipments of medicines leading to shortages especially antimalaria drugs, exacerbated in the case of chloroquine and hydroxychloroquine by increased prescribing following the initial reports and hype<br>• To help address shortages, the government has announced a rebate on the import of essential ethyl alcohol, spirits, liquefiers, and hydrogen peroxide. The government has also regulated the export of medicines and PPEs via statutory instrument 90 of 2020 with currently the following pharmaceutical companies operating in Zimbabwe: Varichem, Plus 5, Central African Pharmaceutical Society, Datlabs, Pharmanova and Zim Pharmaceuticals

Table 4A – Key lessons for the future among individual African countries

Country	Key lessons and their implications
Botswana	Need for coordinated research on COVID -19 especially vaccine development
Egypt	Paying attention to 4 key pillars in the future:<br>• **Research**: Specific funding for infectious disease including prevention, early detection and treatment, encourage early publication of findings<br>• **Education**: Training programs in undergraduate and postgraduate education to improve the management of infectious and other diseases<br>• **Strategy for food and drug security.**<br>• **Good infrastructure of Information technology** to enhance communication during outbreaks especially those that require social distancing. In addition, enhance the healthcare infrastructure (staff and facilities)
Eswatini (Swaziland)	• Need to establish and strengthen local health care and emergency health systems and be less reliant on others not just to help contain the COVID-19 pandemic but help ensure routine and essential healthcare services are readily available to the public. This includes strengthening universal health coverage in accordance with SDG3 (218) as local transmission rates continue to grow<br>• Ensure multi-sectoral tackling of public health emergencies such as addressing early key issues including the availability of PPE and ventilators as well as issues surrounding the overall health of the population as well as its socioeconomic wellbeing including food as a result of such pandemics<br>• The promotion of hand hygiene and other preventive measures must be accompanied by addressing necessary structures and resources that constrain activities such activities as the scarcity of clean water<br>• Invest in local research on vaccines, therapeutics and non-medical methods of prevention as well as promote the production of pharmaceuticals, medical supplies, and equipment domestically. This will also build the economy, which will be appreciably affected by the pandemic
Ghana	• There is a need to develop national policies for disease outbreaks and responses building on national activities to tackle key issues such as antimicrobial resistance and diabetes. This can include rapid medical response centres with associated research facilities to monitor developments leading to closer integration between medical research and practice in the future<br>• Health must continue to be a government priority including training and employing health workers as well as agreed action plans and targets (including quality targets) for priority infectious and non-infectious diseases<br>• Ongoing publication of findings, alongside cross-national collaboration, to provide direction for the future
Kenya	• There is a clear need to enhance the healthcare infrastructure in Kenya including trained and adequate human resource to deal with future endemics and promote and entrench strategic interdisciplinary and multi-sectoral team work and collaboration<br>• Alongside this, a clear need for routine public health education and health promotion during pandemics to address myths and misinformation, and to promote hygiene and health seeking behaviours among the citizens<br>• A key lesson to protect against medicine and PPE shortages is to enhance local production and emphasis on appropriate and rational use<br>• Social security measures for citizens should also be put in place including affordable healthcare insurance for citizens in the low-income bracket to protect the vulnerable in the future. This is because lockdown measures may not always work in a country where the majority of citizens, especially those in informal settlements, have to work for their daily wage<br>• Need to conduct regular auditing and maintain records of movable and immovable assets in all health institutions including functional equipment and supplies

	• Need to have sound financial and food strategic reserves to sustain the country during emergencies<br>• Need for a clear tier communication and response strategy between the National and Regional governments<br>• Need to enhance and upgrade the ICT resources and services to support all essential services and functions including health and education e.g. adequate broadband and networks to support online delivery of services<br>• Need to ensure access to quality education for all to enhance literacy levels and raise the capacity for all members of the public to understand, appreciate and apply rules, guidelines and instructions in response to emergencies<br>• Need to address quality housing for all that can support activities such as home-based quarantine and/or isolation when necessary.<br>• Need to support and strengthen regional blocks with inbuilt systems and structures that ensure unified responses to emergencies with minimal border conflicts<br>• Strengthen and adequately fund the health sector and minimize reliance on donor funding and support<br>• Establish national systems for knowledge generation, synthesis, and translation to facilitate evidence-based decisions and enhance Best Practices in all key sectors and particularly the health sector
Lesotho	• There is a need to strengthen healthcare systems including all key stakeholder groups<br>• In addition, there is a need to enhance the integration of services to improve the management of infectious and non-infectious diseases especially in ambulatory care. As part of this improve the education of key healthcare workers on emerging and re- emerging infectious diseases starting in college/ university and continuing
Malawi	• There is a need to boost the resilience of the poor people in Malawi as majority of them rely on daily wages, impeding the implementation of public health measures such as lock down and distancing<br>• There is a need to improve coordination and partnerships between institutions that need each other in times of emergency to avoid overwhelming each and delaying emergency response<br>• There is also need to improve the testing capability of the local laboratories<br>• The legal framework also needs improving to minimise public health intervention challenges by the citizens as seen with legal challenges to the lockdown
Nigeria	• Urgent need to improve the healthcare system and infrastructure in the country especially the public healthcare system through improved funding from current low levels. As a result, enhance the capacity of Nigeria to respond to disease outbreaks through adequate preparedness<br>• Put social security measures into place to enhance the survival of citizens in emergency situations: food, healthcare and shelter<br>• Health education on health promoting behaviours should become routine, building on current initiatives<br>• Strengthening local production of medicines and PPEs to ensure adequate supplies during crisis and outbreaks in the future as well as improve hospitals' laboratory capacities
South Africa	• The challenge of separating COVID-19 and non-COVID patients, as well as ensuring non-COVID 19 patients do not lose out with healthcare delivery especially in other priority infectious and non-infectious diseases<br>• Similar problems arise in private facilities especially general practice<br>• Unlike other epidemics, the COVID-19 epidemic is likely to be protracted requiring a different approach. As a result, there is a need for both the public and private sectors to work together under the auspices of Universal Health Coverage (National Health Insurance)<br>• Ensure uninterrupted continuation of routine infant immunisation services and catch-up of any missed vaccinations during any pandemic to decrease

	the risk of outbreaks of dangerous vaccine-preventable diseases, which will further burden the health system and increase morbidity and mortality (219)
Sudan	• Need for preparedness against any outbreak/ epidemics/ pandemics including the provision of necessary equipment and PPE as well as a buffer stock of necessary preventive and diagnostics tools and medicines<br>• The need for a separate track for suspected patients in epidemics to reduce unintended consequences in hospitals and other healthcare centres
Uganda	• A multi-sectoral approach appeared a key success factor in reducing transmission and mortality in Uganda (Table 1) helped by an early response, open and consultative leadership and team work between the MoH and frontline healthcare professionals<br>• Lessons for the future include:<br>o Prioritise healthcare funding and functionality of the healthcare system including strengthening ambulatory care and referral systems. This includes addressing the unintended consequences including NCDs/ mental health. As part of this, seek to empower patients to improve the self-management of their disease<br>o Better manage inter-country/interstate cargo truck movement and driver testing as well as improve regional and border entry point facilities<br>o Need for more test kits and expanded testing in Uganda in the future<br>o Less reliance on donor funding for healthcare services in the future
Zambia	• Investment in developing resilient health systems including human resources especially in LMICs already burdened with high infectious diseases and NCD rates<br>• Routine instigation of infection, prevention, and control (IPC) groups including stewardship groups across healthcare sectors focussing on behavioural change (220). High rates of infection among health workers, including non-adherence to COVID-19 guidelines, endorses this need<br>• PPE for healthcare workers must be available at all times in sufficient quantity as a first step and all must participate in IPC<br>• Psychological support for healthcare workers (HCWs) in the frontline must be considered. It will be interesting to evaluate the mental health impact of COVID-19 on HCWs in the most hard-hit settings post the pandemic<br>• Countries sharing best practices, especially evidence of what works well and other adaptive strategies, that health systems can learn from each other during pandemics<br>• Address challenges with the pharmaceutical supply chain including investing in local manufacturing of medicines and other medical supplies such as PPE and diagnostic kits
Zimbabwe	• Future strategies should account for the economic situation (income, availability of basic foods, type of employment e.g. stable or unstable) and the potential impact with regards to demography, co-morbidities, access and availability of healthcare<br>• Lockdown measures should be complemented by (a) education of the general public to avoid misinformation and basic infection prevention, (b) increased preparedness among health facilities (e.g. increase the availability of PPE and equipment), training of healthcare workers in diagnosis, management and infection prevention and control; expansion of facilities to treat an increased number of cases (c) increasing capacity for testing and preparing additional laboratories to perform testing; (d) early development of strategies for resuming activities after lockdown measures have ended<br>• Improved coordination among all key stakeholder groups including scientific groups and the medical community in order that expert input is taken on board to avoid misinformation and the consequences

References

1. WHO Regional Office Africa. COVID-19 - Situation Update for the WHO African Region - 15 April 2020. External Situation Report 7. Available at URL: https://apps.who.int/iris/bitstream/handle/10665/331763/SITREP_COVID-19_WHOAFRO_20200415-eng.pdf.
2. WHO Regional Office Africa. COVID-19 - Situation Update for the WHO African Region - 5 May 2020. External Situation Report 10. Available at URL: https://reliefweb.int/sites/reliefweb.int/files/resources/SITREP_COVID-19_WHOAFRO_20200506-eng.pdf
3. Gilbert M, Pullano G, Pinotti F, Valdano E, Poletto C, Boëlle P-Y, et al. Preparedness and vulnerability of African countries against importations of COVID-19: a modelling study. Lancet. 2020;395(10227):871-7.
4. WHO Regional Office Africa. COVID-19 - Situation Update for the WHO African Region - 12 May 2020. External Situation Report 11. Available at URL: https://apps.who.int/iris/bitstream/handle/10665/332078/SITREP_COVID-19_WHOAFRO_20200513-eng.pdf.
5. WHO Regional Office Africa. COVID-19 - Situation Update for the WHO African Region - 20 May 2020. External Situation Report 12. Available at URL: https://apps.who.int/iris/bitstream/handle/10665/332150/SITREP_COVID-19_WHOAFRO_20200520-eng.pdf.
6. WHO Regional Office Africa. COVID-19 - Situation Update for the WHO African Region - 27 May 2020. External Situation Report 13. Available at URL: https://apps.who.int/iris/bitstream/handle/10665/332199/SITREP_COVID-19_WHOAFRO_20200527-eng.pdf.
7. WHO Regional Office Africa. COVID-19 - Situation Update for the WHO African Region - 1 July 2020. External Situation Report 18. Available at URL: https://apps.who.int/iris/bitstream/handle/10665/332929/SITREP_COVID-19_WHOAFRO_20200701-eng.pdf.
8. WHO. Democratic Republic of Congo. First case of COVID-19 confirmed in the Democratic Republic of the Congo. 2020. Available at URL: https://www.afro.who.int/news/first-case-covid-19-confirmed-democratic-republic-congo.
9. Mbopi-Keou F-X, Pondi J-E, Sosso MA. COVID-19 in Cameroon: a crucial equation to resolve. Lancet Infect Dis 2020. EPrint https://doi.org/10.1016/S1473-3099(20)30373-X.
10. Tih F. Cameroon confirms first coronavirus case. 2020. Available at URL: https://www.aa.com.tr/en/africa/cameroon-confirms-first-coronavirus-case/1756866.
11. WHO. Coronavirus disease 2019 (COVID-19) Situation Report – 95. 2020. Avalable at URL: https://www.who.int/docs/default-source/coronaviruse/situation-reports/20200424-sitrep-95-covid-19.pdf?sfvrsn=e8065831_4.
12. Egypt Today. Egypt records 232 new coronavirus cases, 11 deaths. 2020. Available at URL: https://www.egypttoday.com/Article/1/85044/Egypt-records-232-new-coronavirus-cases-11-deaths.
13. Hassany M, Abdel-Razek W, Asem N, AbdAllah M, Zaid H. Estimation of COVID-19 burden in Egypt. The Lancet Infectious diseases. 2020.
14. WHO. Coronavirus disease (COVID-19) Situation Report – 113. 2020. Available at URL: https://www.who.int/docs/default-source/coronaviruse/situation-reports/20200512-covid-19-sitrep-113.pdf?sfvrsn=feac3b6d_2.
15. WHO. Coronavirus disease (COVID-19) Situation Report – 120. 2020. Available at URL: https://www.who.int/docs/default-source/coronaviruse/situation-reports/20200519-covid-19-sitrep-120.pdf?sfvrsn=515cabfb_2.
16. WHO. Coronavirus disease (COVID-19) Situation Report – 128. 2020. Available at URL: https://www.who.int/docs/default-source/coronaviruse/situation-reports/20200527-covid-19-sitrep-128.pdf?sfvrsn=11720c0a_2.
17. WHO. Coronavirus disease (COVID-19) Situation Report – 163. 2020. Available at URL: https://www.who.int/docs/default-source/coronaviruse/situation-reports/20200701-covid-19-sitrep-163.pdf?sfvrsn=c202f05b_2.
18. COVID-19 Eswanti. Available at URL: http://covid19.uneswa.ac.sz/.
19. WHO Africa. COVID-19 - External Situation Report 9. 29 April 2020. Available at URL: https://apps.who.int/iris/bitstream/handle/10665/331935/SITREP_COVID-19_WHOAFRO_20200429-eng.pdf.

20. Ghana News Agency. Ghana tops Africa’s ranking for COVID-19 testing. 2020. Available at URL: https://newsghana.com.gh/ghana-tops-africas-ranking-for-covid-19-testing.
21. Reuters. Ghana turns to drones to enable faster coronavirus testing. 2020. Available at URL: https://uk.reuters.com/article/uk-health-coronavirus-ghana-drones/ghana-turns-to-drones-to-enable-faster-coronavirus-testing-idUKKBN2220HO.
22. Ghana Health Service. COVID-19. May 2020. Available at URL: https://ghanahealthservice.org/covid19/.
23. Ministry of Health Kenya. FIRST CASE OF CORONAVIRUS DISEASE CONFIRMED IN KENYA. 2020. Available at URL: https://www.health.go.ke/first-case-of-coronavirus-disease-confirmed-in-kenya/.
24. Republic of Kenya Ministry of Health. Kenya COVID-19 cases rise to 649 Nairobi, Saturday May 9, 2020. Available at URL: https://www.health.go.ke/kenya-covid-19-cases-rise-to-649-nairobi-saturday-may-9-2020/.
25. EAC. EAC COVID-19 UPDATES. 2020. Available at URL: https://www.eac.int/coronavirus.
26. SowetanLive. Malawi announces first Covid-19 cases. 2020. Available at URL: https://www.sowetanlive.co.za/news/africa/2020-04-03-malawi-announces-first-covid-19-cases/.
27. Ministry Of Health - Malawi. COVID-19 National Information Dashboard. 2020. Available at URL: http://covid19.health.gov.mw/?fbclid=IwAR3o91QivQRRhVl4Ra2dcvTU3moeeJAJFqfCtnFr3kqQP4C4BTv1-xSZweo.
28. Nigeria Centre for Disease Control. An update of COVID-19 outbreak in Nigeria. 3 May 2020. Available at URL: https://ncdc.gov.ng/diseases/sitreps/?cat=14&name=An%20update%20of%20COVID-19%20outbreak%20in%20Nigeria.
29. Medical Brief. SA’S Covid-19 situation — April 9, 2020. Available at URL: https://www.medicalbrief.co.za/archives/sas-covid-19-situation-april-9-2020/.
30. Ashly J. Is Tanzania covering up the real number of coronavirus deaths? Suspicion over official COVID-19 numbers grows as critics accuse gov't of of failing to inform public on true extent. 2020. Available at URL: https://www.aljazeera.com/news/2020/05/tanzania-covering-real-number-coronavirus-deaths-200511054304751.html.
31. Mules I. Tanzania under fire from WHO for lackluster response to COVID-19 pandemic. 2020. Available at URL: https://www.dw.com/en/tanzania-under-fire-from-who-for-lackluster-response-to-covid-19-pandemic/a-53304699.
32. Ajari EE, Kanyike AM, Ojilong D, Abdulbasit IO. COVID-19 in Uganda: Epidemiology and Response. European Journal of Medical and Educational Technologies. 2020;13(2):em2009. https://doi.org/10.30935/ejmets/8269.
33. LusakaTimes. Zambia confirms first two cases of Coronavirus. 2020. Available at URL: https://www.lusakatimes.com/2020/03/18/zambia-confirms-first-two-cases-of-coronavirus/.
34. Zambia National Public Health Institute. ZAMBIA SITUATION REPORT NO. 51. 2020. Available at URL: http://znphi.co.zm/news/wp-content/uploads/2020/05/Zambia_COVID-Situational-Report-No-51_8May2020_Final.pdf.
35. Zambia National Public Health Institute. ZAMBIA SITUATION REPORT NO. 35. 2020. Available at URL: http://znphi.co.zm/news/wp-content/uploads/2020/04/Zambia_COVID-Situational-Report-No-35_22April20_Final.pdf.
36. OCHA Zimbabwe. Situation Reports Highlights 7 May 2020. Available at URL: https://reliefweb.int/sites/reliefweb.int/files/resources/Situation%20Report%20-%20Zimbabwe%20-%206%20May%202020.pdf.
37. Kahla C. Coronavirus update: First COVID-19 case confirmed in Zimbabwe. 2020. Available at URL: https://www.thesouthafrican.com/news/zimbabwe/coronavirus-first-covid-19-case-zimbabwe-21-march/.
38. AfricaNews. Coronavirus - Zimbabwe: COVID-19 Update - 07 MAY 2020. Available at URL: https://www.africanews.com/2020/05/08/coronavirus-zimbabwe-covid-19-update-07-may-2020//
39. GardaWorld. Algeria: Government implements lockdown and curfew in Blida and Algiers March 23 /update 7. 2020. Available at URL: https://www.garda.com/crisis24/news-alerts/325896/algeria-government-implements-lockdown-and-curfew-in-blida-and-algiers-march-23-update-7.
40. Reuters. Algeria allows more businesses to-reopen amid fears of Covid-19 endangering economy. 2020. https://www.france24.com/en/20200426-algeria-coronavirus-covid-19-lockdown-restrictions.

41. GardaWorld. Algeria: Authorities ease COVID-19 restrictions June 7 /update 18. Available at URL: https://www.garda.com/crisis24/news-alerts/348746/algeria-authorities-ease-covid-19-restrictions-june-7-update-18.
42. Masisi M. Our response to Covid-19 will define our legacy. Available at URL: https://www.mmegi.bw/index.php?aid=85151&dir=2020/april/08.
43. Tau P. Covid-19: Botswana Parliament endorses 6-month state of emergency. 2020. Available at URL: https://city-press.news24.com/News/covid-19-botswana-parliament-endorses-6-month-state-of-emergency-20200409.
44. Motsamai M - Botswana Daily News. Masisi leads COVID-19 preparedness team. 2020. Available at URL: http://www.dailynews.gov.bw/news-details.php?nid=54983.
45. GARDAwORLD. Botswana: Authorities suspend commercial flights March 28 /update 1. 2020. Available at URL: https://www.garda.com/crisis24/news-alerts/327796/botswana-authorities-suspend-commercial-flights-march-28-update-1.
46. GardaWorld. Botswana: Face mask use compulsory in public May 1 /update 5. Available at URL: https://www.garda.com/crisis24/news-alerts/338786/botswana-face-mask-use-compulsory-in-public-may-1-update-5.
47. GardaWorld. Botswana: Authorities implement nationwide lockdown as of April 2 /update 3. 2020. Available at URL: https://www.garda.com/crisis24/news-alerts/328131/botswana-authorities-implement-nationwide-lockdown-as-of-april-2-update-3.
48. GardaWorld. Botswana: Nationwide lockdown measures to be lifted on May 22 /update 8. 2020. Available at URL: https://www.garda.com/crisis24/news-alerts/344411/botswana-nationwide-lockdown-measures-to-be-lifted-on-may-22-update-8.
49. Cameroon Tribune. Coronavirus Pandemic: Cameroon's Response Strategy. 2020. Available at URL: https://www.cameroon-tribune.cm/article.html/31165/fr.html/coronavirus-pandemic-cameroons-response-strategy.
50. Unah L, Mussa C. Masks, bans and questions: Inside Cameroon's COVID-19 response. 2020. Available at URL: https://www.aljazeera.com/news/2020/04/masks-bans-questions-cameroon-covid-19-response-200422134140013.html.
51. Kindzeka MK. Cameroon Eases Coronavirus Lockdown, But Neighbors Block Access. 2020. Available at URL: https://www.voanews.com/covid-19-pandemic/cameroon-eases-coronavirus-lockdown-neighbors-block-access.
52. Kindzeka MK. Cameroon Seizes Fake Coronavirus Drugs Sold by Scammers. 2020. Available at URL: https://www.voanews.com/science-health/coronavirus-outbreak/cameroon-seizes-fake-coronavirus-drugs-sold-scammers.
53. Reliefweb. Cameroon: COVID-19 Situation Report #11 (May 15 – May 28). 2020. Available at URL: https://reliefweb.int/report/cameroon/cameroon-covid-19-situation-report-11-may-15-may-28.
54. GardaWorld. Congo: Authorities implement quarantine measures March 3. 2020. Available at URL: https://www.garda.com/crisis24/news-alerts/319746/congo-authorities-implement-quarantine-measures-march-3.
55. Tasamba J. DR Congo extends state of emergency amid COVID-19 - MPs need to put aside ethnic and tribal differences in face of coronavirus, speaker of Lower House of Parliament. 2020. Available at URL: https://www.aa.com.tr/en/africa/dr-congo-extends-state-of-emergency-amid-covid-19/1816681.
56. WHO Africa. How Ebola is helping the Democratic Republic of the Congo prepare for coronavirus disease. Available at URL: https://www.afro.who.int/photo-story/how-ebola-helping-democratic-republic-congo-prepare-coronavirus-disease.
57. GardaWorld. Congo: Government extends COVID-19 lockdown measures until May 15 /update 4. 2020. Available at URL: https://www.garda.com/crisis24/news-alerts/338286/congo-government-extends-covid-19-lockdown-measures-until-may-15-update-4.
58. GardaWorld. Congo: Authorities extend nationwide curfew orders until June 20 /update 6. 2020. Available at URL: https://www.garda.com/crisis24/news-alerts/348541/congo-authorities-extend-nationwide-curfew-orders-until-june-20-update-6.
59. UNICEF. DEMOCRATIC REPUBLIC OF CONGO: COVID-19 Situation Report – #05. 2020. Available at URL: https://reliefweb.int/sites/reliefweb.int/files/resources/DRC%20-%20COVID19%20-%20Weekly%20SitRep%20%235%20EN.pdf.
60. Sahmoud T. Estimation of COVID-19 burden in Egypt. The Lancet Infectious diseases. 2020.
61. Egypt Independent. Egypt’s Ministry of Health converts 30 hospitals to quarantine hospitals. 2020. Available at URL: https://egyptindependent.com/egypts-ministry-of-health-converts-30-hospitals-to-quarantine-hospitals/.

62. GardaWorld. Egypt: Government to implement nighttime curfew from March 25 /update 11. 2020. Available at URL: https://www.garda.com/crisis24/news-alerts/325991/egypt-government-to-implement-nighttime-curfew-from-march-25-update-11.
63. Middle East Eye. 'Staying home is for the rich': Social distancing is a luxury Egypt's poor can't afford - A lack of social security and overcrowding means obeying the Covid-19 curfew is impossible for millions of Egyptians. 2020. Available at URL: https://www.middleeasteye.net/news/coronavirus-egypt-social-distancing-poor-cannot-afford.
64. Negida A. Estimation of COVID-19 burden in Egypt. The Lancet Infectious diseases. 2020.
65. Egypt Today. Egyptian Armed Forces continue handing out face masks to citizens to fight coronavirus. 2020. Available at URL: https://www.egypttoday.com/Article/1/85157/Egyptian-Armed-Forces-continue-handing-out-face-masks-to-citizens.
66. Egypt Independent. Video: Egyptian Armed Forces lead fight against coronavirus. 2020. Available at URL: https://egyptindependent.com/video-egyptian-armed-forces-lead-fight-against-coronavirus/.
67. El Tawil. Sisi visits field quarantine hospital established by Egyptian Armed Force. 2020. Available at URL: https://www.egypttoday.com/Article/1/89027/Sisi-visits-field-quarantine-hospital-established-by-Egyptian-Armed-Forces.
68. Ahramonline. Egypt's Sisi tours Armed Forces medical isolation facilities. 27 June 2020. Available at URL: http://english.ahram.org.eg/NewsContent/1/64/373091/Egypt/Politics-/UPDATED-Egypts-Sisi-tours-Armed-Forces-medical-iso.aspx.
69. Egypt State Information Service. Sudan thanks Egypt for medical aid to curb COVID-19. 5 May 2020. Available at URL: https://www.sis.gov.eg/Story/145975/Sudan-thanks-Egypt-for-medical-aid-to-curb-COVID-19?lang=en-us.
70. Egypt Today. Egypt sends medical aid to South Sudan to combat COVID-19. 19 May 2020. Available at URL: https://www.egypttoday.com/Article/1/87883/Egypt-sends-medical-aid-to-South-Sudan-to-combat-COVID.
71. XINHUANET. Egypt sends medical aid to DC Congo, Zambia to fight COVID-19. 2 June 2020. Available at URL: http://www.xinhuanet.com/english/2020-06/02/c_139106090.htm.
72. Ministry of Health Eswatini, WHO, UNICEF. National Contingency for Novel Coronavirus (COVID-19) . Available at URL: https://www.unicef.org/eswatini/media/771/file/National-novel-coronavirus-preparedness-and-response-plan-2020.pdf.
73. GardaWorld. Eswatini: Partial lockdown comes into effect March 27 /update 1. 2020. Available at URL: https://www.garda.com/crisis24/news-alerts/326956/eswatini-partial-lockdown-comes-into-effect-march-27-update-1.
74. Eswatini Government Gazette Extraordinary March 27 2020. No 53. Available at URL: http://www.gov.sz/images/CORONA/The-Coronavirus-COVID-19-Regulations-2020.pdf.
75. ESWATINI GOVERNMENT GAZETTE EXTRAORDINARY. THE CORONAVIRUS (COVID-19) REGULATIONS, 2020. 27 March 2020. Available at URL: http://www.gov.sz/images/CORONA/The-Coronavirus-COVID-19-Regulations-2020.pdf.
76. GardaWorld. Eswatini: Authorities reinstate COVID-19 lockdown measures from April 24 /update 4. 2020. Available at URL: https://www.garda.com/crisis24/news-alerts/335771/eswatini-authorities-reinstate-covid-19-lockdown-measures-from-april-24-update-4.
77. GOVERNMENT OF THE KINGDOM OF ESWATINI. PRIME MINISTER’S STATEMENT - PARTIAL LOCKDOWN STRENGTHENED MEASURES. 2020. Available at URL: http://www.gov.sz/images/CORONA/PM-statement-22-April-2020.pdf.
78. Swaziland News. Chinese Embassy: Eswatini to benefit from billionaire’s Coronavirus equipment donation. 2020. Available at URL: http://www.swazilandnews.co.za/fundza.php?nguyiphi=261.
79. EU in Swaziland. 23 April 2020. Available at URL: https://twitter.com/EUinSwaziland.
80. Everington K. Taiwan donates 20,000 units of medical supplies to Eswatini to fight coronavirus. Available at URL: https://www.taiwannews.com.tw/en/news/3907591.
81. AfricaNews. Coronavirus – Eswatini: COVID-19 update – 22 April 2020. Available at URL: https://www.africanews.com/2020/04/22/coronavirus-eswatini-covid-19-update-22-april-2020//.
82. AfricaNews. Coronavirus – Eswatini: The Ministry of Health emphasizes that the definition of a suspect case has changed. 2020. Available at URL: https://www.africanews.it/english/coronavirus-eswatini-the-ministry-of-health-emphasizes-that-the-definition-of-a-suspect-case-has-changed/.
83. Aljazeera News Ethiopia. Ethiopia declares state of emergency to fight coronavirus - Prime Minister Abiy Ahmed warns of 'grave legal measures' against anyone who undermines the fight against the pandemic. 2020. Available at URL: https://www.aljazeera.com/news/2020/04/ethiopia-declares-state-emergency-fight-covid-19-200408142519485.html.

84. Tekle T-A. Ethiopia Bans Public Events, Closes Schools As It Confirms Fifth COVID-19 Case. 2020. Available at URL: https://allafrica.com/stories/202003170202.html.
85. Embassy of the Federal Democratic Republic of Ethiopia, London, UK. Ethiopia declares State of Emergency to curb transmission of Coronavirus. 2020. Available at URL: https://www.ethioembassy.org.uk/ethiopia-declares-state-of-emergency-to-curb-transmission-of-coronavirus/.
86. Shaban ARA. Ethiopia coronavirus: Key updates between March 16 - May 16. 2020. Available at URL: https://www.africanews.com/2020/05/19/ethiopia-s-coronavirus-rules-crowd-ban-free-transport-regulate-essentials-etc/.
87. ReliefWeb. Ethiopia Taking Measures to Prevent Ebola Virus. 2018. Available at URL: https://reliefweb.int/report/democratic-republic-congo/ethiopia-taking-measures-prevent-ebola-virus.
88. KPMG. Ghana-COVID-19-Related Travel Restrictions and Safety Directives. 2020. Available at URL: https://home.kpmg/gh/en/home/insights/2020/04/gh-covid-19-related-travel-restrictions-and-safety-.html.
89. Ghana News Agency. Ghana closes borders Sunday to curb coronavirus spread. 2020. Available at URL: https://newsghana.com.gh/ghana-closes-borders-sunday-to-curb-coronavirus-spread/
90. Ngnenbe T. Govt selects five companies to produce 3.6 million nose masks. 2020. Available at URL: https://www.graphic.com.gh/news/general-news/govt-selects-five-companies-to-produce-3-6-million-nose-masks.html.
91. Arhinful E. COVID-19 Local production of PPE begins in Ghana. 2020. Available at URL: https://citinewsroom.com/2020/04/covid-19-local-production-of-ppe-begins-in-ghana-photos/.
92. Citi Newsroom. FDA recommendations for homemade face masks. 2020. Available at URL: https://citinewsroom.com/2020/04/fda-recommendations-for-homemade-face-masks/
93. Mbewa DO. Ghana makes wearing of face masks in public mandatory. 15 June 2020. https://africa.cgtn.com/2020/06/15/ghana-makes-wearing-of-face-masks-in-public-mandatory/.
94. GhanaWeb. Zipline Ghana launches emergency delivery of COVID-19 test samples. 2020. Available at URL: https://www.ghanaweb.com/GhanaHomePage/NewsArchive/Zipline-Ghana-launches-emergency-delivery-of-COVID-19-test-samples-929806
95. Dogbey SA. Jack Ma’s donation of PPEs, test kits lands in Ghana. 2020. Available at URL: https://www.myjoyonline.com/news/national/jack-mas-donation-of-ppes-test-kits-lands-in-ghana/.
96. Graphic Online. Coronavirus: Ghana has over 400 ventilators. 2020. Available at URL: https://www.graphic.com.gh/news/general-news/coronavirus-ghana-has-over-400-ventilators.html. .
97. Republic of Kenya Ministry of Health. UPDATE ON COVID-19 AS AT 25th FEBRUARY 2020. Available at URL: https://www.health.go.ke/wp-content/uploads/2020/02/COV-2019-Press-Release-_25thFeb-2020-converted.pdf.
98. Smith G. Stamping out misinformation in Kenya's COVID-19 fight - Volunteers and activists work to diffuse spread of fake information, false 'remedies' as Kenya steps up pandemic battle. 2020. Available at URL: https://www.aljazeera.com/news/2020/04/stamping-misinformation-kenya-covid-19-fight-200424195805081.html.
99. Moore D. Fury in Kenya over police brutality amid coronavirus curfew - Growing calls for police to be held accountable for excessive violence used to enforce COVID-19-related restrictions. 2020. Available at URL: https://www.aljazeera.com/news/2020/04/fury-kenya-police-brutality-coronavirus-curfew-200402125719150.html.
100. AA News. Kenya confirms first coronavirus case - Patient is Kenyan national who recently traveled to US, says health official. 13 March 2020. https://www.aa.com.tr/en/africa/kenya-confirms-first-coronavirus-case/1764717.
101. Ali S. Kenyans Under Brute Attack Amidst COVID-19 Curfew. 6 April 2020. Available at URL: https://www.indepthnews.net/index.php/the-world/africa/3439-kenyans-under-brute-attack-amidst-covid-19-curfew.
102. The Citizen. Kenya imposes lockdown in Covid-19 hotspots as cases shoot to 582. 2020. Available at URL: https://www.msn.com/en-xl/news/other/kenya-imposes-lockdown-in-covid-19-hotspots-as-cases-shoot-to-582/ar-BB13HdO1.
103. Daily Nation. Covid-19: Mandera officially on lockdown after cases increase. 2020. Available at URL: https://www.nation.co.ke/counties/mandera/Covid-19-Mandera-officially-on-lockdown/1183298-5531714-grhcp8/index.html.
104. Aljazeera News. COVID-19: Kenya bans travel in and out of Nairobi, other areas. 6 April 2020. Available at URL: https://www.aljazeera.com/news/2020/04/covid-19-kenya-bans-travel-nairobi-areas-200406163601579.html.

105. World Aware. COVID-19 Alert: Kenya Extends International Flight Ban as of April 6-May 6. 2020. Available at URL: https://www.worldaware.com/covid-19-alert-kenya-extends-international-flight-ban-april-6-may-6.
106. Mutahi B. Coronavirus: The fear of being sentenced to a Kenyan quarantine centre. 2020. Available at URL: https://www.bbc.co.uk/news/world-africa-52326316.
107. Roberto M. Coronavirus: Kenyan company begins mass production of face masks to counter spread of COVID-19. Available at URL: https://www.tuko.co.ke/348695-coronavirus-kenyan-company-begins-mass-production-face-masks-counter-spread-covid-19.html.
108. MUASYA P. Two firms begin mass face masks production. 2020. Available at URL: https://www.standardmedia.co.ke/article/2001367532/two-firms-begin-mass-face-masks-production.
109. MUENI J. COVID-19: Health Officials Visit PPE Assembling Firm To Assess Quality Assurance. 2020. Available at URL: https://www.capitalfm.co.ke/news/2020/04/covid-19-health-officials-visit-ppe-assembling-firm-to-assess-quality-assurance/.
110. Kajilwa G. Kenya receives test kits donation from Jack Ma. 2020. Available at URL: https://www.standardmedia.co.ke/article/2001365546/kenya-receives-test-kits-donation-from-jack-ma.
111. The World Bank. Kenya Receives $50 Million World Bank Group Support to Address COVID-19 Pandemic. 2020. Available at URL: https://www.worldbank.org/en/news/press-release/2020/04/02/kenya-receives-50-million-world-bank-group-support-to-address-covid-19-pandemic.
112. GATHURA G. 400 Kenya health workers wanted for Covid-19 vaccine trials. 2020. Available at URL: https://www.standardmedia.co.ke/health/article/2001372627/vaccine-test-targets-400-kenyan-health-workers.
113. JKUAT. JKUAT Trio Develop Apps to Trace, Triage and Manage COVID-19. 2020. Available at URL: http://www.jkuat.ac.ke/jkuat-trio-develop-contact-tracing-and-case-management-apps/.
114. JKAUT. JKUAT's Team of Engineers Invent Ventilators to Fill Shortage. 2020. Available at URL: http://www.jkuat.ac.ke/jkuats-team-of-engineers-invent-ventilators-to-fill-shortage/.
115. University of Nairobi. UoN Ventilators, ready for production model. 2020. Available at URL: https://www.uonbi.ac.ke/news/uon-ventilators-ready-production-model.
116. Kenyatta University. K.U Makes Ventilators. 2020. Available at URL: http://www.ku.ac.ke/component/k2/item/1655-k-u-makes-ventilators.
117. Wanzala J. Kenyans Are Innovative in Finding Ways to Fight COVID-19. 2020. Available at URL: https://www.indepthnews.net/index.php/the-world/africa/3496-kenyans-are-innovative-in-finding-ways-to-fight-covid-19.
118. Ministry of Health Kenya. Practical Guide for Continuity of Reproductive, Maternal, Newborn and Family Planning Care and Services in the Background of COVID19 Pandemic in Kenya. Available at URL: https://depts.washington.edu/kenyares/wp-content/uploads/2020/04/COVID19-RMNH-GUIDELINES-CIRCULATE.pdf.pdf.pdf.
119. GardaWorld. Lesotho: Authorities announce lockdown and entry restrictions due to COVID-19 effective March 30. 2020. Available at URL: https://www.garda.com/crisis24/news-alerts/327786/lesotho-authorities-announce-lockdown-and-entry-restrictions-due-to-covid-19-effective-march-30.
120. The Citizen. Lesotho to lift lockdown as virus remains undetected. 2020. Available at URL: https://citizen.co.za/news/covid-19/2279373/lesotho-to-lift-lockdown-as-virus-remains-undetected/.
121. GardaWorld. Lesotho: COVID-19 lockdown measures eased May 5 /update 2. 2020. Available at URL: https://www.garda.com/crisis24/news-alerts/339401/lesotho-covid-19-lockdown-measures-eased-may-5-update-2.
122. Munthali GN, Xuelian W, Covid-19 Outbreak on Malawi Perspective. Electronic Journal of General Medicine. 2020;17(4):em210.
123. Chilungia Z. Mutharika shakes up Malawi Covid-19 committee Oppositio leade lowe drafted in. 2020. Available at URL: https://www.nyasatimes.com/mutharika-shakes-up-malawi-covid-19-committee-opposition-leader-lowe-drafted-in.
124. GaraWold. Malawi: Authorities suspend international flights as of April 1 /update 1. 2020. Available at URL: https://www.garda.com/crisis24/news-alerts/328586/malawi-authorities-suspend-international-flights-as-of-april-1-update-1.
125. United Nations Malawi - COVID-19 Update. 2020. Available at URL: https://reliefweb.int/sites/reliefweb.int/files/resources/Malawi-COVID-19-Situation-Update-03.04.20.pdf.
126. GardaWorld. Malawi: Nationwide lockdown imposed due to COVID-19 April 14 /update 4. 2020. Available at URL: https://www.garda.com/crisis24/news-alerts/332471/malawi-nationwide-lockdown-imposed-due-to-covid-19-april-14-update-4.

127. Mweninguwe R. Malawi judge blocks lockdown. Available at URL: https://www.dandc.eu/en/article/malawis-covid-19-lockdown-has-been-suspended-legal-action.
128. GardaWorld. Malawi: Nationwide lockdown measures come into effect from April 18 /update 5. 2020. Available at URL: https://www.garda.com/crisis24/news-alerts/333176/malawi-nationwide-lockdown-measures-come-into-effect-from-april-18-update-5.
129. United Nations Malawi. Malawi Govt launches new COVID-19 workplace guidelines. 2020. Available at URL: https://mw.one.un.org/malawi-govt-launches-new-covid-19-workplace-guidelines/.
130. GardaWorld. Namibia: Government closes schools and suspends flights between Qatar, Ethiopia, and Germany March 15. 2020. Available at URL: https://www.garda.com/crisis24/news-alerts/323396/namibia-government-closes-schools-and-suspends-flights-between-qatar-ethiopia-and-germany-march-15.
131. GardaWorld. Namibia: Authorities ban entry of all foreign nationals due to COVID-19 March 24 /update 1. Available at URL: https://www.garda.com/crisis24/news-alerts/326116/namibia-authorities-ban-entry-of-all-foreign-nationals-due-to-covid-19-march-24-update-1.
132. Shikongo A. Namibia: Partial Lockdown in Effect from Friday. 2020. Available at URL: https://allafrica.com/stories/202003241023.html.
133. GardaWorld. Namibia: Government extends partial lockdown until May 4 due to COVID-19 /update 2. 2020. Available at URL: https://www.garda.com/crisis24/news-alerts/332441/namibia-government-extends-partial-lockdown-until-may-4-due-to-covid-19-update-2.
134. GARDAWORLD. Namibia: Government to ease COVID-19 restrictions from May 5 /update 3. 2020. Available at URL: https://www.garda.com/crisis24/news-alerts/337911/namibia-government-to-ease-covid-19-restrictions-from-may-5-update-3.
135. Nigeria Centre for Disease Control. Guidelines and Protocols. 2020. Available at URL: https://ncdc.gov.ng/diseases/guidelines.
136. Di Caro B, Beech B. COVID-19 in Africa: insights from our 23 April WHO media briefing. 2020. Available at URL: https://www.weforum.org/agenda/2020/04/covid19-in-africa-our-media-briefing-with-who
137. Ogoina D. COVID-19: The Need for Rational Use of Face Masks in Nigeria. Am J Trop Med Hyg. 2020.
138. President Federal Republic of Nigeria. The Quarantine Act. 30 March 2020. Available at URL: https://covid19.ncdc.gov.ng/media/files/COVID-19_REGULATIONS_2020_20200330214102.pdf.
139. The Straits Times. Coronavirus: Lagos says masks compulsory to stop virus. 26 April 2020. Available at URL: https://www.straitstimes.com/world/africa/coronavirus-lagos-says-masks-compulsory-to-stop-virus.
140. Oyeyemi T. REMARKS BY THE SGF/CHAIRMAN OF THE PTF COVID-19 AT THE NATIONAL BRIEFING OF WEDNESDAY, 6TH MAY, 2020. Available at URL: https://fmic.gov.ng/remarks-by-the-sgf-chairman-of-the-ptfcovid19-at-the-national-briefing-of-wednesday-6th-may-2020/.
141. Adeneyo A. EU donates €50 million to assist Nigeria fight against COVID-19. 2020. Available at URL: https://www.kemifilani.ng/2020/04/eu-donates-e50-million-to-assist-nigeria-fight-against-covid-19.html.
142. Onyedinefu G. Coronavirus: NAFDAC alerts Nigerians to circulation of fake Chloroquine. 2020. Available at URL: https://businessday.ng/coronavirus/article/coronavirus-nafdac-alerts-nigerians-to-circulation-of-fake-chloroquine/.
143. AfricaNews. Coronavirus - Rwanda: Update on COVID-19 Coronavirus - 18 April 2020. Available at URL: https://www.africanews.com/2020/04/19/coronavirus-rwanda-update-on-covid-19-coronavirus-18-april-2020//.
144. Tasamba J. COVID-19: Rwanda, DR Congo make mask wearing mandatory. Available at URL: https://www.aa.com.tr/en/africa/covid-19-rwanda-dr-congo-make-mask-wearing-mandatory/1810165.
145. Condo J, Uwizihiwe JP, Nsanzimana S. Rwanda’s success in tackling COVID-19. Nature 2020; 581: 384
146. AA Agency. Rwanda uses drones to bring cancer drugs to patients - Rwandan NGO teams up with US logistics firm to deliver cancer medicines to where patients live amid lockdown. 2020. Available at URL: https://www.aa.com.tr/en/africa/rwanda-uses-drones-to-bring-cancer-drugs-to-patients/1820739.
147. Balasingam M. Drones in medicine-The rise of the machines. International journal of clinical practice. 2017;71(9).

148. Toor A. This startup is using drones to deliver medicine in Rwanda - Zipline will begin delivering blood and drugs across the country in July. 2016. Available at URL: https://www.theverge.com/2016/4/5/11367274/zipline-drone-delivery-rwanda-medicine-blood.
149. Tasamba J. Rwanda takes steps to ease COVID-19 lockdown. 1 May 2020. Available at URL: https://www.aa.com.tr/en/africa/rwanda-takes-steps-to-ease-covid-19-lockdown/1825174.
150. Anadolu Agency. Rwanda reinstates COVID-19 lockdown in parts of capital. 26 June 2020. Available at URL: https://www.aa.com.tr/en/africa/rwanda-reinstates-covid-19-lockdown-in-parts-of-capital/1891116.
151. WHO. COVID-19 Strategic Preparedness and Response Plan - OPERATIONAL PLANNING GUIDELINES TO SUPPORT COUNTRY PREPAREDNESS AND RESPONSE. 2020. Available at URL: https://www.who.int/docs/default-source/coronaviruse/covid-19-sprp-unct-guidelines.pdf.
152. South African Government. President Cyril Ramaphosa: Measures to combat Coronavirus COVID-19 epidemic. 2020. Avalable at URL: https://www.gov.za/speeches/statement-president-cyril-ramaphosa-measures-combat-covid-19-epidemic-15-mar-2020-0000.
153. Republic of South Africa. Government Notices 26 March 2020. Available at URL: https://www.gov.za/sites/default/files/gcis_document/202003/43168reg11067gon419.pdf.
154. Republic of South Africa. Government Notices 29 April 2020. Available at URL: https://www.gov.za/sites/default/files/gcis_document/202004/43258rg11098gon480.pdf.
155. Msomi N, Pilane P. #LockdownSA: Ramaphosa announces SA lockdown to curb Covid-19 spread. 2020. Available at URL: https://health-e.org.za/2020/03/23/lockdownsa-ramaphosa-announces-sa-lockdown-to-curb-covid-19-spread/.
156. South African Government. President Cyril Ramaphosa: Update on Coronavirus COVID-19 lockdown. 2020. Available at URL: https://www.gov.za/speeches/president-cyril-ramaphosa-update-coronavirus-covid-19-lockdown-30-mar-2020-0000.
157. Republic of South Africa - South African Government Agency News. SA to move from level 5 lockdown to level 4. 23 April 2020. Available at URL: https://www.sanews.gov.za/south-africa/sa-move-level-5-lockdown-level-4.
158. Republic of South Africa. Summary of alert levels. 2020. Available at URL: https://www.stateofthenation.gov.za/.
159. Schroder M, Bossert A, Kersting M, Aeffner S, Coetzee J, Timme M et al. COVID-19 in Africa: outbreak despite interventions? 2020. MedRxiv preprint doi: https://doi.org/10.1101/2020.04.24.20077891.t. Available at URL: https://www.medrxiv.org/content/10.1101/2020.04.24.20077891v1.full.pdf.
160. Department of Health Republic of South Africa. USE OF CLOTH FACE-MASKS BY MEMBERS OF THE GENERAL PUBLIC IN SOUTH AFRICA DURING THE COVID-19 PANDEMIC. 2020. Available at URL: https://sacoronavirus.co.za/2020/04/21/use-of-cloth-face-masks-by-members-of-the-general-public-in-south-africa-during-the-covid-19-pandemic/
161. Maringira G. Covid-19: Social Distancing and Lockdown in Black Townships in South Africa. 2020. Available at URL: https://kujenga-amani.ssrc.org/2020/05/07/covid-19-social-distancing-and-lockdown-in-black-townships-in-south-africa/.
162. Depart of Health Republic of South Africa. COVID-19 Disease: Infection Prevention and Control Guidelines. April 2020. Available at URL: https://www.idealhealthfacility.org.za/docs/Manuals-and-Handbooks/COVID%2019%20Disease%20Infection%20Prevention%20and%20Control%20Guidelines%20Version%201%20-%20%20April%202020.pdf.
163. Githahu M. Covid-19: App launched to track symptoms, alleviating pressure on health-care systems. 2020. Available at URL: https://www.iol.co.za/capeargus/news/covid-19-app-launched-to-track-symptoms-alleviating-pressure-on-health-care-systems-46412366.
164. Wits University. Wits engineers make face shields to protect healthcare workers. 2020. Available at URL: https://www.wits.ac.za/covid19/covid19-news/latest/wits-engineers-make-face-shields-to-protect-healthcare-workers.html.
165. Reuters. South Africa's arms firm Denel to produce ventilators in coronavirus fight. 2020. Available at URL: https://www.dailymaverick.co.za/article/2020-04-06-south-africas-arms-firm-denel-to-produce-ventilators-in-coronavirus-fight/.
166. Wild S. How HIV expertise is helping South Africa tackle coronavirus. 2020. Available at URL: https://www.weforum.org/agenda/2020/04/south-africa-hiv-coronavirus-covid19-pandemic-health-virus.
167. van den Heever A. Projections on SA health system and whether there are enough hospital beds to cope. 2020. Available at URL: https://www.dailymaverick.co.za/article/2020-03-16-projections-on-sa-health-system-and-whether-there-enough-hospital-beds-to-cope/.

168. Joynt GM, Gopalan DP, Argent AA, Chetty S, Wise R, Lai VKW, et al. The Critical Care Society of Southern Africa Consensus Statement on ICU Triage and Rationing (ConICTri). South African medical journal. 2019;109(8b):613-29.
169. Motse O. Coronavirus: Bloemfontein hospital ups ICU capacity by 70 beds. 2020. Available at URL: https://www.ofm.co.za/article/centralsa/286902/-coronavirus-bloemfontein-hospital-ups-icu-capacity-by-70-beds.
170. Department of Health Republic of South Africa. SA's COVID-19 epidemic: Trends & Next steps. 2020. Available at URL: https://sacoronavirus.co.za/2020/04/13/sas-covid-19-epidemic-trends-next-steps/.
171. Medical Brief. SA's COVID-19 roundup – 23 April 2020: 45,000 deaths expected over next 2-3 years. April 22, 2020. Available from URL: https://www.medicalbrief.co.za/archives/sas-covid-19-roundup-23-april-2020-45000-deaths-expected-over-next-2-3-years/. .
172. Davis R. Viral outbreak: Fake news spreads in SA in tandem with Covid-19. 2020. Available at URL: https://www.dailymaverick.co.za/article/2020-03-31-viral-outbreak-fake-news-spreads-in-sa-in-tandem-with-covid-19/.
173. SAHPRA. SAHPRA cautions against medicine stockpiling including Chloroquine containing products. 2020. Available at URL: http://www.sahpra.org.za/wp-content/uploads/2020/03/SAHPRA-communique_Chloroquine-Stockpiling_23032020.pdf.
174. Masango TA. Misuse of chloroquine, azithromycin and lopinavir-ritonavir for the prevention and/or potential treatment of Covid-19. 2020. Available atURL: https://www.biznews.com/briefs/2020/03/24/pharmacy-body-anti-malaria-drugs-covid-19.
175. Lakay B. Your 6-month prescription expiring? Your pharmacist may extend it, new regulations state. 2020. Available at URL: https://www.health24.com/Medical/Infectious-diseases/Coronavirus/your-6-month-prescription-expiring-your-pharmacist-may-extend-it-new-regulations-state-20200430-2.
176. Eltahir N, Abdelaziz K, Siddig E. Few ventilators, little cash: Sudan braces for coronavirus test. 22 April 2020. Available at URL: https://uk.reuters.com/article/us-health-coronavirus-sudan/few-ventilators-little-cash-sudan-braces-for-coronavirus-test-idUKKCN22426N

177. Weston M. Coronavirus reaches Sudan, one of the countries least equipped to cope with it. 24 March 2020. Available at URL: https://mg.co.za/africa/2020-03-24-coronavirus-reaches-sudan-one-of-the-countries-least-equipped-to-cope-with-it/.
178. HCT/UNCT. Sudan: COVID-19 Country Preparedness and Response Plan CPRP, 2020. Available for URL: https://reliefweb.int/sites/reliefweb.int/files/resources/200504_Sudan%20HCT-UNCT%20Covid-19%20Plan.pdf.
179. XinhuaNet. Tanzanian president rules out lockdown amid COVID-19 pandemic. 2020. Available at URL: http://www.xinhuanet.com/english/2020-04/23/c_138999856.htm.
180. EAC Secretariat. East African Community COVID-19 Response Plan. 2020. Available at URL: https://www.eac.int/coronavirus.
181. Aceng JR. Update on Uganda's preparedness situation following the 2019 Novel Coronavirus outbreak in China and other countries around the World. 2020. Available at URL: https://www.afro.who.int/news/update-ugandas-preparedness-situation-following-2019-novel-coronavirus-outbreak-china-and.
182. Akello G, Hopwood J. How do we measure the effectiveness of lockdown in Uganda against COVID-19? 2020. Available at URL: https://blogs.lse.ac.uk/africaatlse/2020/04/27/how-to-measure-effective-of-lockdown-in-uganda-against-covid-19/.
183. Calvin-Smith G. Covid-19: Uganda extends lockdown for two weeks but slowly eases measures. 2020. Available at URL: https://www.france24.com/en/africa/20200505-uganda-extends-lockdown-for-two-weeks-but-slowly-eases-measures.
184. GardaWorld. Uganda: Authorities ease certain restrictions and extend lockdown by 14 days May 4 /update 6. Available at URL: https://www.garda.com/crisis24/news-alerts/339056/uganda-authorities-ease-certain-restrictions-and-extend-lockdown-by-14-days-may-4-update-6.
185. GardaWorld. Uganda: Authorities announce 14-day nationwide lockdown April 1 /update 3. 2020. Available at URL: https://www.garda.com/crisis24/news-alerts/328101/uganda-authorities-announce-14-day-nationwide-lockdown-april-1-update-3.
186. Mariam A. Uganda: 'Treatment strategy helps recovery of COVID-19 patients' - Attending to other ailments of COVID-19 patients has helped in their fast recovery, say Ugandan doctors. 2020. Available at URL: https://www.aa.com.tr/en/africa/uganda-treatment-strategy-helps-recovery-of-covid-19-patients-/1832024.

187. The Independent. EAC to embark on joint tests for COVID-19. 2020. Available at URL: https://www.independent.co.ug/eac-to-embark-on-joint-tests-for-covid-19/.
188. The East African. Covid-19: Kenya truck drivers protest Uganda's border rules. 2020. Available at URL: https://www.theeastafrican.co.ke/news/ea/Kenya-truck-drivers-protest-Uganda-border-rules/4552908-5536468-jdjvikz/index.html.
189. Ministry of Health Uganda. UPDATE ON COVID-19 RESPONSE IN UGANDA: TRUCK DRIVERS AND GUIDELINES ON USE OF MASKS. 2020. Available at URL: https://www.health.go.ug/covid/document/update-on-covid-19-response-in-uganda-truck-drivers-and-guidelines-on-use-of-masks/.
190. Xinhua. Uganda starts relief food distribution for vulnerable people amid COVID-19 lockdown. 2020. Available at URL: http://www.xinhuanet.com/english/2020-04/04/c_138947580.htm.
191. Zambia National Public Health Institute. PRESS STATEMENT ON COVID-19 AND ADDITIONAL PREVENTIVE AND CONTROL MEASURES INTRODUCED BY THE GOVERNMENT OF THE REPUBLIC OF ZAMBIA. 2020. Available at URL: https://znphi.co.zm/news/press-statement-on-covid-19-and-additional-preventive-and-control-measures-introduced-by-the-government-of-the-republic-of-zambia/.
192. Government of Zambia. Statutory Instrument No. 22 of 2020. 2020. Available at URL: https://zambialii.org/zm/blog/Statutory%20Instrument%20No.22%20of%202020%20%282%29.pdf
193. Anadolu Agency. Zambia shuts border with Tanzania to curb COVID-19 - Development comes after African country witnessed highest spike in infections last week. 2020. Available at URL: https://www.aa.com.tr/en/africa/zambia-shuts-border-with-tanzania-to-curb-covid-19/1836623.
194. Charity Today. New funding to adapt Zambia’s Malaria network during COVID-19. 2020. Available at URL: https://www.charitytoday.co.uk/new-funding-to-adapt-zambias-malaria-network-during-covid-19/.
195. Xinhua. China's Jack Ma gives Zambia COVID-19 medical supplies. 2020. Available at URL: https://www.focac.org/eng/zfgx_4/jmhz/t1760745.htm.
196. Editor Food Business Africa. Trade Kings Foundation launches hygiene drive against Covid-19 in Lusaka. 2020. Available at URL: https://www.foodbusinessafrica.com/2020/04/22/trade-kings-foundation-launches-hygiene-drive-against-covid-19-in-lusaka/.
197. Dzobo M, Chitungo I, Dzinamarira T. COVID-19: a perspective for lifting lockdown in Zimbabwe. The Pan African Medical Journal. 2020;35 (Supp 2):13.
198. The Herald. Government declares Coronavirus a national disaster . . . Independence celebrations cancelled. 2020. Available at URL: https://www.herald.co.zw/government-declares-coronavirus-a-national-disaster-independence-celebrations-cancelled/.
199. Marawanyika G. Zimbabwe Extends Covid-19 National Lockdown by 14 Days. 2020. Availanble at URL: https://www.bloomberg.com/news/articles/2020-05-01/zimbabwe-extends-covid-19-national-lockdown-by-14-days.
200. ReliefWeb. COVID-19 FACT SHEET Zimbabwe. 2020. Available at URL: https://reliefweb.int/sites/reliefweb.int/files/resources/WVZ%20COVID-19%20Response%20Fact%20Sheet_22%20April%202020.pdf.
201. Xinhua. Zimbabwe receives donation from China's Jack Ma to fight COVID-19. 2020. Available at URL: http://en.people.cn/n3/2020/0325/c90000-9672146.html.
202. Dzirutwe M. Zimbabwe president threatens 20 years jail over fake Covid-19 statement. 2020. Available at URL: https://www.sowetanlive.co.za/news/africa/2020-04-14-zimbabwe-president-threatens-20-years-jail-over-fake-covid-19-statement/.
203. GhanaWeb. Pharmaceutical companies to produce 70% of country’s drug needs. 2020. Available at URL: https://www.ghanaweb.com/GhanaHomePage/NewsArchive/Pharmaceutical-companies-to-produce-70-of-country-s-drug-needs-936016.
204. Bokpe S. Watch out for counterfeit chloroquine on the market-FDA warns. 2020. Available at URL: https://www.theghanareport.com/fda/.
205. N Gage Consuting. Egypt's Pharmaceutical Sector Following Bold Economic Reforms: CHALLENGES AND OPPORTUNITIES. 2017. Available at URL: https://www.ngage-consulting.com/downloads/Pharmaceutical_PDF_Final_Version_K_and_A.pdf.
206. KENYAPHARMTECH. List of Kenya Pharmaceutical Manufacturers. 2020. Available at URL: https://www.kenyapharmtech.com/kenya-pharmaceutical-manufacturers/.
207. Maisha Meds. COVID consequences: How to spot a drug shortage. 2020. Available at URL: https://maishameds.org/2020/04/28/covid-consequences-how-to-spot-a-drug-shortage/.
208. VUGIGI S, THOITHI GN , JOGAJI JI, ONUONGA SO. Production Capacity of Pharmaceutical Manufacturing Industry in Kenya. East and Central African Journal of Pharmaceutical Sciences. 2017; 20: 3-12.

209. Folorunsho-Francis A. Coronavirus: Drug scarcity imminent by August, Nigerian manufacturers warn. 2020. Available at URL: https://punchng.com/drug-scarcity-imminent-by-august-nigerian-manufacturers-warn/.
210. MSN Africa. Nigeria faces drugs shortage due to Coronavirus, NAFDAC warns. 2020. Available at URL: https://www.msn.com/en-xl/africa/nigeria/nigeria-faces-drugs-shortage-due-to-coronavirus-nafdac-warns/ar-BB106usZ.
211. Medical Brief. Health Department 'taking steps' over shortage of antidepressants and antipsychotics. 2020. Available at URL: https://www.medicalbrief.co.za/archives/health-department-taking-steps-over-shortage-of-antidepressants-and-antipsychotics/.
212. Review Online. No lithium tablets: anti-depressant shortage looms. 2020. Available at URL: https://reviewonline.co.za/404809/no-lithium-tablets-anti-depressant-shortage-looms/.
213. Dabanga. OCHA Sudan: Medical supplies may be affected by Covid-19 measures. 2020. Available at URL: https://www.dabangasudan.org/en/relief-news/article/ocha-sudan-medical-supplies-may-be-affected-by-covid-19-measures.
214. Kivoto PM, Mulaku M, Ouma C, Ferrario A, Kurdi A, Godman B, et al. Clinical and Financial Implications of Medicine Consumption Patterns at a Leading Referral Hospital in Kenya to Guide Future Planning of Care. Frontiers in pharmacology. 2018;9(1348).
215. Alfadl AA, Hassali MA, Ibrahim MI. Counterfeit drug demand: perceptions of policy makers and community pharmacists in Sudan. Research in social & administrative pharmacy. 2013;9(3):302-10.
216. Republic of Zambia Ministry of Health. HEALTH SECTOR SUPPLY CHAIN STRATEGY AND IMPLEMENTATION PLAN (2015 – 2017). 2015. Available at URL: https://www.moh.gov.zm/docs/impplanncss.pdf.
217. Republic of Zambia Ministry of Health. STANDARD TREATMENT GUIDELINES, ESSENTIAL MEDICINES LIST AND ESSENTIAL LABORATORY SUPPLIES LIST FOR ZAMBIA. 2013. Available at URL: https://www.moh.gov.zm/docs/final_standard_treatment_guidelines_booklet_04.pdf.
218. WHO. SDG 3: Ensure healthy lives and promote wellbeing for all at all ages. 2019. Available at URL: https://www.who.int/sdg/targets/en/.
219. UNICEF and WHO. Maintaining routine immunization services vital during the COVID-19 pandemic – WHO and UNICEF. 2020. Available at URL: https://www.unicef.org/northmacedonia/press-releases/maintaining-routine-immunization-services-vital-during-covid-19-pandemic-who-and.
220. Kalungia AC, Mwambula H, Munkombwe D, Marshall S, Schellack N, May C, et al. Antimicrobial stewardship knowledge and perception among physicians and pharmacists at leading tertiary teaching hospitals in Zambia: implications for future policy and practice. Journal of chemotherapy. 2019;31(7-8):378-87.
